# Human Plasma Metabolomics in Age-Related Macular Degeneration: Meta-Analysis of Two Cohorts

**DOI:** 10.3390/metabo9070127

**Published:** 2019-07-02

**Authors:** Inês Laíns, Wonil Chung, Rachel S. Kelly, João Gil, Marco Marques, Patrícia Barreto, Joaquim N. Murta, Ivana K. Kim, Demetrios G. Vavvas, John B. Miller, Rufino Silva, Jessica Lasky-Su, Liming Liang, Joan W. Miller, Deeba Husain

**Affiliations:** 1 Department of Ophthalmology, Massachusetts Eye and Ear, Harvard Medical School, Boston, MA 02114, USA; 2 Faculty of Medicine, University of Coimbra, 3000 Coimbra, Portugal; 3 Centro Hospitalar e Universitário de Coimbra, 3000 Coimbra, Portugal; 4 Association for Innovation and Biomedical Research on Light and Image, 3000 Coimbra, Portugal; 5 Program in Genetic Epidemiology and Statistical Genetics, Harvard T.H. Chan School of Public Health, Boston, MA 02115, USA; 6 Department of Epidemiology, Harvard T.H. Chan School of Public Health, Boston, MA 02115, USA; 7 Systems Genetics and Genomics Unit, Channing Division of Network Medicine Brigham and Women’s Hospital and Harvard Medical School, Boston, MA 02115, USA; 8 Department of Biostatistics, Harvard T.H. Chan School of Public Health, Boston, MA 02115, USA

**Keywords:** age-related macular degeneration, metabolomics, mass spectrometry

## Abstract

The pathogenesis of age-related macular degeneration (AMD), a leading cause of blindness worldwide, remains only partially understood. This has led to the current lack of accessible and reliable biofluid biomarkers for diagnosis and prognosis, and absence of treatments for dry AMD. This study aimed to assess the plasma metabolomic profiles of AMD and its severity stages with the ultimate goal of contributing to addressing these needs. We recruited two cohorts: Boston, United States (*n* = 196) and Coimbra, Portugal (*n* = 295). Fasting blood samples were analyzed using ultra-high performance liquid chromatography mass spectrometry. For each cohort, we compared plasma metabolites of AMD patients versus controls (logistic regression), and across disease stages (permutation-based cumulative logistic regression considering both eyes). Meta-analyses were then used to combine results from the two cohorts. Our results revealed that 28 metabolites differed significantly between AMD patients versus controls (false discovery rate (FDR) *q*-value: 4.1 × 10^−2^–1.8 × 10^−5^), and 67 across disease stages (FDR *q*-value: 4.5 × 10^−2^–1.7 × 10^−4^). Pathway analysis showed significant enrichment of glycerophospholipid, purine, taurine and hypotaurine, and nitrogen metabolism (*p*-value ≤ 0.04). In conclusion, our findings support that AMD patients present distinct plasma metabolomic profiles, which vary with disease severity. This work contributes to the understanding of AMD pathophysiology, and can be the basis of future biomarkers and precision medicine for this blinding condition.

## 1. Introduction

Age-related macular degeneration (AMD) is the leading cause of adult blindness in developed countries. Worldwide, it ranks third, and is expected to affect 288 million people by 2040 [1]. The hallmark of the early phases of AMD are macular drusen and pigmentary changes. Some patients progress to the late forms of the disease, choroidal neovascularization (neovascular form) or geographic atrophy [2,3]. While we have treatment for the neovascular, late form of AMD [4], 90% of those diagnosed with AMD have the early or intermediate forms, for which there is currently no proven therapy [5]. It is likely that the early and intermediate forms of AMD may not represent a single disease, but rather a collection of subtypes that ultimately progress to the advanced forms [6]. Elucidating these subtypes and their underlying pathogenesis will be critical in developing effective therapies for these earlier stages of AMD. Furthermore, any prognostic information is based solely on features identified by ophthalmic examination and/or color fundus imaging, and it is limited. Delay in instituting treatment for neovascular AMD is one of the factors leading to poor visual functional outcomes [7]. Biomarker tools that could improve our prognostic information would fill a crucial, unmet need, and might also provide new insights into AMD pathogenesis. Previous attempts to identify AMD biofluid biomarkers revealed variable results [8,9] probably, in part, due to the complexity of this disease, involving interactions between environmental and genetic risk factors.

Our group hypothesized that metabolomics, the qualitative and quantitative analysis of metabolites (<1–1.5 KDa), could be an appropriate approach to help address these questions [10]. Metabolites are the downstream product of the cumulative effects of the genome and its interaction with environmental exposures; therefore, the metabolome is thought to closely relate to disease phenotype, especially with multifactorial diseases such as AMD [11]. Two main analytical tools are available for metabolomic profiling: nuclear magnetic resonance (NMR) spectroscopy and mass spectrometry (MS) [12]. 

We have published data [13,14] supporting the concept of metabolomics as a powerful tool to identify plasma signatures of AMD and its severity stages. We initiated our work [13] with NMR spectroscopy, based on the principal that it can represent an appropriate technique for an initial untargeted approach. Nuclear magnetic resonance offers high reproducibility, simple sample handling, and the possibility of sample reuse [15,16]. Using this technique [13], we observed small differences between AMD stages, mostly driven by amino acids and some small proteins, organic acids, and lipids. These results motivated us to continue our investigations with MS, which is becoming the most widely used technology for large-scale epidemiological studies [17]. Mass spectrometry has a much higher sensitivity compared to NMR, thus enabling the measurement of a broader range of metabolites [11]. In an MS-based pilot study [14], we identified 87 plasma metabolites that differed significantly between subjects with AMD and controls; most of them were lipids, in particular glycerophospholipids, but differences in some amino acids and dipeptides were also observed. Despite the interesting results, this work was limited by its relatively small sample size (30 controls and 90 subjects with AMD), and the lack of validation in an independent population. 

In this manuscript, we present our analysis of MS plasma metabolomic profiles from a large group of patients with AMD, and a group of subjects older than 50 years with normal macula from two cohorts: one from Boston, United States (USA) and the other from Coimbra, Portugal. Our aim was to further assess and define the plasma metabolomic profiles of AMD and its severity stages, with the ultimate goal of contributing to the current understanding of AMD pathogenic mechanisms, as well as identifying targets that can serve as potential biomarkers and become the basis for precision medicine for this blinding disease.

## 2. Results

### 2.1. Study Population and Identified Plasma Metabolites

**Figure 1:**
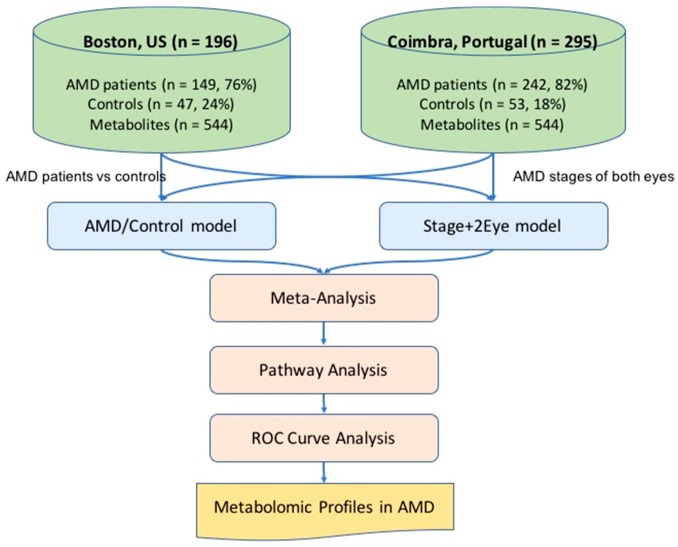
Overview of the study. AMD: age-related macular degeneration; AMD/Control model multivariable logistic regression model considering AMD versus controls as the outcome; Stage+2 Eye: permutation-based cumulative logistic regression model considering both eyes of each patient and the severity stage of disease as the outcome (control, early, intermediate and late); ROC: receiving operating characteristic; *n*: number.

We recruited patients diagnosed with AMD and a control group of subjects with no evidence of AMD and age older than 50 years. A total of 491 subjects were included; 196 (*n* = 149, 76% AMD patients; *n* = 47, 24% controls) in Boston, US and 295 (*n* = 242, 82% AMD patients; *n* = 53, 18% controls) in Coimbra, Portugal. Figure 1 presents the overview of the study. Table 1 presents the clinical and demographic characterization of the included study population. As shown, age differed significantly among groups in both study cohorts (*p*-value < 0.001 for both), and in the Portuguese cohort significant differences were also observed for smoking history among groups (*p*-value = 0.044). 

In both cohorts, 605 metabolites were measured. Sixty-one of these metabolites were determined to be exogenous to humans (e.g., medications, food additives, and buffering agents), and hence, excluded from analysis, as we were interested in endogenous metabolites that could be driving systemic biology. Thus, the final analysis included 544 endogenous metabolites (*n* = 411, identified; *n* = 133, unidentified). Principal component analysis (PCA) analyses were performed to summarize and visualize metabolomic data, and to evaluate how individuals could be clustered based on their metabolomic profiles. Principal components with significant associations are presented in Figure S1 for each cohort. 

### 2.2. Comparison between AMD Patients and Controls 

To assess plasma metabolomic profiles of AMD from our two cohorts, we conducted logistic regression analysis accounting for potential confounders (age, sex, BMI and smoking status) and false discovery rate (FDR). This was first done for the Boston and Coimbra cohorts separately (Tables S1 and S2), and then the results were combined by meta-analysis (see the Methods section: AMD/Control model). The meta-analysis of these results revealed that 69 metabolites differed significantly (*p*-value < 0.05; Table S3) between patients with AMD and controls, 28 of them reaching a statistically significant *q*-value (Table 2). As shown, most of the significant metabolites (*q*-value) were lipids (*n* = 10; 35.7%), followed by amino acids (*n* = 8; 28.6%), nucleotides (*n* = 6; 21.4%), carbohydrates (*n* = 2; 7.1%), cofactors and vitamins (*n* = 1; 3.6%), and peptides (*n* = 1; 3.6%). 

### 2.3. Comparison Across All Stages of Disease

For the comparison across all stages of disease, we used cumulative logistic regression models with likelihood ratio tests (LRTs) by permutation with data on both eyes of each patient (see Methods section: Stage + 2Eye model). This was performed because there was a substantial fraction of patients with different stages of AMD in either eye (15% of the subjects from the Boston cohort and 28% of those from the Coimbra cohort, Table S4). Again, we fitted the models with adjustment for age, BMI, smoking status, and gender for each cohort separately (Tables S5 and S6), and then combined the results by meta-analyses. The meta-analysis of the results revealed that 147 metabolites differed significantly across all stages of disease, based on *p*-value; among which 67 reached a significant *q*-value. As shown, most of these metabolites were lipids (*n* = 34, 50.7%), followed by amino acids (*n* = 20; 30%), nucleotides (*n* = 6, 9.0%), carbohydrates (*n* = 4; 6.0%), cofactors and vitamins (*n* = 1; 1.5%), peptides (*n* = 1; 1.5%) and energy related metabolites (*n* = 1; 1.5%). Table 3 presents the top 20 most significant metabolites, and Table S7 the complete list. 

**Figure 2:**
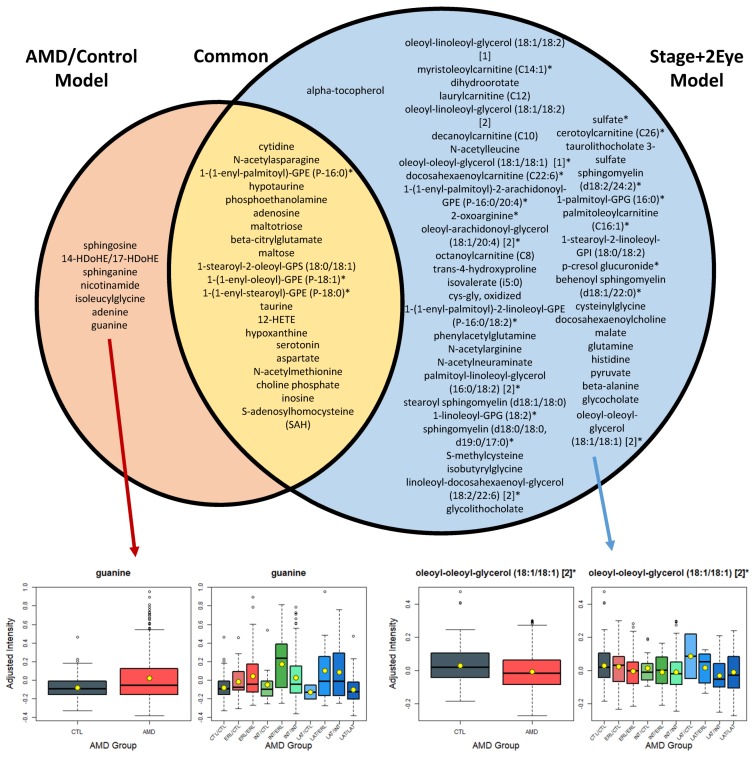
List of the metabolites differing significantly (*q* < 0.05) between AMD patients (AMD/Control model), and controls, and across AMD stages of both eyes (Stage + 2Eye model). AMD: age-related macular degeneration; CTL: control; ERL: early AMD; INT: intermediate AMD; LAT: late AMD. Box plots for the most statistically significant metabolites of each analysis are presented. For each box plot, yellow dots represent the mean and black horizontal lines represent the median; AMD/Control model: multivariable logistic regression model considering AMD versus controls as the outcome; Stage + 2Eye: permutation-based cumulative logistic regression model considering both eyes of each patient and the severity stage of disease as the outcome (control, early, intermediate, and late).

As expected, most of the metabolites identified as differing statistically significantly in the comparison of AMD versus controls were also significantly different in the multi-stage analysis considering all stages of disease (22 out of 28, 79%, Figure 2). 

However, the multi-stage analysis (i.e., considering all stages of disease, Stage+2Eye model) enabled a higher power, and thus, the detection of 45 additional plasma metabolites with different patterns associated with severity of AMD. For example, oleoyl-oleoyl-glycerol 18:1/18:1 (*q*-value in the Stage+2Eye model = 4.2 × 10^−4^) showed distinct levels from controls to the different stages of AMD (Figure 2), which we hypothesized the analysis AMD versus controls did not have sufficient power to demonstrate (*q*-value in the AMD/Control model = 0.21). The full list of the significant metabolites for the analyses of both cohorts and meta-analyses are presented in Tables S8 to S10. Figure S2 presents an additional model for the assessment of stages of disease per individual (i.e., worse eye of each patient considered when differing between eyes) and the common metabolites with the analyses described above (AMD/Control and Stage+2Eye model). Supplementary Tables S11 to S13 present the list of significant metabolites for each cohort and meta-analysis using the Stage model, and Tables S14 to S16 the lists of metabolites described in Supplementary Figure S2 (common among the analyses performed). 

### 2.4. Pathway Analysis

**Figure 3:**
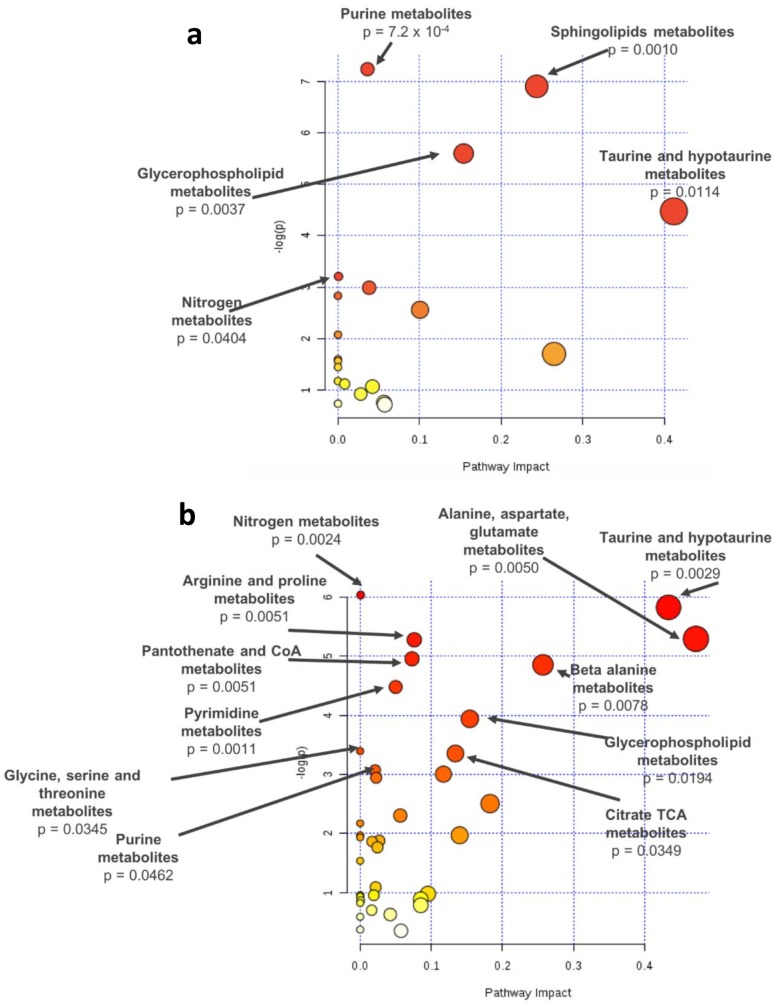
Pathway analysis of the metabolites differing significantly based on *q*-values (**a**) between AMD patients and controls or (**b**) across stages (controls, early AMD, intermediate AMD, and late AMD), identified by meta-analysis of the results of two study cohorts; -log(p): logarithm of the *p*-value.

Pathway analysis of the significant metabolites identified for the comparison among patients with AMD and controls (*q*-value, *n* = 28) revealed a significant enrichment for purine (*p*-value = 7.2 × 10^−4^), sphingolipid (*p*-value = 0.0010), glycerophospholipid (*p*-value = 0.0037), and nitrogen metabolism (*p*-value = 0.0404) (Figure 3a). Pathway analysis based on the metabolites associated with AMD severity stages (Stage+2Eye models; *q*-value, *n* = 67) revealed a statistically significant enrichment of the following pathways: nitrogen (*p*-value = 0.0024), alanine, aspartate, and glutamate (*p*-value = 0.0050), taurine and hypotaurine (*p*-value = 0.0029), arginine and proline (*p*-value = 0.0051), pantothenate and CoA (*p*-value = 0.0051), beta-alanine (*p*-value = 0.0078), pyrimidine (*p*-value = 0.0011), glycerophospholipid (*p*-value = 0.0194), glycine, serine, and threonine (*p*-value = 0.0345), citrate TCA (*p*-value = 0.0349) and purine metabolism (*p*-value = 0.0462) (Figure 3b).

### 2.5. Pathway Analysis

To assess the performance of the models described above (AMD/Control and Stage+2Eye models), we performed receiving operating curve (ROC) assessments. These two models were compared with a model including only demographic covariates (from now on designated baseline model), and another model including the demographic covariates plus the metabolites selected using elastic-net regression with all the plasma metabolites from the study participants (from now on designated All-Met+EN model). In order to avoid overfitting, *p*-values were re-computed only on the training dataset of each fold, and thus, the average numbers of significant metabolites over 10 folds were reduced, as shown in Table 4. 

**Figure 4:**
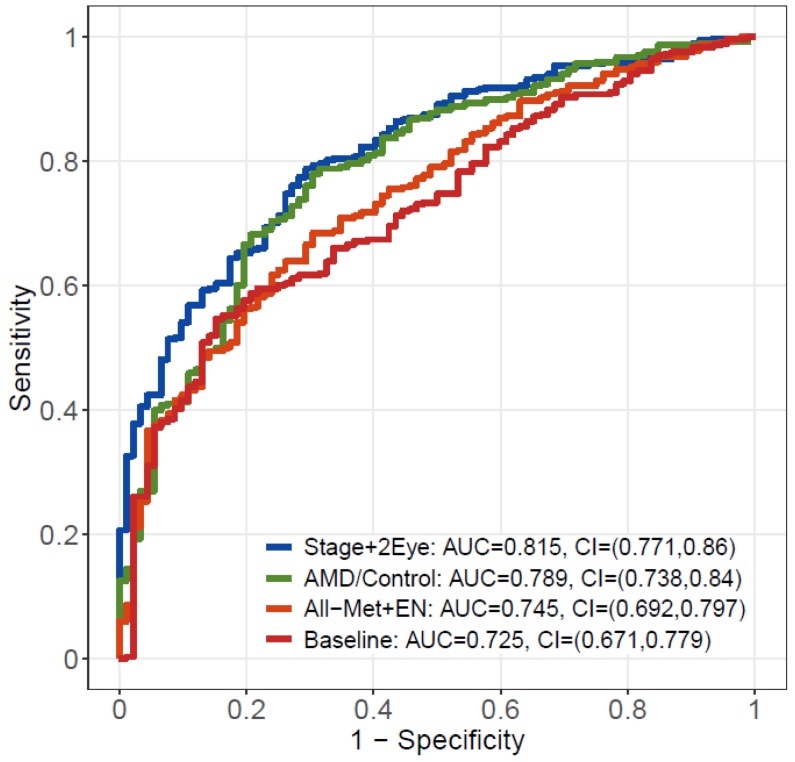
Receiving operating characteristic (ROC) curve analysis from the meta-analysis of the results of the two studies. In red, the baseline model including demographic covariates alone; in orange, the model including demographic covariates plus the metabolites selected by elastic net regression with all metabolites; in green, the model including demographic covariates plus the significant metabolites identified by AMD/Control model; in blue, the model including demographic covariates plus the significant metabolites identified by Stage+2Eye model. AUC – area under the curve; CI – confidence interval; AMD – age-related macular degeneration; Stage+2 Eye – permutation-based cumulative logistic regression model considering both eyes of each patient and the severity stage of disease as the outcome (control, early, intermediate and late); AMD/Control - multivariable logistic regression model considering AMD vs controls as the outcome; All-Met+EN – elastic net regression model with all metabolites; Baseline - statistical model only considering demographic covariates alone.

The AUCs were computed using combined meta-analysis results. Consistently, both the model considering metabolite changes across disease stages (Stage+2Eye model) (AUC = 0.815; 95% CI: 0.771–0.860) and the model comparing patients with AMD versus controls (AMD/Control model) (AUC = 0.789; 95% CI: 0.738–0.840) outperformed (*p*-value = 3.74 × 10^−6^ and *p*-value = 2.07 × 10^−4^, respectively) the baseline model considering demographic covariates alone (AUC = 0.725; 95% CI: 0.671–0.779) (Figure 4). Both models performed better than All-Met+EN model (AUC = 0.745; 95% CI: 0.692–0.797), which performed similarly (*p* = 1.36 × 10^−1^) to the baseline model. Figure S3 shows the results of the AUC assessments separately for the Boston and the Portuguese cohorts, and Figure S4 the performance of the additional model for the assessment of stages of disease per individual (i.e., worse eye of each patient considered when differing between eyes); Table S17 details these results. 

## 3. Discussion

We present a cross-sectional assessment of the plasma metabolomic profiles of 391 subjects with AMD and 100 subjects older than 50 years with a normal macula. Using a state-of-the-art platform for untargeted mass spectrometry, we observed that, after accounting for age, gender, smoking status and BMI, and controlling for false discovery rate, 28 plasma metabolites differed significantly (*q*-value < 0.05) between AMD cases and controls. The majority of these metabolites (*n* = 22, 79%) also differed significantly across severity stages of disease. Most of the identified metabolites were lipids, followed by amino acids and nucleotides. For both comparisons, pathway analysis revealed a significant enrichment of glycerophospholipid metabolism, as well as of purine, taurine, and hypotaurine, and nitrogen metabolism pathways. 

By looking at a larger sample set and using statistical methods to merge and take into account the inclusion of data from two study cohorts, this study validates our initial pilot data [14] on the role of MS metabolomics to identify plasma profiles of AMD and its severity stages. This is important as it further supports how plasma metabolomic profiles may represent future biomarkers for AMD, as shown by ROC curve analysis, where models including metabolites presented an AUC of more than 80% to predict AMD as defined based on color fundus photographs. To this end, further validation on a population-based scale and with targeted quantification of potential metabolic biomarkers is needed [10]. 

This study also supports the relevance of plasma lipid metabolites in AMD, thus adding to the current understanding of their involvement in this condition [18]. In particular, as suggested by our initial data [14], we observed that metabolites mapping to glycerophospholipids pathways are altered in plasma samples of AMD subjects, with differences across severity stages of the disease. As previously discussed by our group [14], glycerophospholipids participate in signal transduction [19,20], and are involved in oxidative stress processes in neurologic disorders [21], namely, in Alzheimer’s disease, which may share important pathogenic mechanisms with AMD. 

We also observed an alteration in the purine metabolism pathway in the plasma of patients with AMD. This has been described by Zhu et al. [22] in a study of long non-coding RNAs, and is in agreement with the relevance of purines, including adenosine triphosphate (ATP), for retinal signaling in general and AMD in particular. Purines are involved in energy metabolism at an intracellular level, but also have extracellular effects through the activation of their receptors (such as P2X and P2Y). Depending on their location, these receptors can affect multiple cellular functions, and are found both in the inner and the outer retina. Namely, through these receptors [23], purinergic signaling seems to play a central role in photoreceptor and RPE cell degeneration [22,23], crucial pathogenic mechanisms of AMD. An over activation of purinergic signaling seems to be involved in this process, and thus contribute to the development of geographic atrophy [23]. The activation of purine receptors in the RPE may also contribute to the accumulation of drusen [24], and to drive the development of choroidal neovascularization [25]. Interestingly, single nucleotide polymorphisms in P2X receptors have been linked with a four-old increased risk of advanced AMD [26], and drugs targeting these receptors have been proposed as potential therapeutic options for geographic atrophy [25,26,27]. 

In this work we also identified significant dysregulation of other pathways related to neurodegeneration, namely of taurine and hypotaurine. Taurine is the most abundant amino acid in the retina [28,29] and its major source is exogenous (i.e., diet). It is transported through the RPE, with a high retinal uptake index [30]. The exact role of taurine in the retina remains unknown, though its neuroprotective functions as an osmolyte and antioxidant have been recognized [31,32] as well as its role (together with hypotaurine) in preventing lipid peroxidation and protection of rod outer segments [33,34]. Taurine is currently seen as a physiological stabilizer of photoreceptor membranes, and studies have shown that, in its absence, there is a degeneration of both ganglion cells, photoreceptors and RPE [29,30]. The observed increased levels of taurine and hypotaurine in the plasma of subjects with AMD may represent a compensatory response to increased levels of lipid peroxidation and oxidative stress [28].

Changes in glutamate (beta-citrylglutamate) and glutamine were also observed in our study between patients with AMD and controls, and across severity stages of disease. The glutamate–glutamine cycle ensures an adequate supply of the neurotransmitter glutamate, which is important for the visual pathway, including the retina. A role for glutamate has been suggested in retinal vein occlusion, glaucoma, and diabetic retinopathy [35,36] through excitotoxicity, leading to neural cell damage or death [35,37]. Importantly, this is thought to be mediated at least in part by oxidative stress [38]. which is an important mechanism in AMD [39]. A recent study on the microbiome of patients with neovascular AMD as compared to controls also observed an enrichment in the microbiome of patients with AMD in genes of glutamate degradation and arginine synthesis [40]. In our study with the four study groups as the outcome (i.e., controls, early, intermediate and late AMD), a dysregulation of arginine metabolites (such as N-acetylarginine and 2-oxoarginine) has also been observed. Further studies are required to clarify these results and a potential relevance of these metabolites in the pathogenesis of AMD. 

Altogether, our data suggest that pathways related to glycerophospholipids and neurodegeneration are altered in the plasma of patients of AMD and may be important in the pathogenesis of this neurodegenerative disease. As previously discussed by our group [14], similar changes have been observed in Alzheimer’s disease for glycerophospholipids, and these seem to represent some of the most consistent blood biomarkers for this condition [41,42,43]. Alterations in both glutamate and purine metabolites [44,45] have also been described in Alzheimer’s disease in other biofluids (such as cerebrospinal fluid). Prior work has proposed that purine metabolism might act as alternative pathway to overcome inadequate glucose supply and energy crisis in neurodegeneration [46]. Of note, Trushina et al. [47] described that approximately 60% of the metabolic pathways altered in the CSF of patients with AD were also affected in plasma of the same individuals, and that the number of common pathways increased with disease severity. 

This study has several limitations. First, we did not analyze the relation between plasma metabolomic findings and dietary patterns and genetic risk profiles of patients and controls. This is extremely relevant considering the nature of metabolomics (reflecting the downstream nature of the genetic transcription process and its interaction with environmental exposures), and because it probably justifies some of the differences observed between our two study cohorts (US and Portugal). Namely, for several metabolites identified on meta-analyses, we observed disparities in effect sizes and statistical significances (Table 2 and 3). This can be related to differences in the sample size of the two cohorts, as in general the larger study cohort (i.e., from Portugal) presented more statistically significant results; however, it can also be explained by potentially different environmental (such as diet) or genetic exposures. Indeed, AMD is a strongly heritable disease [48,49] and metabolomic profiles are controlled by various genetic variants [50], as well as environmental effects, including dietary patterns. This limitation will be addressed in our future work, as we believe that integrative analyses metabolomics-genomics and metabolomics–environmental exposures can provide important insights into AMD pathophysiology and targets for biomarkers’ identification.

Another limitation of our study is the methodology used for AMD classification and staging. We used color fundus photographs, as these remain the gold-standard of AMD grading schemes. However, they miss important features, and have a limited capability to evaluate AMD morphologic changes and to assess disease severity [6]. Indeed, it is increasingly recognized that there is a real need to develop new AMD classification schemes including refined AMD phenotypes as assessed by multimodal imaging [6,51,52]. This would likely affect our results, in particular on the discriminatory ability (i.e., ROC analysis) of the identified plasma metabolomic profiles for two main reasons: (i) other imaging modalities could modify the classification AMD versus no AMD and of the severity stages of disease, because CFP can miss an important number of cases and features [53]; (ii) in these analyses we did not account for the potential added value of imaging biomarkers, in particular of optical coherence tomography features. In the future, it would be interesting to assess if the inclusion of metabolomic profiles also contributes to improve multimodal imaging performance. Still, it is important to note that all included participants underwent a complete ophthalmological exam by an experienced ophthalmologist. This is particularly relevant since many other metabolomic studies rely on established repositories and databases that often lack good phenotypic characterization of ophthalmic diseases. 

The cross-sectional design of this study represents another limitation, as it solely provides a snapshot of the metabolome for the subjects studied. Longitudinal assessments are needed to evaluate the evolution of the metabolome with the progression of the disease. At least for some of the identified significant metabolites (such as guanine, Figure 2), their variation with disease severity did not seem to be linear and warrants further studies. Additionally, in our cohorts, most patients with late AMD had choroidal neovascularization, rather than geographic atrophy, and a comparison between these two advanced forms was not possible. The external validity of our study might also be limited because our cohorts were nearly all Caucasian subjects. This is related in part to the epidemiology of AMD [54], and in part to the population served by both enrolling sites, two tertiary care hospitals. We also did not assess how other parameters, namely, conventional measures of lipid levels (such as serum cholesterol) and other serologic biomarkers, related to our findings. This would be interesting considering the conflicting data on the association between circulating lipoproteins and AMD [18]. Of note, data on smoking and BMI were collected through self-reported questionnaires; thus, there is a potential for response bias. Finally, in this work, we assessed metabolomic profiles of a peripheral biofluid, plasma, and not local eye tissue changes. Therefore, we cannot conclude on how our findings relate to in situ AMD abnormalities. Like in most other peripheral blood biomarker studies, the link between in situ and peripheral metabolomic alterations remains to be elucidated. Methods for metabolomics studies correlating different biospecimens have been developed [55,56,57,58] and should be attempted in the future.

Despite these limitations, to our knowledge, this study represents the largest assessment of plasma metabolomic profiles of AMD patients, including all stages of disease. It is also strengthened by the use of robust statistical methods, namely by accounting for relevant clinical confounding factors, and for multiple comparisons. It is furthermore enhanced by the utilization of information on severity of AMD from both eyes of each subject by performing multivariate cumulative logistic models and by combining two study cohorts by meta-analysis. These methods resulted in the achievement of high statistical power. The detailed assessment of the predictive performance of various models from the two study cohorts proves the usefulness of our approaches. Additionally, this study was prospectively designed, and all study protocols were defined a priori and standardized. Our samples were collected after fasting, processed within 30 min, and immediately stored for metabolomic profiling, which was performed using a state-of-the-art platform that covers a wide-range of the metabolome and identifies metabolites using a chemocentric approach with standards for each identified metabolite.

## 4. Materials and Methods 

### 4.1. Study Design

This study was prospectively designed, and it was an observational, cross-sectional study. Patients were recruited from two sites: Boston, US, at the Department of Ophthalmology of Massachusetts Eye and Ear (MEE), Harvard Medical School; Coimbra, Portugal, at the Faculty of Medicine of the University of Coimbra (FMUC), in collaboration with the Association for Innovation and Biomedical Research on Light and Image (AIBILI) and the “Centro Hospitalar e Universitário de Coimbra”. The clinical protocol was conducted in accordance with HIPAA (Health Insurance Portability and Accountability Act) requirements and the tenets of the Declaration of Helsinki and was approved by the Institutional Review Boards of MEE, FMUC and AIBILI, and by the Portuguese National Data Protection Committee (CNPD). All subjects enrolled in the study provided written informed consent. 

### 4.2. Eligibility Criteria

From January 2015 to July 2016, in both study sites (Coimbra and Boston), we recruited subjects diagnosed with AMD, as well as control subjects with no evidence of AMD and aged ≥ 50 years. At MEE, participants were consecutively recruited from the Retina Service and the Comprehensive Ophthalmology and Optometry Services, at their regular appointments. For those not fasting at that time, a new appointment was scheduled for blood collection within a maximum of one month. The Portuguese (FMUC/AIBILI) study population was derived from a population-based cohort study [59], where all subjects with an established diagnosis of any stage of AMD were invited to participate. Subjects without signs of AMD in a prior evaluation [59] were also invited and included as controls if they remained without the disease in the present evaluation (see criteria below). For both cohorts, the exclusion criteria were: diagnosis of any other vitreoretinal disease, active uveitis or ocular infection, significant media opacities that precluded the observation of the ocular fundus, refractive error equal or greater than 6 diopters of spherical equivalent, past history of retinal surgery, history of any ocular surgery or intra-ocular procedure (such as laser and intra-ocular injections) within the 90 days prior to enrolment, and diagnosis of diabetes mellitus.

### 4.3. Study Protocol

As previously described [13,14], included participants underwent a complete bilateral ophthalmologic examination, including a dilated fundus exam, and were imaged with 7 field, non-stereoscopic color fundus photographs (CFPs), either with a Topcon TRC-50DX (Topcon Corporation, Tokyo, Japan) or a Zeiss FF-450Plus (Carl Zeiss Meditec, Dublin, CA, Ireland) camera. At the same visit, a complete medical history was obtained, according to a standardized questionnaire specifically built for the purposes of this study [60]. This included self-reported data on smoking habits (smokers were considered those who reported current smoking and ex-smokers those who have ever smoked, regardless of when they stopped), and weight and height (used for body mass index, BMI, calculations). If the study participants did not know their current height and/or weight, these were recorded by a study investigator. All data was stored using REDCap electronic data capture tools.

### 4.4. AMD Diagnosis and Staging

For AMD diagnosis and staging, two of three independent experienced graders analyzed field 2 CFPs, according to the Age-Related Eye Disease Study (AREDS) classification system [61,62]. In case of disagreement, a senior author (RS or DH) established the final categorization. Before grading, images were standardized using software developed by our group [63]. Images taken with Topcon cameras were evaluated with IMAGEnet 2000 software (version 2,56; Topcon Medical Systems, Oakland, NJ, USA), and those obtained with a Zeiss camera were observed using VISUPAC (version 4.5.1; Carl Zeiss Meditec, Jena, Germany).

We adopted the most recent AREDS2 definitions [64], namely, that the standard disc diameter equals 1800 µm (rather than 1500 µm), which affects the size of the Early Treatment Diabetic Retinopathy Study (ETDRS) grid and of the standard drusen circles; that geographic atrophy (GA) is present if the lesion has a diameter equal or superior than 433 µm (AREDS circle I-2); and that at least two of the following features are present: absence of retinal pigment epithelium (RPE) pigment, circular shape, or sharp margins (thus, meaning that the involvement of the central fovea is not a requirement). Therefore, the following groups were established and used for further assessments [61,62]: controls—presence of drusen maximum size < circle C0 and total area < C1; early AMD—drusen maximum size ≥ C0 but < C1 or presence of AMD characteristic pigment abnormalities in the inner or central subfields; intermediate AMD—presence drusen maximum size ≥ C1 or of drusen maximum size ≥ C0 if the total area occupied is > I2 for soft indistinct drusen and > O2 for soft distinct drusen; late AMD—presence of GA according to the criteria described above (GA or “dry” late AMD) or evidence of neovascular AMD (choroidal neovascularization, CNV or “wet” AMD).

### 4.5. Sample Collection and Mass Spectrometry Analysis

For all participants, after confirmed overnight fasting, blood samples were collected in the morning, into sodium–heparin tubes, and centrifuged within 30 min (1500 rpm, 10 min, 20 °C). Plasma aliquots of 1.5 mL (MEE) and 5 mL (FMUC/AIBILI) were transferred into sterile cryovials and stored at −80 °C. When all subjects had been recruited, plasma samples from Coimbra, Portugal where shipped to MEE in dry ice (through TNT Express, US, INC). Then, all samples (i.e., from both study locations) were shipped to Metabolon, Inc®, also in dry ice (through TNT® Express, City, State, US, INC). In both cases, samples arrived frozen in less than 48 h and were immediately stored at −80 °C until processing. Non-targeted MS analysis was performed by Metabolon, using ultra-high performance liquid chromatography-tandem MS (UPLC-MS/MS), according to the previously published protocols [14]. The samples from our pilot study (*n* = 120) were analyzed first [14], but all the remaining samples underwent MS analysis simultaneously. To account for a potential batch effect and variation resulting from instrument inter-day differences, Metabolon, Inc® performed data normalization according to their standard protocols; essentially, each compound was corrected in run-day blocks by registering the medians to equal one (1.00) and normalizing each data point (i.e., in our study, two days) proportionately. Then, to merge the first (*n* = 120) and newer (*n* = 371) datasets, we performed normalization by dividing each dataset by the median of the control samples for that study; merged data were then median scaled [64].

### 4.6. Descriptive Statistics and Data Clustering

Traditional descriptive methods were used to describe the clinical and demographic characteristics of the included study population, including mean and standard deviation for continuous variables, and percentages for dichotomous/categorical variables. The four study groups (i.e., controls, early AMD, intermediate AMD, and late AMD) were compared using ANOVA tests. All metabolites were Pareto scaled and log-transformed for statistical assessments. As part of our quality-control procedures, we observed that three subjects (two from Coimbra and one from Boston) had missing or undetectable levels for >30% of metabolites, and therefore, were excluded. Any missing values for the remaining subjects were imputed with half the minimum detected level for that metabolite. To ensure only the most informative metabolites were included in the analysis, those metabolites with an interquartile range of zero were excluded [10].

We applied principal component analysis (PCA) [65] to reduce the dimension of the metabolomics data and to visualize the overall patterns. Principal component analysis is an unsupervised clustering approach that assesses how subjects cluster based on their metabolome. It relies on the transformation of metabolites into a set of linearly uncorrelated variables, known as “principal components”, which summarize a large number of metabolites with a smaller number of variables. This decomposition method maximizes the variance explained by the first component, while the subsequent components explain increasingly reduced amounts of variance [66].

### 4.7. Statistical Methods for Associations between Metabolites and AMD vs. Controls

To identify plasma metabolites associated with AMD case-control status, we used multivariable logistic regression models (from now on designated “AMD/Control models”), with a dichotomous outcome (AMD versus control). These models were computed for each one of our study cohorts separately (i.e., for Boston, US and Coimbra, Portugal). Each metabolite was included in the statistical model as a continuous variable, and we performed adjustments for age, gender, BMI, and smoking status: $logit\left(P\left(Y_{i}=1\right)\right)=\beta_{0}+\beta_{1}X_{i}+\beta_{2}M_{i}$ where *Y_i_* is the binary outcome for AMD status (1: AMD, 0: control) of each individual, *X_i_* is a set of covariates (i.e., age, gender, BMI, and smoke status), and *M_i_* denotes a given plasma metabolite. In this equation, the exp($\beta_{2}$) is the odds ratio (OR) of each metabolite, thus measuring the effect size of a one-unit (i.e., standard deviation) increase in Mi on AMD (versus control). For these analyses, likelihood ratio tests (LRTs) for each metabolite were conducted to compute a *p*-value, because the LRT test is more reliable and powerful than Wald tests in the event of a relatively small to moderate sample size [10]. The significance level for each test was based on the Benjamini–Hochberg procedure controlling for false discovery rate (FDR), which generates FDR adjusted *p*-values (i.e., *q*-values). As mentioned, we obtained *p*-values for the two cohorts (Boston, US and Coimbra, Portugal). Then, to assess both cohorts together, and to achieve a higher statistical power, we performed a meta-analysis. This meta-analysis was based on the Liptak-Stouffer weighted Z-method [67,68], which has a superior power than the Fisher’s combined probability method. Accordingly, we first converted *p*-values for the two cohorts to z-scores and then combined them, assigning different weights to each study according to their sample sizes. For each metabolite, the weighted Z-method is given by $Z_{meta}=\frac{Z_{boston}\sqrt{n_{boston}}+Z_{coimbra}\sqrt{n_{coimbra}}}{\sqrt{n_{boston}+n_{coimbra}}}$. The *p*-values for the meta-analysis were obtained based on two-tailed Z-tests.

### 4.8. Statistical Methods for Associations Between Metabolites and Stages of Disease

To further assess the association between metabolomic plasma profiles and the different stages of AMD, we considered an ordinal outcome: control (0), early (1), intermediate (2), and late AMD (3). Assuming the difference in the log-odds of stage being equal to or below two different categories for each covariate are the same (proportional odds assumption), we performed a proportional-odds cumulative logistic regression model for each metabolite. To evaluate the suitability of these models, we assessed the proportional odds assumption for each covariate and found that the estimated difference in the log-odds of AMD stage for each set of categories of AMD stages was similar for each metabolite or clinical covariate, which indicated that cumulative logistic models fit our data (see Figure S5). Again, these analyses were performed separately for each of the two cohorts, and we adjusted for age, gender, BMI, and smoke status: $logit\left(P\left(Y_{i}\leq j\right)\right)=\beta_{0j}+\beta_{1}X_{i}+\beta_{2}M_{i}$ for j=0,1,2,3. In these models, j is the AMD stage, *Y_i_* is the ordinal outcome for stages (0: control, 1: early, 2: intermediate, 3: late) of each individual, *X_i_* is a set of covariates (i.e., age, gender, BMI, and smoke status), and *M_i_* is a given plasma metabolite. Of note, intercepts for each cumulative logit model can differ but slopes for a metabolite or other covariates stay the same. To further utilize information on both eyes of each patient, we conducted permutation-based cumulative logistic regression models (from now designated “Stage+2Eye model”). For these analyses, we considered the stage of AMD of both eyes of each subject, and thus doubled our sample size. The test statistics were computed using the LRT method. For testing, we generated null distribution of test statistics through shuffling the AMD stages, which resulted in assigning different AMD stage to each set of covariates. Under the estimated null distribution by permutation, we calculated *p*-values for cumulative logistic regression. To combine *p*-values from the two cohorts, we again conducted a meta-analysis based on the Liptak–Stouffer weighted Z-method, as described above. Significance levels were also calculated using FDR, as described for the AMD/Control model.

### 4.9. Pathway Analyses

To interpret the biological relevance of our findings, we performed pathway analyses on the significant metabolites identified from both the AMD/Control and Stage+2Eye models based on *q*-values (i.e., only metabolites with a significant *q*-value were considered). This was performed using Metaboanalyst 4.0 [69], which combines overrepresentation analysis with topology analysis to identify pathways that are dysregulated in AMD based on (i) the number of metabolites from our significant metabolites that fall within Kyoto Encyclopedia of Genes and Genomes (KEGG)-defined metabolic pathways and (ii) the positional importance of our metabolites within these pathways. This analysis generates a pathway impact score and the associated *p*-value. 

### 4.10. Performance Analysis

In order to evaluate the performance of the identified significant metabolites, we used receiver operating characteristic (ROC) curve analyses. For each prediction model, the 10-fold cross validation was used to compute area under the curve (AUC); we divided the dataset from the two cohorts into 10 folds and each fold was used as the validation dataset and the remaining as the training sets. We then conducted a penalized logistic regression (i.e., logistic regression with elastic-net penalty) on the training dataset and obtained the fitted probabilities for AMD status on the validation dataset. The AUC for all models were computed based on AMD status and the fitted probabilities over the 10 folds. We compared the predictive performance among the following four models: (1) Baseline model including only demographic covariates (i.e., age, gender, BMI, and smoking status); (2) All-Met+EN model including baseline and the metabolites selected using elastic-net regression with all metabolites; (3) AMD/Control model including baseline and the metabolites identified in the logistic regression models; (4) Stage+2Eye model including baseline and the metabolites identified in the permutation-based cumulative logistic regression models. Of note, the four demographic covariates were always included, and metabolites were further identified by penalized logistic regression (i.e., elastic net regression) in the final model. For models (3) and (4), only significant metabolites were included based on *p*-values of each model using training datasets (i.e., 90% of data) to avoid overfitting. The AUCs were computed using each cohort separately as well as the combined dataset. All statistical assessments were performed with R (version 3.5.0).

## 5. Conclusions

In conclusion, this work provides additional evidence that patients with AMD present an altered plasma metabolomic profile as compared to controls, and that these profiles vary with disease severity. Our study contributes to the understanding of the pathobiology of AMD, namely, by pointing to the relevance of plasma lipid and amino acid metabolites, in particular, those related to glycerophospholipid, taurine and hypotaurine, purines, as well as nitrogen pathways. Combining these metabolomic data with other phenotypic data (including multimodal imaging and functional testing), as well as genomic and environmental data, may help elucidate AMD subtypes and identify potential druggable targets. Additionally, our findings support that further research should be pursued attempting to develop plasma-based metabolomic biomarkers of AMD. It these are identified in the future, they can contribute to the early diagnosis, screening, and prognosis of this blinding condition. We believe that this work fosters the development of precision medicine in AMD, which can lead to novel interventions based on preventive strategies to reduce progression of this disease, and ultimately to reduce the burden of blindness in AMD.

## 6. Patents

We have submitted a patent entitled “Metabolomics as biomarker for age related macular degeneration”, this is submitted and under review (PCT Patent Application No. PCT/US2018/031878). Patent Filing Institution is Mass Eye and Ear. The authors included on the patent include Ines Lains, Joan Miller, and Deeba Husain. None of the authors have non-financial competing interests.

## Figures and Tables

**Table 1:** Clinical and demographic characterization of the two study cohorts.

**Boston, US**					
**Heading**	**Control**	**Early**	**Intermediate AMD**	**Late**	***p*-Value**
		**AMD**		**AMD**	
Number of patients, *n* (%)	47 (24)	35 (18)	64 (33)	50 (25)	NA
Age, mean ± SD	67.8 ± 8.5	68.5 ± 7.1	72.4 ± 6.9	76.1 ± 8.2	<0.0001 *
BMI, mean ± SD	26.8 ± 4.4	26.7 ± 4.3	27.6 ± 5.6	26.9 ± 4.5	0.779
Gender *n*, (%)					0.564
Female	29 (62)	23 (66)	45 (70)	29 (58)	
Male	18 (38)	12 (34)	19 (30)	21 (42)	
Smoking *n*, (%)					0.107
Non-smoker	24 (52)	21 (60)	27 (42)	17 (35)	
Ex-smoker	20 (43)	14 (40)	34 (53)	31 (66)	
Current smoker	2 (4)	0 (0)	3 (5)	0 (0)	
Race *n*, (%)					0.127
White	39 (95)	30 (91)	63 (98)	44 (94)	
Black	1 (2)	0 (0)	0 (0)	0 (0)	
Asian	0 (0)	1 (3)	1 (2)	0 (0)	
Hispanic	1 (2)	2 (6)	0 (0)	3 (6)	
**Coimbra, Portugal**					
**Heading**	**Control**	**Early**	**Intermediate AMD**	**Late**	***p*-Value**
		**AMD**		**AMD**	
Number of patients, *n* (%)	53 (18)	58 (20)	130 (44)	54 (18)	NA
Age, mean ± SD	68.6 ± 5.0	71.2 ± 6.1	76.6 ± 7.5	81.8 ± 6.9	<0.0001 *
BMI, mean ± SD	27.1 ± 4.7	27.1 ± 4.3	27.4 ± 4.5	26.5 ± 4.3	0.712
Gender *n*, (%)					0.497
Female	35 (66)	35 (60)	90 (69)	32 (59)	
Male	18 (34)	23 (40)	40 (31)	22 (41)	
Smoking *n*, (%)					0.044 *
Non-smoker	43 (81)	50 (86)	116 (89)	39 (72)	
Ex-smoker	10 (19)	8 (14)	14 (11)	14 (26)	
Current smoker	0 (0)	0 (0)	0 (0)	1 (2)	
Race *n*, (%)					0.601
White	53 (100)	58 (100)	128 (98)	53 (98)	
Black	0 (0)	0 (0)	2 (2)	1 (2)	
Asian	0 (0)	0 (0)	0 (0)	0 (0)	
Hispanic	0 (0)	0 (0)	0 (0)	0 (0)	

US: United States; *n*: number of subjects; SD: standard deviation; AMD: age-related macular degeneration. * *p*-value significant based on one-way ANOVA test.

**Table 2:** Metabolites differing significantly (*q*-value) between patients with AMD and controls, based on meta-analysis of the two study cohorts.

Super Pathway	Sub Pathway	Metabolite	OR PT	Pval PT	OR US	Pval US	OR Meta	Pval Meta	Qval Meta
Amino Acid	Methionine, Cysteine, SAM, and Taurine Metabolism	Taurine	3.70	1.19 × 10^−7^	1.26	2.46 × 10^−1^	2.04	9.59 × 10^−7^	6.52 × 10^−5^
Amino Acid	Glutamate Metabolism	Beta−citrylglutamate	2.87	1.37 × 10^−5^	1.52	5.40 × 10^−2^	2.06	3.86 × 10^−6^	2.05 × 10^−4^
Amino Acid	Tryptophan Metabolism	Serotonin	2.00	6.20 × 10^−4^	1.58	2.67 × 10^−2^	1.82	4.84 × 10^−5^	1.65 × 10^−3^
Amino Acid	Methionine, Cysteine, SAM, and Taurine Metabolism	N−acetylmethionine	2.55	1.85 × 10^−5^	1.20	4.05 × 10^−1^	1.75	9.74 × 10^−5^	2.92 × 10^−3^
Amino Acid	Alanine and Aspartate Metabolism	Aspartate	2.37	6.69 × 10^−5^	1.28	2.32 × 10^−1^	1.80	1.02 × 10^−4^	2.92 × 10^−3^
Amino Acid	Methionine, Cysteine, SAM, and Taurine Metabolism	Hypotaurine	2.32	4.69 × 10^−4^	1.36	1.47 × 10^−1^	1.85	2.57 × 10^−4^	6.99 × 10^−3^
Amino Acid	Alanine and Aspartate Metabolism	N−acetylasparagine	1.55	1.96 × 10^−2^	2.00	4.21 × 10^−3^	1.69	3.22 × 10^−4^	7.96 × 10^−3^
Amino Acid	Methionine, Cysteine, SAM, and Taurine Metabolism	S−adenosylhomocysteine (SAH)	2.10	3.91 × 10^−4^	1.24	3.21 × 10^−1^	1.67	6.43 × 10^−4^	1.52 × 10^−2^
Carbohydrate	Glycogen Metabolism	Maltotriose	3.79	2.44 × 10^−7^	1.49	5.12 × 10^−2^	2.47	1.31 × 10^−7^	1.75 × 10^−5^
Carbohydrate	Glycogen Metabolism	Maltose	3.32	1.62 × 10^−6^	1.29	1.86 × 10^−1^	2.14	4.14 × 10^−6^	2.05 × 10^−4^
Cofactors and Vitamins	Nicotinate and Nicotinamide Metabolism	Nicotinamide	2.34	1.91 × 10^−4^	1.15	5.08 × 10^−1^	1.68	7.87 × 10^−4^	1.78 × 10^−2^
Lipid	Phosphatidylserine (PS)	1−stearoyl−2−oleoyl−GPS (18:0/18:1)	5.74	2.94 × 10^−10^	1.23	2.92 × 10^−1^	2.35	1.80 × 10^−8^	6.64 × 10^−6^
Lipid	Phospholipid Metabolism	Phosphoethanolamine	3.97	2.36 × 10^−8^	1.44	5.79 × 10^−2^	2.49	2.44 × 10^−8^	6.64 × 10^−6^
Lipid	Phospholipid Metabolism	Choline phosphate	4.95	4.01 × 10^−9^	1.21	3.21 × 10^−1^	2.38	1.42 × 10^−7^	1.75 × 10^−5^
Lipid	Sphingosines	Sphingosine	16.67	1.89 × 10^−9^	0.99	9.68 × 10^−1^	1.75	2.26 × 10^−6^	1.36 × 10^−4^
Lipid	Lysoplasmalogen	1-(1-enyl-palmitoyl)-GPE (P-16:0) *	1.70	5.86 × 10^−3^	2.47	2.98 × 10^−4^	1.97	1.12 × 10^−5^	4.69 × 10^−4^
Lipid	Fatty Acid, Monohydroxy	14-HDoHE/17-HDoHE	5.02	8.02 × 10^−9^	0.87	4.63 × 10^−1^	1.73	3.87 × 10^−5^	1.46 × 10^−3^
Lipid	Eicosanoid	12-HETE	4.77	2.61 × 10^−8^	0.91	6.26 × 10^−1^	1.64	4.03 × 10^−5^	1.46 × 10^−3^
Lipid	Sphingolipid Synthesis	Sphinganine	5.33	6.18 × 10^−7^	0.90	5.85 × 10^−1^	1.44	3.05 × 10^−4^	7.91 × 10^−3^
Lipid	Lysoplasmalogen	1-(1-enyl-oleoyl)-GPE (P-18:1) *	1.35	9.20 × 10^−2^	2.01	2.36 × 10^−3^	1.60	1.41 × 10^−3^	2.95 × 10^−2^
Lipid	Lysoplasmalogen	1-(1-enyl-stearoyl)-GPE (P-18:0) *	1.25	1.89 × 10^−1^	2.24	6.67 × 10^−4^	1.58	1.81 × 10^−3^	3.65 × 10^−2^
Nucleotide	Purine Metabolism, Adenine containing	Adenosine	2.58	6.33 × 10^−5^	2.15	6.90 × 10^−4^	2.39	1.60 × 10^−7^	1.75 × 10^−5^
Nucleotide	Pyrimidine Metabolism, Cytidine containing	Cytidine	2.55	6.24 × 10^−5^	2.12	1.35 × 10^−3^	2.37	2.93 × 10^−7^	2.66 × 10^−5^
Nucleotide	Purine Metabolism, Guanine containing	Guanine	3.70	5.99 × 10^−7^	1.39	1.08 × 10^−1^	2.40	8.16 × 10^−7^	6.34 × 10^−5^
Nucleotide	Purine Metabolism, (Hypo)Xanthine/Inosine containing	Inosine	2.29	1.54 × 10^−4^	1.65	9.69 × 10^−3^	2.02	4.74 × 10^−6^	2.15 × 10^−4^
Nucleotide	Purine Metabolism, (Hypo)Xanthine/Inosine containing	Hypoxanthine	2.52	3.58 × 10^−5^	1.24	2.79 × 10^−1^	1.80	8.43 × 10^−5^	2.70 × 10^−3^
Nucleotide	Purine Metabolism, Adenine containing	adenine	1.63	2.02 × 10^−2^	1.54	4.38 × 10^−2^	1.58	2.13 × 10^−3^	4.14 × 10^−2^
Peptide	Dipeptide	isoleucylglycine	2.15	1.25 × 10^−4^	1.07	7.26 × 10^−1^	1.58	1.15 × 10^−3^	2.51 × 10^−2^

Legend: OR: odds ratio; PT: Portugal; US: United States; PVal: *p*-value; Qval: *q*-value; Meta: meta-analysis; SAM - S-Adenosyl methionine.

**Table 3:** Top 20 significant metabolites identified on the meta-analysis of the two cohorts for the comparison across all study groups.

Super Pathway	Sub Pathway	Metabolite	OR PT	Pval PT	OR US	Pval US	OR Meta	Pval Meta	Qval Meta
Amino Acid	Alanine and Aspartate Metabolism	N-acetylasparagine	1.23	1.16 × 10^−2^	1.74	1.00 × 10^−6^	1.37	6.10 × 10^−7^	1.65 × 10^−4^
Amino Acid	Methionine, Cysteine, SAM, and Taurine Metabolism	Hypotaurine	1.49	1.00 × 10^−6^	1.19	1.08 × 10^−1^	1.37	1.21 × 10^−6^	1.65 × 10^−4^
Amino Acid	Glutamate Metabolism	Beta−citrylglutamate	1.26	3.40 × 10^−3^	1.46	3.50 × 10^−4^	1.32	6.67 × 10^−6^	4.21 × 10^−4^
Amino Acid	Leucine, Isoleucine, and Valine Metabolism	N-acetylleucine	1.18	4.62 × 10^−2^	1.43	7.30 × 10^−4^	1.27	2.69 × 10^−4^	7.80 × 10^−3^
Carbohydrate	Glycogen Metabolism	Maltotriose	1.43	1.00 × 10^−6^	1.11	2.88 × 10^−1^	1.31	6.19 × 10^−6^	4.21 × 10^−4^
Carbohydrate	Glycogen Metabolism	Maltose	1.38	3.00 × 10^−5^	1.15	1.60 × 10^−1^	1.29	3.17 × 10^−5^	1.72 × 10^−3^
Lipid	Lysoplasmalogen	1-(1-enyl-palmitoyl)-GPE (P-16:0) *	1.21	1.62 × 10^−2^	1.95	1.00 × 10^−6^	1.48	9.86 × 10^−7^	1.65 × 10^−4^
Lipid	Phospholipid Metabolism	Phosphoethanolamine	1.39	2.00 × 10^−5^	1.26	2.01 × 10^−2^	1.32	1.62 × 10^−6^	1.67 × 10^−4^
Lipid	Diacylglycerol	Oleoyl-oleoyl-glycerol (18:1/18:1) [2] *	0.86	5.74 × 10^−2^	0.62	1.00 × 10^−6^	0.76	6.96 × 10^−6^	4.21 × 10^−4^
Lipid	Diacylglycerol	Oleoyl−linoleoyl-glycerol (18:1/18:2) [1]	0.87	7.56 × 10^−2^	0.64	1.00 × 10^−5^	0.76	4.01 × 10^−5^	1.98 × 10^−3^
Lipid	Fatty Acid Metabolism(Acyl Carnitine)	Myristoleoylcarnitine (C14:1) *	0.90	2.06 × 10^−1^	0.62	1.00 × 10^−6^	0.78	6.54 × 10^−5^	2.97 × 10^−3^
Lipid	Phosphatidylserine (PS)	1−stearoyl-2-oleoyl-GPS (18:0/18:1)	1.50	1.00 × 10^−6^	1.01	9.33 × 10^−1^	1.33	8.78 × 10^−5^	3.54 × 10^−3^
Lipid	Fatty Acid Metabolism(Acyl Carnitine)	Laurylcarnitine (C12)	0.91	2.78 × 10^−1^	0.61	1.00 × 10^−6^	0.78	1.18 × 10^−4^	4.29 × 10^−3^
Lipid	Diacylglycerol	Oleoyl-linoleoyl-glycerol (18:1/18:2) [2]	0.89	1.44 × 10^−1^	0.63	2.00 × 10^−5^	0.76	1.68 × 10^−4^	5.67 × 10^−3^
Lipid	Fatty Acid Metabolism(Acyl Carnitine)	Decanoylcarnitine (C10)	0.86	5.97 × 10^−2^	0.69	2.20 × 10^−4^	0.79	1.77 × 10^−4^	5.67 × 10^−3^
Lipid	Lysoplasmalogen	1-(1-enyl-oleoyl)-GPE (P-18:1) *	1.06	4.19 × 10^−1^	1.75	1.00 × 10^−6^	1.32	2.83 × 10^−4^	7.80 × 10^−3^
Lipid	Diacylglycerol	Oleoyl−oleoyl-glycerol (18:1/18:1) [1] *	0.86	5.14 × 10^−2^	0.70	6.50 × 10^−4^	0.79	2.87 × 10^−4^	7.80 × 10^−3^
Nucleotide	Pyrimidine Metabolism, Cytidine containing	Cytidine	1.31	9.10 × 10^−4^	1.69	1.00 × 10^−6^	1.40	1.96 × 10^−8^	1.06 × 10^−5^
Nucleotide	Purine Metabolism, Adenine containing	Adenosine	1.25	3.12 × 10^−3^	1.47	7.00 × 10^−5^	1.32	1.84 × 10^−6^	1.67 × 10^−4^
Nucleotide	Pyrimidine Metabolism, Orotate containing	Dihydroorotate	0.71	1.00 × 10^−6^	0.99	9.44 × 10^−1^	0.80	9.11 × 10^−5^	3.54 × 10^−3^

Legend: OR: odds ratio; PT: Portugal; US: United States; Meta: meta-analysis.

**Table 4:** Area under the curve (AUC) for predictive models for AMD including demographic covariates and significant metabolites identified in the AMD/Control and Stage+2Eye models.

Data	Model	AUC	AUC_CI_L	AUC_CI_U	Nz_Sig	Nz_Final	Pval
Boston,	Baseline	0.645	0.540	0.749	4.0	.	
US	All-Met+EN	0.703	0.602	0.803	544.0	17.2	2.87 × 10^−2^
	AMD/Control	0.691	0.596	0.786	53.0	17.7	2.44 × 10^−1^
	Stage+2Eye	0.747	0.665	0.829	169.5	14.2	1.06 × 10^−2^
Coimbra,	Baseline	0.759	0.697	0.821	4.0	.	
Portugal	All-Met+EN	0.810	0.758	0.862	544.0	15.3	2.27 × 10^−4^
	AMD/Control	0.826	0.775	0.878	57.8	15.1	7.79 × 10^−3^
	Stage+2Eye	0.850	0.803	0.898	87.1	18.6	2.70 × 10^−4^
Combined	Baseline	0.725	0.671	0.779	4.0	.	
	All-Met+EN	0.745	0.692	0.797	544.0	25.5	1.36 × 10^−1^
	AMD/Control	0.789	0.738	0.840	63.7	11.8	2.07 × 10^−4^
	Stage+2Eye	0.815	0.771	0.860	140.6	16.8	3.74 × 10^−6^

AUC_CI_L: Lower bound of 95% confidence interval of AUC; AUC_CI_L: upper bound of 95% confidence interval of AUC; NZ_Sig: number of non-zero significant metabolites selected by logistic or permutation based logistic regression models; NZ_Final: number of non-zero metabolites in the final model; Pval: *p*-value compared to the baseline model; Baseline: baseline model including only demographic covariates; All-Met+EN: all metabolites plus elastic net model including baseline + metabolites selected using elastic net regression with all metabolites; AMD/Control: AMD/Control model including baseline + metabolites identified in the logistic regression; Stage+2Eye: stage+2eye model including baseline + metabolites identified in the permutation-based cumulative logistic regression.

## Supplementary Materials

The following are available online at https://www.mdpi.com/2218-1989/9/7/127/s1, Figure S1: title, PCA Plots and scree plots for 544 metabolites from Boston, US; Figure S2: List of metabolites differing significantly (*q* < 0.05) between patients with AMD and controls (AMD/Control model) and across AMD stages of both eyes (Stage+2Eye model), as well as a supplementary model of AMD stages based on each individual (Stage model) and Coimbra, Portugal cohorts; Figure S3: ROC curves from Boston, US and Coimbra, Portugal cohorts (a) ROC curves for Boston cohort (b) ROC curves for Portuguese cohort; Figure S4: ROC curve analysis of results of the two study cohorts and of the meta-analysis; Figure S5: Proportional odds assumption for the two cohort data (Boston, US and Coimbra, Portugal); Table S1: Metabolites differing significantly between patients with AMD and controls, using Boston (US) samples based on *p*-values of AMD/Control model; Table S2: Metabolites differing significantly between patients with AMD and controls, using Coimbra (Portugal) samples based on p-values of AMD/Control model; Table S3: Metabolites differing significantly (*p*-value) between patients with AMD and controls, based on meta-analysis of AMD/Control model from the 2 study cohorts; Table S4: Characterization of the included study population, by eye; Table S5: Metabolites differing significantly across all study groups using Boston (US) samples based on *p*-values from Stage+2Eye model; Table S6: Metabolites differing significantly across all study groups using Coimbra (Portugal) samples based on *p*-values from Stage+2Eye model; Table S7: Metabolites differing significantly (*p*-value) from Stage+2Eye model identified on the meta-analysis of the 2 cohorts; Table S8: List of the metabolites in Figure 1 and their *p*-values from AMD/Control and Stage+2Eye models from Boston cohort; Table S9: List of the metabolites in Figure 1 and their p-values from AMD/Control and Stage+2Eye models from Portuguese cohort; Table S10: List of the metabolites in Figure 1 and their p-values from AMD/Control and Stage+2Eye models from the meta-analysis; Table S11: Metabolites differing significantly across all study groups using Boston (US) samples based on *p*-values of Stage model; Table S12: Metabolites differing significantly across all study groups using Coimbra (Portugal) samples based on *p*-values of Stage model; Table S13: Metabolites differing significantly (*p*-value) of Stage model across all study groups identified on the meta-analysis of the 2 cohorts; Table S14: List of the metabolites belonging to metabolite sets and their *p*-values of AMD/Control, Stage and Stage+2Eye models from Boston cohort; Table S15: List of the metabolites belonging to metabolite sets and their *p*-values of AMD/Control, Stage and Stage+2Eye models from Portuguese cohort; Table S16: List of the metabolites belonging to metabolite sets and their *p*-values of AMD/Control, Stage and Stage+2Eye models from the meta-analysis; Table S17: Area under the curve (AUC) for predictive models for AMD including clinical covariates and significant metabolites identified in the AMD/Control, Stage, Stage+2Eye or multivariate cumulative logistic models.

## References

[1] Wong W.L., Su X., Li X., Cheung C.M., Klein R., Cheng C.Y., Wong T.Y. (2014). Global prevalence of age-related macular degeneration and disease burden projection for 2020 and 2040: A systematic review and meta-analysis. Lancet Glob. Heal.

[2] Yonekawa Y., Miller J.W., Kim I.K. (2015). Age-Related Macular Degeneration: Advances in Management and Diagnosis. J. Clin. Med..

[3] Miller J.W. (2013). Age-related macular degeneration revisited–piecing the puzzle: The LXIX Edward Jackson memorial lecture. Am. J. Ophthalmol..

[4] Rosenfeld P.J., Brown D.M., Heier J.S., Boyer D.S., Kaiser P.K., Chung C.Y., Kim R.Y., MARINA Study Group (2006). Ranibizumab for Neovascular Age-Related Macular Degeneration. N. Engl. J. Med..

[5] Chew E.Y., Clemons T.E., SanGiovanni J.P., Danis R., Ferris F.L., Elman M., Antoszyk A., Ruby A., Orth D., Bressler S. (2013). Lutein + zeaxanthin and omega-3 fatty acids for age-related macular degeneration: The Age-Related Eye Disease Study 2 (AREDS2) randomized clinical trial. JAMA.

[6] Spaide R.F. (2018). Improving the age-related macular degeneration construct. Retina.

[7] Takahashi H., Ohkubo Y., Sato A., Sato A., Takezawa M., Fujino Y., Yanagi Y., Kawashima H. (2015). Relationship between visual prognosis and delay of intravitreal injection of ranibizumab when treating age-related macular degeneration. Retina.

[8] Kersten E., Paun C.C., Schellevis R.L., Hoyng C.B., Delcourt C., Lengyel I., Peto T., Ueffing M., Klaver C.C.W., Dammeier S. (2018). Systemic and ocular fluid compounds as potential biomarkers in age-related macular degeneration. Surv. Ophthalmol..

[9] Klein R., Myers C.E., Buitendijk G.H.S., Rochtchina E., Gao X., de Jong P.T., Sivakumaran T.A., Burlutsky G., McKean-Cowdin R., Hofman A. (2014). Lipids, Lipid Genes, and Incident Age-Related Macular Degeneration: The Three Continent Age-Related Macular Degeneration Consortium. Am. J. Ophthalmol..

[10] Laíns I., Gantner M., Murinello S., Lasky-Su J.A., Miller J.W., Friedlander M., Husain D. (2018). Metabolomics in the study of retinal health and disease. Prog. Retin. Eye Res..

[11] Nicholson J., Holmes E., Kinross J.M., Darzi A.W., Takats Z., Lindon J.C. (2012). Metabolic phenotyping in clinical and surgical environments. Nature.

[12] Barnes S., Benton H.P., Casazza K., Cooper S.J., Cui X., Du X., Engler J.A., Kabarowski J.H., Li S., Pathmasiri W. (2016). Training in metabolomics research. I. Designing the experiment, collecting and extracting samples and generating metabolomics data. J. Mass Spectrom..

[13] Laíns I., Duarte D., Barros A.S., Martins A.S., Gil J., Miller J.B., Marques M., Mesquita T., Kim I.K., Cachulo M.D.L. (2017). Human plasma metabolomics in age-related macular degeneration (AMD) using nuclear magnetic resonance spectroscopy. PLoS ONE.

[14] Laíns I., Kelly R.S., Miller J.B., Silva R., Vavvas D.G., Kim I.K., Murta J.N., Lasky-Su J., Miller J.W., Husain D. (2017). Human Plasma Metabolomics Study across All Stages of Age-Related Macular Degeneration Identifies Potential Lipid Biomarkers. Ophthalmology.

[15] Psychogios N., Hau D.D., Peng J., Guo A.C., Mandal R., Bouatra S., Sinelnikov I., Krishnamurthy R., Eisner R., Gautam B. (2011). The human serum metabolome. PLoS ONE.

[16] Suhre K., Gieger C. (2012). Genetic variation in metabolic phenotypes: Study designs and applications. Nat. Rev. Genet..

[17] Patti G.J., Yanes O., Siuzdak G. (2012). Innovation: Metabolomics: The apogee of the omics trilogy. Nat. Rev. Mol. Cell Biol..

[18] van Leeuwen E.M., Emri E., Merle B.M.J., Colijn J.M., Kersten E., Cougnard-Gregoire A., Dammeier S., Meester-Smoor M., Pool F.M., de Jong E.K. (2018). A new perspective on lipid research in age-related macular degeneration. Prog. Retin. Eye Res..

[19] Farooqui A.A., Horrocks L.A., Farooqui T. (2000). Glycerophospholipids in brain: Their metabolism, incorporation into membranes, functions, and involvement in neurological disorders. Chem. Phys. Lipids.

[20] Hopiavuori B.R., Agbaga M.-P., Brush R.S., Sullivan M.T., Sonntag W.E., Anderson R.E. (2017). Regional changes in CNS and retinal glycerophospholipid profiles with age: A molecular blueprint. J. Lipid Res..

[21] Farooqui A.A., Horrocks L.A., Farooqui T. (2007). Interactions between neural membrane glycerophospholipid and sphingolipid mediators: A recipe for neural cell survival or suicide. J. Neurosci. Res..

[22] Zhu W., Meng Y.-F., Xing Q., Tao J.J., Lu J., Wu Y. (2017). Identification of lncRNAs involved in biological regulation in early age-related macular degeneration. Int. J. Nanomed..

[23] Reichenbach A., Bringmann A. (2016). Purinergic signaling in retinal degeneration and regeneration. Neuropharmacology.

[24] Guha S., Baltazar G.C., Coffey E.E., Tu L.A., Lim J.C., Beckel J.M., Patel S., Eysteinsson T., Lu W., O’Brien-Jenkins A. (2013). Lysosomal alkalinization, lipid oxidation, and reduced phagosome clearance triggered by activation of the P2X7 receptor. FASEB J..

[25] Carver K.A., Lin C.M., Bowes Rickman C., Yang D. (2017). Lack of the P2X7 receptor protects against AMD-like defects and microparticle accumulation in a chronic oxidative stress-induced mouse model of AMD. Biochem. Biophys. Res. Commun..

[26] Gu B.J., Baird P.N., Vessey K.A., Skarratt K.K., Fletcher E.L., Fuller S.J., Richardson A.J., Guymer R.H., Wiley J.S. (2013). A rare functional haplotype of the *P2RX4* and *P2RX7* genes leads to loss of innate phagocytosis and confers increased risk of age-related macular degeneration. FASEB J..

[27] Yang D., Elner S.G., Clark A.J., Hughes B.A., Petty H.R., Elner V.M. (2011). Activation of P2X receptors induces apoptosis in human retinal pigment epithelium. Invest. Ophthalmol. Vis. Sci..

[28] Ripps H., Shen W. (2012). Review: Taurine: A “very essential” amino acid. Mol. Vis..

[29] Hadj-Saïd W., Froger N., Ivkovic I., Jiménez-López M., Dubus É., Dégardin-Chicaud J., Simonutti M., Quénol C., Neveux N., Villegas-Pérez M.P. (2016). Quantitative and Topographical Analysis of the Losses of Cone Photoreceptors and Retinal Ganglion Cells Under Taurine Depletion. Investig. Opthalmol. Vis. Sci..

[30] Gaucher D., Arnault E., Husson Z., Froger N., Dubus E., Gondouin P., Dherbécourt D., Degardin J., Simonutti M., Fouquet S. (2012). Taurine deficiency damages retinal neurones: Cone photoreceptors and retinal ganglion cells. Amino Acids.

[31] Tomi M., Terayama T., Isobe T., Egami F., Morito A., Kurachi M., Ohtsuki S., Kang Y.S., Terasaki T., Hosoya K. (2007). Function and regulation of taurine transport at the inner blood–retinal barrier. Microvasc. Res..

[32] Ando D., Kubo Y., Akanuma S., Egami F., Morito A., Kurachi M., Ohtsuki S., Kang Y.S., Terasaki T., Hosoya K. (2012). Function and regulation of taurine transport in Müller cells under osmotic stress. Neurochem. Int..

[33] Pasantes-Morales H., Cruz C. (1985). Taurine: A physiological stabilizer of photoreceptor membranes. Prog. Clin. Biol. Res..

[34] Pasantes-Morales H., Cruz C. (1985). Taurine and hypotaurine inhibit light-induced lipid peroxidation and protect rod outer segment structure. Brain Res..

[35] Ishikawa M. (2013). Abnormalities in Glutamate Metabolism and Excitotoxicity in the Retinal Diseases. Scientifica.

[36] Ahsan H. (2015). Diabetic retinopathy--biomolecules and multiple pathophysiology. Diabetes Metab. Syndr..

[37] Boldyrev A., Bulygina E., Makhro A. (2004). Glutamate receptors modulate oxidative stress in neuronal cells. A mini-review. Neurotox. Res..

[38] Coyle J.T., Puttfarcken P. (1993). Oxidative stress, glutamate, and neurodegenerative disorders. Science.

[39] George A.K., Singh M., Homme R.P., Majumder A., Sandhu H.S., Tyagi S.C. (2018). A hypothesis for treating inflammation and oxidative stress with hydrogen sulfide during age-related macular degeneration. Int. J. Ophthalmol..

[40] Zinkernagel M.S., Zysset-Burri D.C., Keller I., Berger L.E., Leichtle A.B., Largiadèr C.R., Fiedler G.M., Wolf S. (2017). Association of the Intestinal Microbiome with the Development of Neovascular Age-Related Macular Degeneration. Sci. Rep..

[41] Farooqui A.A. (2012). Lipid mediators and their metabolism in the nucleous: Implications for Alzheimer’s disease. J. Alzheimers Dis..

[42] Frisardi V., Panza F., Seripa D., Farooqui T., Farooqui A.A. (2011). Glycerophospholipids and glycerophospholipid-derived lipid mediators: A complex meshwork in Alzheimer’s disease pathology. Prog. Lipid Res..

[43] Mapstone M., Cheema A.K., Fiandaca M.S., Zhong X., Mhyre T.R., MacArthur L.H., Hall W.J., Fisher S.G., Peterson D.R., Haley J.M. (2014). Plasma phospholipids identify antecedent memory impairment in older adults. Nat. Med..

[44] Jové M., Portero-Otín M., Naudí A., Ferrer I., Pamplona R. (2014). Metabolomics of Human Brain Aging and Age-Related Neurodegenerative Diseases. J. Neuropathol. Exp. Neurol..

[45] Kaddurah-Daouk R., Zhu H., Sharma S., Ferrer I., Pamplona R. (2013). Alterations in metabolic pathways and networks in Alzheimer’s disease. Transl. Psychiatry.

[46] Kori M., Aydın B., Unal S., Arga K.Y., Kazan D. (2016). Metabolic Biomarkers and Neurodegeneration: A Pathway Enrichment Analysis of Alzheimer’s Disease, Parkinson’s Disease, and Amyotrophic Lateral Sclerosis. Omics J. Integr. Biol..

[47] Trushina E., Dutta T., Persson X.-M.T., Mielke M.M., Petersen R.C. (2013). Identification of Altered Metabolic Pathways in Plasma and CSF in Mild Cognitive Impairment and Alzheimer’s Disease Using Metabolomics. PLoS ONE.

[48] Fritsche L.G., Igl W., Bailey J.N.C., Grassmann F., Sengupta S., Bragg-Gresham J.L., Burdon K.P., Hebbring S.J., Wen C., Gorski M. (2016). A large genome-wide association study of age-related macular degeneration highlights contributions of rare and common variants. Nat. Genet..

[49] Fritsche L.G., Chen W., Schu M., Yaspan B.L., Yu Y., Thorleifsson G., Zack D.J., Arakawa S., Cipriani V., Ripke S. (2013). Seven new loci associated with age-related macular degeneration. Nat. Genet..

[50] Kraus W.E., Muoio D.M., Stevens R., Craig D., Bain J.R., Grass E., Haynes C., Kwee L., Qin X., Slentz D.H. (2015). Metabolomic Quantitative Trait Loci (mQTL) Mapping Implicates the Ubiquitin Proteasome System in Cardiovascular Disease Pathogenesis. PLoS Genet..

[51] Miller J.W., Bagheri S., Vavvas D.G. (2017). Advances in Age-related Macular Degeneration Understanding and Therapy. US Ophthalmic Rev..

[52] Spaide R.F. (2017). Disease expression in nonexudative age-related macular degeneration varies with choroidal thickness. Retina.

[53] Neely D.C., Bray K.J., Huisingh C.E., Clark M.E., McGwin G., Owsley C. (2017). Prevalence of Undiagnosed Age-Related Macular Degeneration in Primary Eye Care. JAMA Ophthalmol..

[54] Sobrin L., Seddon J.M. (2013). Nature and nurture- genes and environment- predict onset and progression of macular degeneration. Prog. Retin. Eye Res..

[55] Do K.T., Pietzner M., Rasp D.J., Friedrich N., Nauck M., Kocher T., Suhre K., Mook-Kanamori D.O., Kastenmüller G., Krumsiek J. (2017). Phenotype-driven identification of modules in a hierarchical map of multifluid metabolic correlations. NPJ Syst. Biol. Appl..

[56] Do K.T., Kastenmüller G., Mook-Kanamori D.O., Yousri N.A., Theis F.J., Suhre K., Krumsiek J. (2015). Network-Based Approach for Analyzing Intra- and Interfluid Metabolite Associations in Human Blood, Urine, and Saliva. J. Proteome Res..

[57] Feng Q., Liu Z., Zhong S., Li R., Xia H., Jie Z., Wen B., Chen X., Yan W., Fan Y. (2016). Integrated metabolomics and metagenomics analysis of plasma and urine identified microbial metabolites associated with coronary heart disease. Sci. Rep..

[58] Xia J., Wishart D.S. (2010). MSEA: A web-based tool to identify biologically meaningful patterns in quantitative metabolomic data. Nucleic Acids Res..

[59] da Luz Cachulo M., Lobo C., Figueira J., Ribeiro L., Laíns I., Vieira A., Nunes S., Costa M., Simão S., Rodrigues V. (2015). Prevalence of Age-Related Macular Degeneration in Portugal: The Coimbra Eye Study—Report 1. Ophthalmologica.

[60] Laíns I., Miller J.B., Mukai R., Mach S., Vavvas D., Kim I.K., Miller J.W., Husain D. (2017). Health conditions linked to age-related macular degeneration associated with dark adaptation. Retina.

[61] (2001). The Age-Related Eye Disease Study system for classifying age-related macular degeneration from stereoscopic color fundus photographs: The Age-Related Eye Disease Study Report Number 6. Am. J. Ophthalmol..

[62] Danis R.P., Domalpally A., Chew E.Y., Clemons T.E., Armstrong J., SanGiovanni J.P., Ferris F.L. (2013). Methods and reproducibility of grading optimized digital color fundus photographs in the Age-Related Eye Disease Study 2 (AREDS2 Report Number 2). Invest. Ophthalmol. Vis. Sci..

[63] Tsikata E., Laíns I., Gil J.Q., Marques M., Brown K., Mesquita T., Melo P., Cachulo M.D.L., Kim I.K., Vavvas D. (2017). Automated Brightness and Contrast Adjustment of Color Fundus Photographs for the Grading of Age-Related Macular Degeneration. Transl. Vis. Sci. Technol..

[64] Li B., Tang J., Yang Q., Cui X., Li S., Chen S., Cao Q., Xue W., Chen N., Zhu F. (2016). Performance Evaluation and Online Realization of Data-driven Normalization Methods Used in LC/MS based Untargeted Metabolomics Analysis. Sci. Rep..

[65] Barnes S., Benton H.P., Casazza K., Cooper S.J., Cui X., Du X., Engler J., Kabarowski J.H., Li S., Pathmasiri W. (2016). Training in metabolomics research. II. Processing and statistical analysis of metabolomics data, metabolite identification, pathway analysis, applications of metabolomics and its future. J. Mass Spectrom..

[66] Yin P., Peter A., Franken H., Zhao X., Neukamm S.S., Rosenbaum L., Lucio M., Zell A., Haring H.-U., Xu G. (2013). Preanalytical aspects and sample quality assessment in metabolomics studies of human blood. Clin. Chem..

[67] Zaykin D.V. (2011). Optimally weighted Z-test is a powerful method for combining probabilities in meta-analysis. J. Evol. Biol..

[68] Whitlock M. (2005). C Combining probability from independent tests: The weighted Z-method is superior to Fisher’s approach. J. Evol. Biol..

[69] Chong J., Soufan O., Li C., Caraus I., Li S., Bourque G., Wishart D.S., Xia J. (2018). MetaboAnalyst 4.0: Towards more transparent and integrative metabolomics analysis. Nucleic Acids Res..

