# Supplementary material for: Human Plasma Metabolomics in Age-Related Macular Degeneration: Meta-Analysis of Two Cohorts
Source: Metabolites. 2019 Jul 2;9(7):127. doi: 10.3390/metabo9070127 (PMC6680405; doi:10.3390/metabo9070127)
Supplement: Supplementary file 1 [file metabolites-09-00127-s001.pdf]

Supplementary Information

Human Plasma Metabolomics in Age-related Macular Degeneration – Meta-Analysis of Two Cohorts

Inês Laíns, MD[1,2,3,4†], Wonil Chung, PhD[5,6†], Rachel S. Kelly, PhD[7], João Gil, MD[2], Marco Marques, MD[2], Patrícia Barreto, MSc[4], Joaquim N. Murta, MD [2,3,4], Ivana K. Kim, MD[1], Demetrios G. Vavvas, MD[1], John B. Miller, MD[1], Rufino Silva, MD[2,3,4], Jessica Lasky-Su, PhD[7], Liming Liang, PhD[5,6,8#], Joan W. Miller, MD[1#] and Deeba Husain, MD[1#*]

Supplementary Figure 1. PCA Plots and scree plots for 544 metabolites from Boston, US and Coimbra, Portugal cohorts. For figures a, b, d and e, the x-axis corresponds to PC 1 and y-axis corresponds to PC 2. Each axis shows the variance explained by individual PC and p-value for association with AMD vs. control (a, d) or three AMD stages (b, e). For figures c and f, the green line on top shows the accumulated variance explained and the blue line underneath represents the variance explained by individual PC.

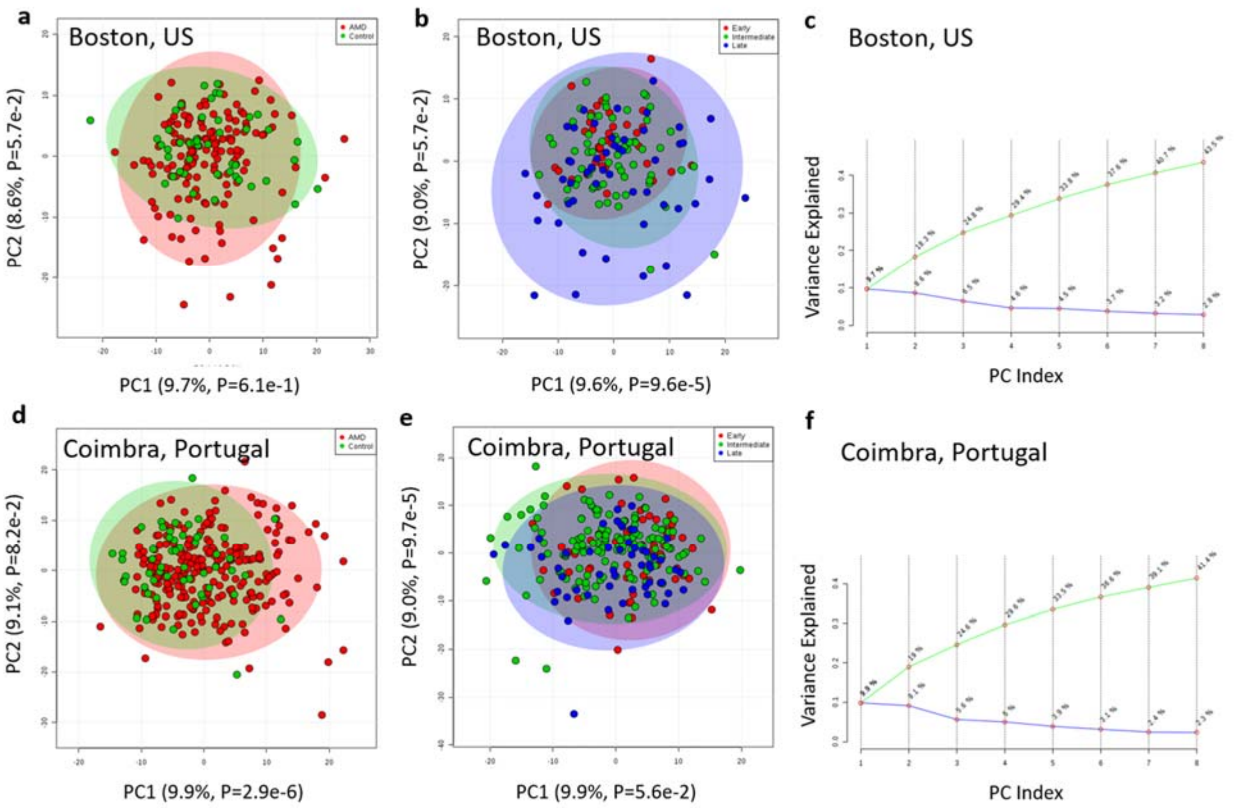

Legend: PC – principal component. For the Boston cohort, the first 12 PCs explained about 50% of the variation, among which PCs 3 and 5 were significantly associated with AMD (as compared to controls) and PCs 2, 4, 7, 8 and 9 were significantly associated with AMD stages (early, intermediate and late). For the Coimbra cohort, about 50% of the variation was explained by the first 13 PCs, among which PCs 1, 3, 4, 5, 6, 8 and PCs 2, 4, 7, 8, 9 were significantly associated with AMD vs. control or AMD stages, respectively.

Supplementary Figure 2. List of metabolites differing significantly (q<0.05) between patients with AMD and controls (AMD/Control model) and across AMD stages of both eyes (Stage+2Eye model), as well as a supplementary model of AMD stages based on each individual (Stage model).

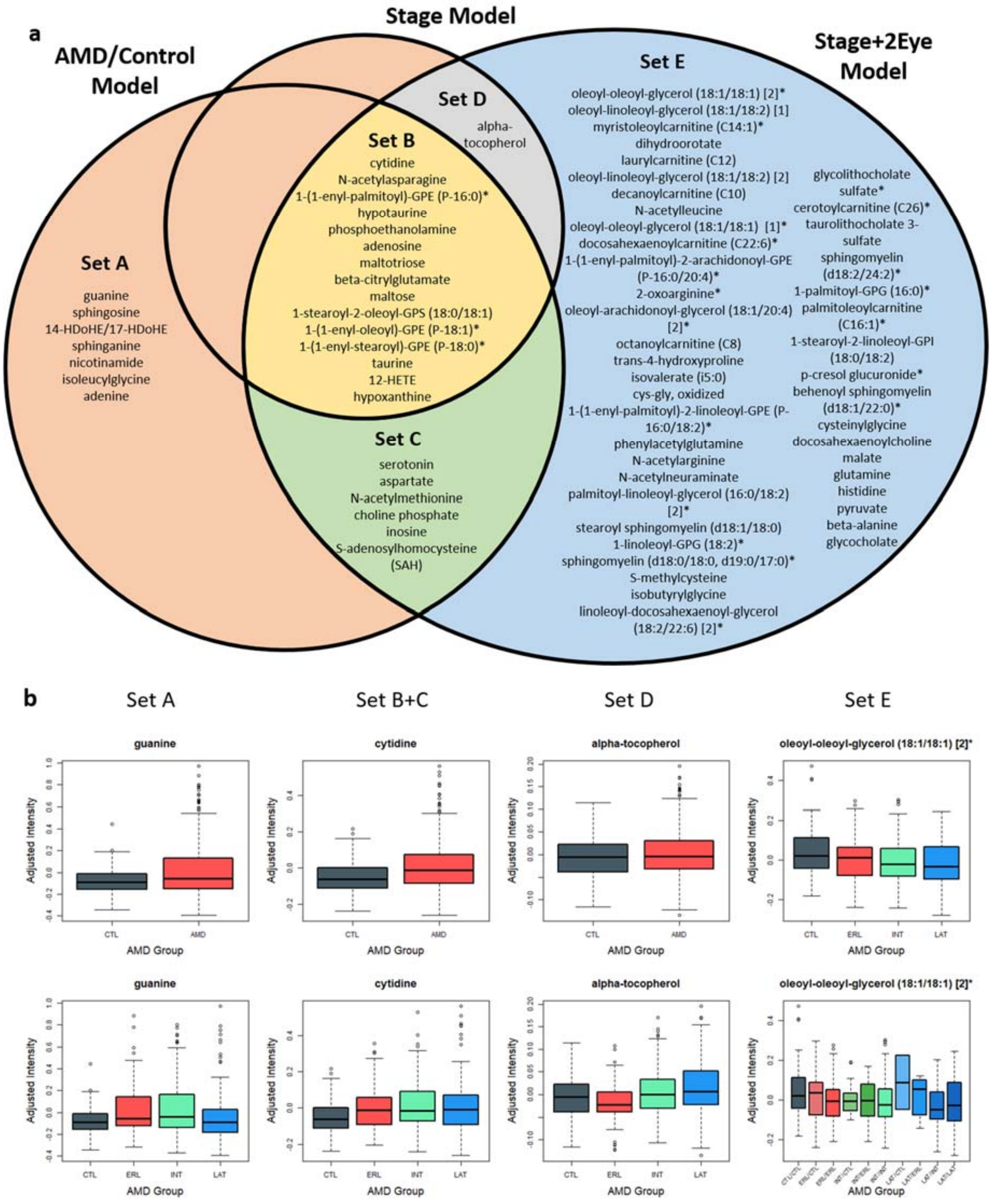

Legend: AMD – age related macular degeneration; CTL – control; ERL – early AMD; INT – intermediate AMD; LAT – late AMD. For each box plot, yellow dots represent the mean and black horizontal lines represent the median. In addition to the AMD/Control model (i.e. multivariable logistic regression model considering AMD vs controls as the outcome) and the Stage+2 Eye (i.e. permutation-based cumulative logistic regression model considering both eyes of each patient and using severity stage of disease as the outcome (control, early, intermediate and late)), we present an additional model (cumulative logistic regression model) also having as the outcome stages of disease (control, early, intermediate and late) but per individual and not accounting for each eye separately. For this model (similarly to the AMD/Control model) if the two eyes of each individual presented a different AMD stage the worst was considered. The list of metabolites differing significantly between AMD and controls based on q-value is presented on metabolite set A+B+C. Considering the Stage model, these can be divided into two groups (i.e. set A vs. set B+C) that contain metabolites with the significantly different adjusted intensity between AMD vs. controls but with no significant linear trends across stages (set A), or with significant linear patterns across stages (set B+C). The list of identified metabolites based on q-value using all stages of AMD is presented above (set B+D). Only one metabolite (i.e. alpha-tocopherol) was additionally detected using the Stage model (Supplementary Tables 11-13). Using AMD stages of both eyes, we identified an additional set of metabolites (set E) that had similar levels between AMD vs. controls but significant linear trends within AMD stages. The full lists of the significant metabolites for both cohort analyses and meta-analyses are presented in Supplementary Tables 14-16.

Supplementary Figure 3. ROC curves from Boston, US and Coimbra, Portugal cohorts (a) ROC curves for Boston cohort (b) ROC curves for Portuguese cohort.

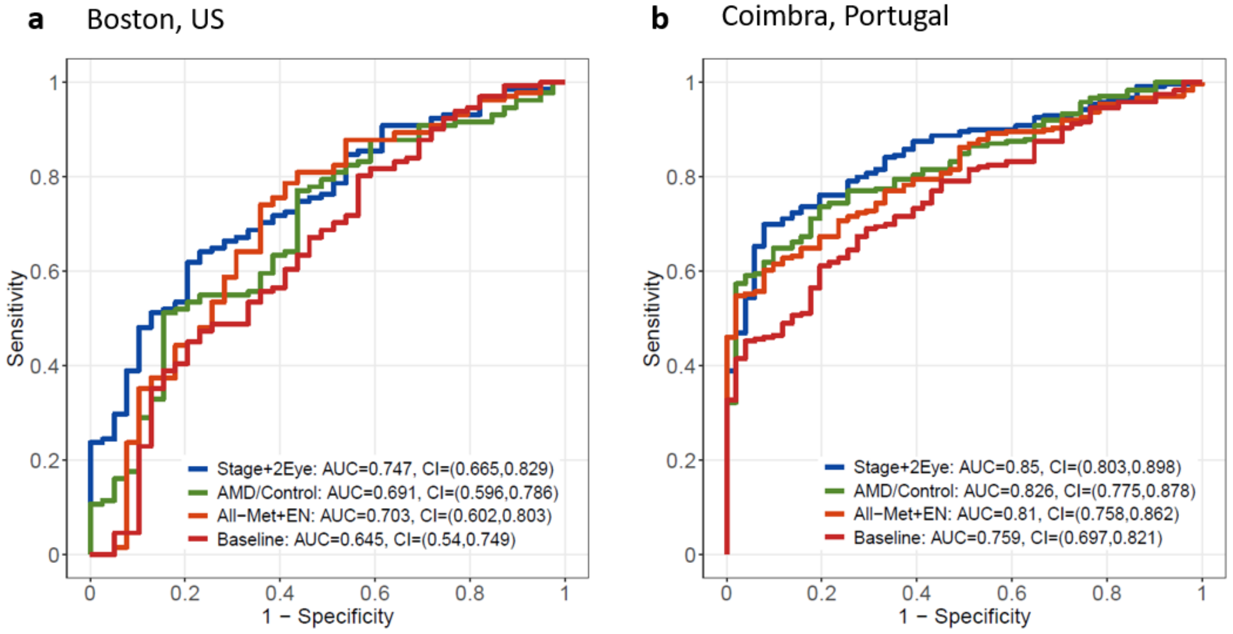

Legend: ROC – receiving operator characteristic; AUC – area under the curve; CI – confidence interval. For the Boston cohort, Stage+2Eye model (i.e. disease stages of both eyes; blue) and AMD/Control model (i.e. AMD vs controls, green) outperformed ($p=1.06\times10^{-2}$, $p=2.44\times10^{-1}$, respectively) the baseline model (red) with demographic covariates alone. Both models performed better than All-Met+EN model (i.e. elastic net with all metabolites, orange), which performed better ($p=2.87\times10^{-2}$) than the baseline model with demographic covariates only. Similarly, for the Coimbra cohort, Stage+2Eye model (i.e. disease stages of both eyes; blue) and AMD/Control model (i.e. disease stages of both eyes; green) performed significantly better ($p=2.70\times10^{-4}$, $p=7.79\times10^{-3}$, respectively) than the baseline model with demographic covariates (red). Also, both models performed better than All-Met+EN model (orange), which outperformed ($p=2.27\times10^{-4}$) than the baseline model (red). In general, AUC for the Portuguese cohort were larger than AUC for the US cohort.

Supplementary Figure 4. ROC curve analysis of results of the two study cohorts and of the meta-analysis. In addition to the models presented in Supplementary Figure 3 and Figure 3, the Stage model considering severity stages by individual is also presented.

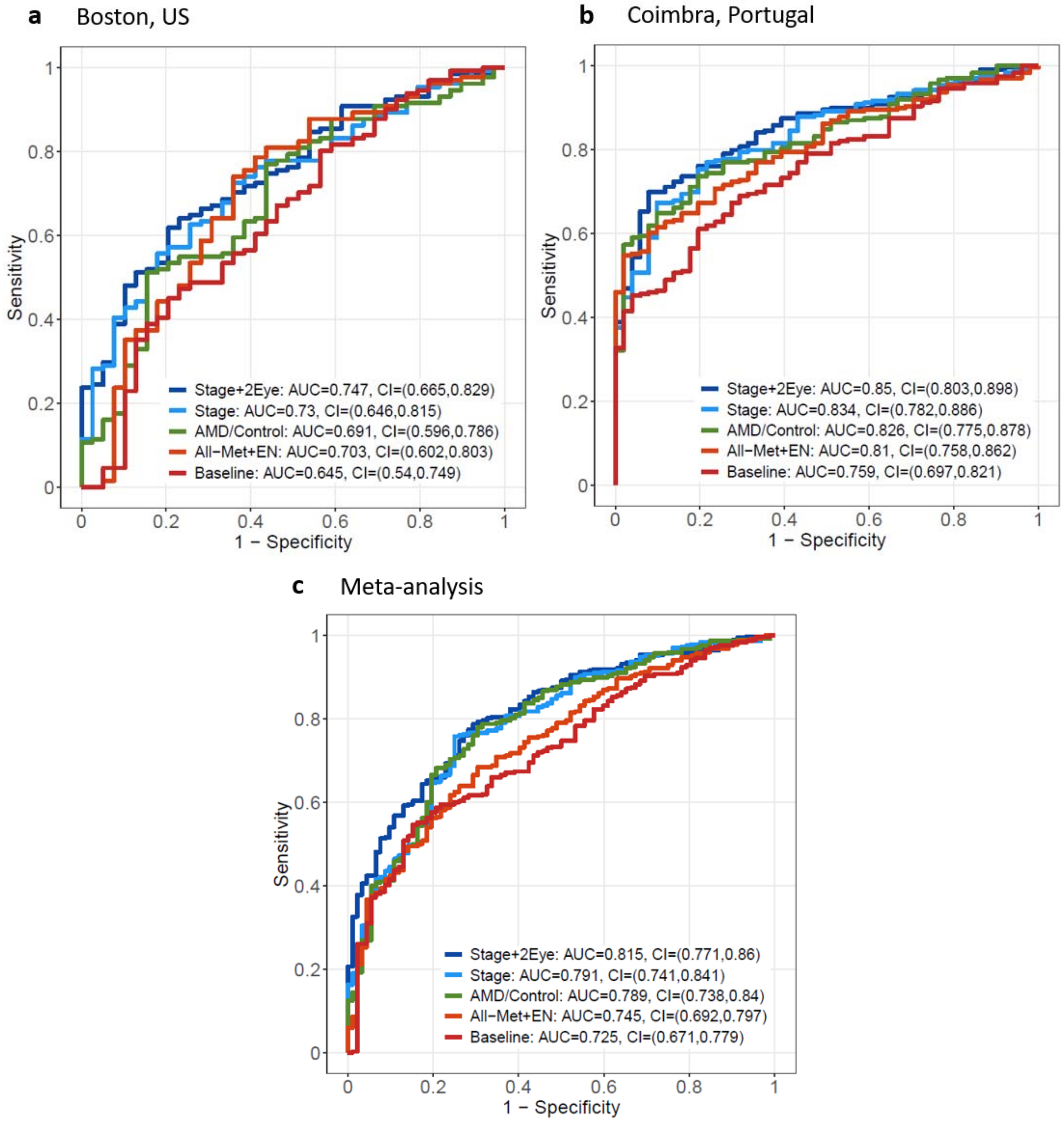

Legend: ROC – receiving operator characteristic; AUC – area under the curve; CI – confidence interval. For the Boston, US cohort, Stage+2Eye model (i.e. disease stages of both eyes; dark blue), Stage model (disease stages per individual, light blue) and AMD/Control model (i.e. AMD vs controls, green) outperformed ($p=1.06\times10^{-2}$, $p=4.06\times10^{-2}$, $p=2.44\times10^{-1}$) the baseline model with demographic covariates alone (red). All three models performed better than All-Met+EN model (i.e. elastic net with all metabolites, orange), which performed better ($p=2.87\times10^{-2}$) than the baseline model with demographic covariates only. Similarly, for the Coimbra, Portugal cohort, Stage+2Eye model

(i.e. disease stages of both eyes; dark blue), Stage model (disease stages per individual, light blue) and AMD/Control model (i.e. AMD vs controls, green) performed significantly better ($p=2.70\times10^{-4}$, $p=1.26\times10^{-3}$, $p=7.79\times10^{-3}$) than the baseline model with demographic covariates alone (red). The Stage model performed better than AMD/Control model, as it used AMD stages, but worse than Stage+2Eye, as it used only AMD stages of each subject rather than information on each eye. Also, all three models performed better than All-Met+EN model (orange), which outperformed ($p=2.27\times10^{-4}$) the baseline model (red). AUC were also computed using combined meta-analysis results. Stage+2Eye model (i.e. disease stages of both eyes; dark blue), Stage model (disease stages of severe eye; light blue) and AMD/Control model (i.e. AMD vs controls; green) outperformed ($p=3.74*10^{-6}$, $p=1.65*10^{-4}$, $p=2.07*10^{-4}$) the baseline model (red). And all three models performed better than All-Met+EN model (orange), which performed similarly ($p=1.36\times10^{-1}$) to the baseline model (red) (see Supplementary Table 17 for more details).

Supplementary Figure 5. Proportional odds assumption for the two cohort data (Boston, US and Coimbra, Portugal). For each predictor variable (including 4 clinical covariates and 544 metabolites), distance between the symbols for each set of categories of the dependent variable remains similar and thus the proportional odds assumption holds.

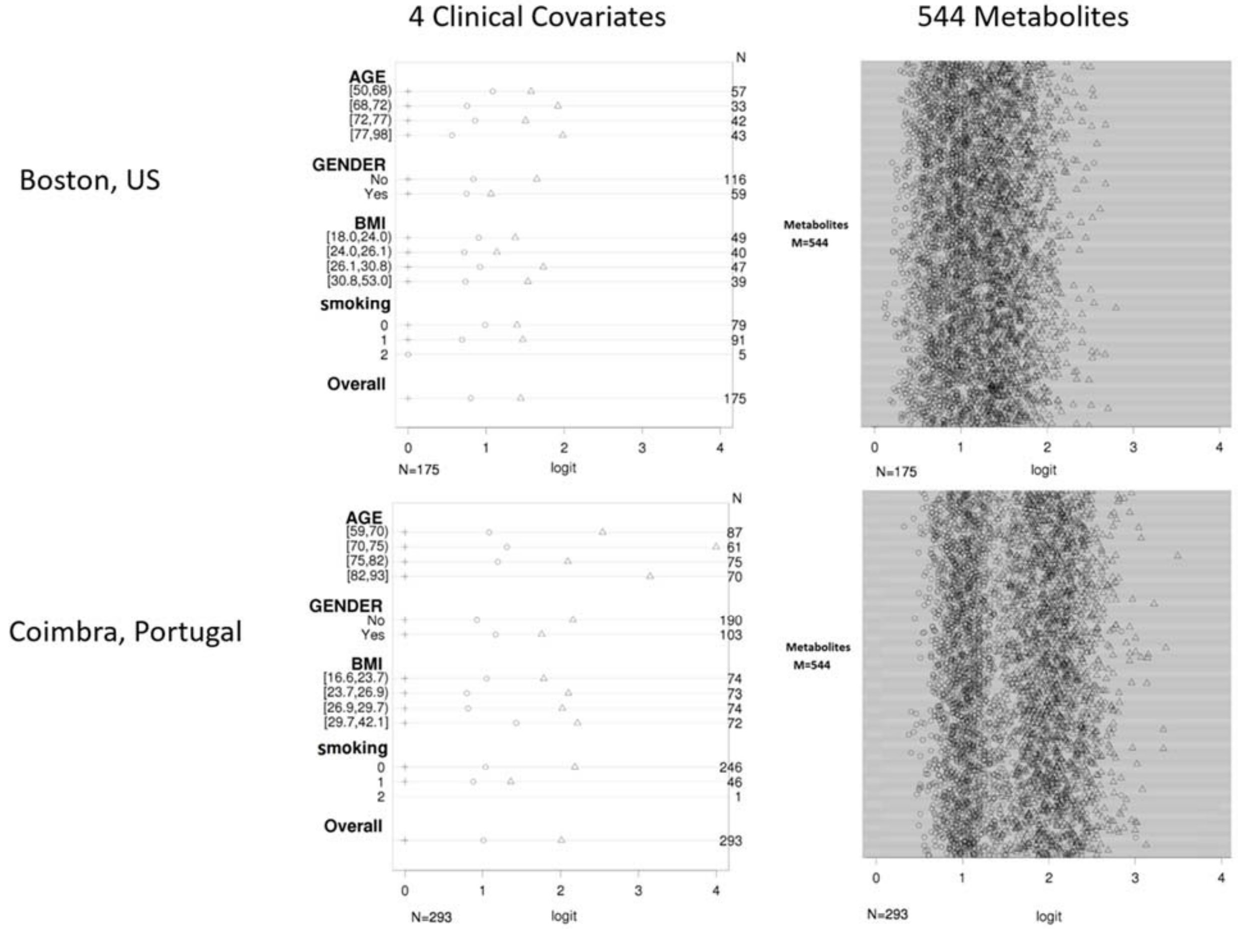

To evaluate suitability of the model, we checked the proportional odds assumption for each covariate and found that estimated difference in log-odds of AMD stage for each set of categories of AMD stages were similar for each metabolite or clinical covariate, which indicated the cumulative logistic model fits our data.

Supplementary Table 1. Metabolites differing significantly between patients with AMD and controls, using Boston (US) samples based on p-values of AMD/Control model.

Super Pathway	Sub Pathway	Metabolite	OR US	Pval US	Qval US	Qval Sig
Amino Acid	Methionine, Cysteine, SAM and Taurine Metabolism	cysteine s-sulfate	0.44	1.60E-05	8.69E-03	1
Amino Acid	Alanine and Aspartate Metabolism	N-acetylasparagine	2.00	4.21E-03	2.12E-01	0
Amino Acid	Glutathione Metabolism	cysteinylglycine	0.58	6.33E-03	2.48E-01	0
Amino Acid	Leucine, Isoleucine and Valine Metabolism	isovalerate (i5:0)	1.76	1.24E-02	2.87E-01	0
Amino Acid	Methionine, Cysteine, SAM and Taurine Metabolism	S-methylcysteine	0.63	1.50E-02	2.97E-01	0
Amino Acid	Tryptophan Metabolism	serotonin	1.58	2.67E-02	3.87E-01	0
Amino Acid	Histidine Metabolism	histidine	0.64	2.77E-02	3.87E-01	0
Amino Acid	Glutathione Metabolism	cys-gly, oxidized	0.67	3.61E-02	4.46E-01	0
Cofactors and Vitamins	Tocopherol Metabolism	alpha-tocopherol	1.96	1.94E-03	1.51E-01	0
Cofactors and Vitamins	Nicotinate and Nicotinamide Metabolism	quinolinate	2.00	8.79E-03	2.66E-01	0
Cofactors and Vitamins	Tocopherol Metabolism	gamma-tocopherol/beta-tocopherol	0.65	2.66E-02	3.87E-01	0
Cofactors and Vitamins	Hemoglobin and Porphyrin Metabolism	biliverdin	1.65	3.05E-02	3.95E-01	0
Lipid	Lysoplasmalogen	1-(1-enyl-palmitoyl)-GPE (P-16:0)*	2.47	2.98E-04	7.51E-02	0
Lipid	Plasmalogen	1-(1-enyl-palmitoyl)-2-linoleoyl-GPE (P-16:0/18:2)*	2.03	6.16E-04	7.51E-02	0
Lipid	Lysoplasmalogen	1-(1-enyl-stearoyl)-GPE (P-18:0)*	2.24	6.67E-04	7.51E-02	0
Lipid	Lysoplasmalogen	1-(1-enyl-oleoyl)-GPE (P-18:1)*	2.01	2.36E-03	1.61E-01	0
Lipid	Plasmalogen	1-(1-enyl-palmitoyl)-2-arachidonoyl-GPE (P-16:0/20:4)*	1.78	3.72E-03	2.12E-01	0
Lipid	Phosphatidylcholine (PC)	1-stearoyl-2-oleoyl-GPC (18:0/18:1)	0.54	4.28E-03	2.12E-01	0
Lipid	Fatty Acid Metabolism(Acyl Carnitine)	myristoylcarnitine (C14)	0.57	6.15E-03	2.48E-01	0
Lipid	Phosphatidylcholine (PC)	1-palmitoyl-2-oleoyl-GPC (16:0/18:1)	0.57	6.37E-03	2.48E-01	0
Lipid	Diacylglycerol	oleoyl-arachidonoyl-glycerol (18:1/20:4) [2]*	0.59	7.13E-03	2.58E-01	0
Lipid	Fatty Acid Metabolism(Acyl Carnitine)	palmitoylcarnitine (C16)	0.57	7.80E-03	2.65E-01	0
Lipid	Lysoplasmalogen	1-(1-enyl-palmitoyl)-GPC (P-16:0)*	1.81	8.69E-03	2.66E-01	0
Lipid	Fatty Acid Metabolism(Acyl Carnitine)	laurylcarnitine (C12)	0.61	1.08E-02	2.87E-01	0
Lipid	Phosphatidylcholine (PC)	1-stearoyl-2-arachidonoyl-GPC (18:0/20:4)	0.56	1.14E-02	2.87E-01	0
Lipid	Phosphatidylcholine (PC)	1-linoleoyl-2-arachidonoyl-GPC (18:2/20:4n6)*	0.58	1.22E-02	2.87E-01	0
Lipid	Fatty Acid Metabolism(Acyl Carnitine)	oleoylcarnitine (C18:1)	0.60	1.27E-02	2.87E-01	0
Lipid	Diacylglycerol	oleoyl-linoleoyl-glycerol (18:1/18:2) [2]	0.62	1.49E-02	2.97E-01	0
Lipid	Fatty Acid Metabolism(Acyl Carnitine)	palmitoleoylcarnitine (C16:1)*	0.62	1.50E-02	2.97E-01	0
Lipid	Fatty Acid Metabolism(Acyl Carnitine)	margaroylcarnitine (C17)*	0.60	1.54E-02	2.97E-01	0
Lipid	Fatty Acid Metabolism(Acyl Carnitine)	myristoleoylcarnitine (C14:1)*	0.63	1.59E-02	2.97E-01	0
Lipid	Phosphatidylinositol (PI)	1-stearoyl-2-arachidonoyl-GPI (18:0/20:4)	0.61	1.67E-02	3.03E-01	0
Lipid	Fatty Acid Metabolism(Acyl Carnitine)	stearoylcarnitine (C18)	0.63	2.29E-02	3.87E-01	0
Lipid	Phospholipid Metabolism	glycerophosphoethanolamine	1.54	2.56E-02	3.87E-01	0
Lipid	Phosphatidylcholine (PC)	1-palmitoyl-2-palmitoleoyl-GPC (16:0/16:1)*	0.65	2.59E-02	3.87E-01	0
Lipid	Fatty Acid Metabolism (Acyl Choline)	linoleoylcholine*	1.54	2.67E-02	3.87E-01	0
Lipid	Phosphatidylcholine (PC)	1-palmitoyl-2-arachidonoyl-GPC (16:0/20:4n6)	0.63	2.74E-02	3.87E-01	0
Lipid	Fatty Acid Metabolism(Acyl Carnitine)	nervonoylcarnitine (C24:1)*	0.64	2.78E-02	3.87E-01	0
Lipid	Diacylglycerol	palmitoyl-linoleoyl-glycerol (16:0/18:2) [2]*	0.66	3.03E-02	3.95E-01	0
Lipid	Diacylglycerol	linoleoyl-arachidonoyl-glycerol (18:2/20:4) [2]*	0.66	3.05E-02	3.95E-01	0
Lipid	Diacylglycerol	oleoyl-oleoyl-glycerol (18:1/18:1) [2]*	0.67	3.51E-02	4.44E-01	0
Lipid	Phosphatidylethanolamine (PE)	1-oleoyl-2-linoleoyl-GPE (18:1/18:2)*	1.48	4.18E-02	4.92E-01	0
Lipid	Diacylglycerol	oleoyl-linoleoyl-glycerol (18:1/18:2) [1]	0.67	4.20E-02	4.92E-01	0
Lipid	Fatty Acid Metabolism(Acyl Carnitine)	ximenoylcarnitine (C26:1)*	0.67	4.29E-02	4.92E-01	0
Lipid	Fatty Acid Metabolism(Acyl Carnitine)	dihomo-linolenoylcarnitine (C20:3n3 or 6)*	0.66	4.43E-02	4.92E-01	0
Lipid	Diacylglycerol	palmitoyl-oleoyl-glycerol (16:0/18:1) [2]*	0.69	4.67E-02	5.09E-01	0
Nucleotide	Purine Metabolism, Adenine containing	adenosine	2.15	6.90E-04	7.51E-02	0
Nucleotide	Pyrimidine Metabolism, Cytidine containing	cytidine	2.12	1.35E-03	1.22E-01	0

Nucleotide	Purine Metabolism, (Hypo)Xanthine/Inosine containing	inosine	1.65	9.69E-03	2.77E-01	0
Nucleotide	Purine Metabolism, Adenine containing	adenine	1.54	4.38E-02	4.92E-01	0

Legend: OR – Odds Ratio; Pval – P-value; Qval – q-value; US – United States; Qval_Sig – indicator for significance based on q-value.

Supplementary Table 2. Metabolites differing significantly between patients with AMD and controls, using Coimbra (Portugal) samples based on p-values of AMD/Control model.

Super Pathway	Sub Pathway	Metabolite	OR PT	Pval PT	Qval PT	Qval Sig
Amino Acid	Methionine, Cysteine, SAM and Taurine Metabolism	taurine	3.70	1.19E-07	9.23E-06	1
Amino Acid	Glutamate Metabolism	beta-citrylglutamate	2.87	1.37E-05	5.74E-04	1
Amino Acid	Methionine, Cysteine, SAM and Taurine Metabolism	N-acetylmethionine	2.55	1.85E-05	7.21E-04	1
Amino Acid	Alanine and Aspartate Metabolism	aspartate	2.37	6.69E-05	2.02E-03	1
Amino Acid	Methionine, Cysteine, SAM and Taurine Metabolism	S-adenosylhomocysteine (SAH)	2.10	3.91E-04	9.25E-03	1
Amino Acid	Methionine, Cysteine, SAM and Taurine Metabolism	hypotaurine	2.32	4.69E-04	1.06E-02	1
Amino Acid	Tryptophan Metabolism	serotonin	2.00	6.20E-04	1.35E-02	1
Amino Acid	Glutamate Metabolism	glutamate	1.82	3.32E-03	6.45E-02	0
Amino Acid	Lysine Metabolism	6-oxopiperidine-2-carboxylate	1.82	4.95E-03	8.98E-02	0
Amino Acid	Glutathione Metabolism	cysteine-glutathione disulfide	1.61	6.85E-03	1.13E-01	0
Amino Acid	Leucine, Isoleucine and Valine Metabolism	isovalerylcarnitine (C5)	1.66	7.72E-03	1.20E-01	0
Amino Acid	Urea cycle; Arginine and Proline Metabolism	homocitrulline	1.79	1.09E-02	1.52E-01	0
Amino Acid	Glutamate Metabolism	glutamine	0.66	1.90E-02	2.47E-01	0
Amino Acid	Alanine and Aspartate Metabolism	N-acetylasparagine	1.55	1.96E-02	2.48E-01	0
Amino Acid	Leucine, Isoleucine and Valine Metabolism	ethylmalonate	1.58	2.52E-02	2.92E-01	0
Amino Acid	Polyamine Metabolism	spermidine	1.53	2.60E-02	2.95E-01	0
Amino Acid	Tryptophan Metabolism	C-glycosyltryptophan	1.74	2.91E-02	3.16E-01	0
Amino Acid	Histidine Metabolism	1-methylhistidine	1.55	3.50E-02	3.66E-01	0
Amino Acid	Urea cycle; Arginine and Proline Metabolism	proline	0.65	3.56E-02	3.66E-01	0
Amino Acid	Lysine Metabolism	N6-acetyllysine	1.65	3.80E-02	3.76E-01	0
Amino Acid	Alanine and Aspartate Metabolism	N-acetylalanine	1.58	3.95E-02	3.77E-01	0
Amino Acid	Leucine, Isoleucine and Valine Metabolism	3-methylglutarylcarnitine (2)	1.48	4.70E-02	4.27E-01	0
Carbohydrate	Glycogen Metabolism	maltotriose	3.79	2.44E-07	1.66E-05	1
Carbohydrate	Glycogen Metabolism	maltose	3.32	1.62E-06	8.02E-05	1
Carbohydrate	Aminosugar Metabolism	N-acetylneuraminate	2.69	8.79E-05	2.52E-03	1
Carbohydrate	Aminosugar Metabolism	glucuronate	1.75	2.43E-02	2.87E-01	0
Cofactors and Vitamins	Nicotinate and Nicotinamide Metabolism	nicotinamide	2.34	1.91E-04	4.71E-03	1
Energy	Oxidative Phosphorylation	phosphate	1.64	7.39E-03	1.18E-01	0
Lipid	Phosphatidylserine (PS)	1-stearoyl-2-oleoyl-GPS (18:0/18:1)	5.74	2.94E-10	1.60E-07	1
Lipid	Sphingosines	sphingosine	16.67	1.89E-09	5.13E-07	1
Lipid	Phospholipid Metabolism	choline phosphate	4.95	4.01E-09	7.27E-07	1
Lipid	Fatty Acid, Monohydroxy	14-HDoHE/17-HDoHE	5.02	8.02E-09	1.09E-06	1
Lipid	Phospholipid Metabolism	phosphoethanolamine	3.97	2.36E-08	2.36E-06	1
Lipid	Eicosanoid	12-HETE	4.77	2.61E-08	2.36E-06	1
Lipid	Sphingolipid Synthesis	sphinganine	5.33	6.18E-07	3.36E-05	1
Lipid	Sphingosines	sphingosine 1-phosphate	2.75	3.35E-06	1.52E-04	1
Lipid	Fatty Acid Metabolism (also BCAA Metabolism)	butyrylcarnitine (C4)	2.18	1.69E-03	3.40E-02	1
Lipid	Fatty Acid Metabolism(Acyl Carnitine)	dihomo-linoleoylcarnitine (C20:2)*	1.71	4.78E-03	8.96E-02	0
Lipid	Lysoplasmalogen	1-(1-enyl-palmitoyl)-GPE (P-16:0)*	1.70	5.86E-03	1.01E-01	0
Lipid	Fatty Acid Metabolism(Acyl Carnitine)	dihomo-linolenoylcarnitine (C20:3n3 or 6)*	1.72	5.96E-03	1.01E-01	0
Lipid	Fatty Acid Metabolism(Acyl Carnitine)	linoleoylcarnitine (C18:2)*	1.65	7.99E-03	1.21E-01	0
Lipid	Fatty Acid Metabolism(Acyl Carnitine)	linolenoylcarnitine (C18:3)*	1.62	9.74E-03	1.43E-01	0
Lipid	Fatty Acid Metabolism(Acyl Carnitine)	acetylcarnitine (C2)	1.65	1.05E-02	1.50E-01	0
Lipid	Fatty Acid, Monohydroxy	9-hydroxystearate	1.59	1.34E-02	1.78E-01	0
Lipid	Fatty Acid Metabolism(Acyl Carnitine)	arachidonoylcarnitine (C20:4)	1.54	2.27E-02	2.74E-01	0
Lipid	Lactosylceramides (LCER)	lactosyl-N-nervonoyl-sphingosine (d18:1/24:1)*	1.50	2.70E-02	3.00E-01	0
Lipid	Secondary Bile Acid Metabolism	deoxycholate	0.70	3.02E-02	3.22E-01	0
Lipid	Sphingomyelins	lignoceroyl sphingomyelin (d18:1/24:0)	1.43	3.79E-02	3.76E-01	0

Lipid	Fatty Acid, Dicarboxylate	3-carboxy-4-methyl-5-propyl-2-furanpropanoate (CMPF)	1.45	3.94E-02	3.77E-01	0
Lipid	Sphingomyelins	behenoyl sphingomyelin (d18:1/22:0)*	1.44	4.18E-02	3.92E-01	0
Lipid	Monoacylglycerol	2-oleoylglycerol (18:1)	0.72	4.67E-02	4.27E-01	0
Lipid	Progestin Steroids	pregnanediol-3-glucuronide	1.43	4.79E-02	4.27E-01	0
Lipid	Secondary Bile Acid Metabolism	glycodeoxycholate	0.71	4.99E-02	4.37E-01	0
Nucleotide	Purine Metabolism, Guanine containing	guanine	3.70	5.99E-07	3.36E-05	1
Nucleotide	Purine Metabolism, (Hypo)Xanthine/Inosine containing	hypoxanthine	2.52	3.58E-05	1.30E-03	1
Nucleotide	Pyrimidine Metabolism, Cytidine containing	cytidine	2.55	6.24E-05	2.02E-03	1
Nucleotide	Purine Metabolism, Adenine containing	adenosine	2.58	6.33E-05	2.02E-03	1
Nucleotide	Purine Metabolism, (Hypo)Xanthine/Inosine containing	inosine	2.29	1.54E-04	4.00E-03	1
Nucleotide	Pyrimidine Metabolism, Orotate containing	dihydroorotate	0.53	7.52E-04	1.57E-02	1
Nucleotide	Pyrimidine Metabolism, Uracil containing	uracil	1.54	1.18E-02	1.60E-01	0
Nucleotide	Purine Metabolism, Adenine containing	adenine	1.63	2.02E-02	2.50E-01	0
Peptide	Dipeptide	isoleucylglycine	2.15	1.25E-04	3.40E-03	1

Legend: OR – Odds Ratio; Pval – P-value; Qval – q-value; PT– Portugal; Qval_Sig – indicator for significance based on q-value.

Supplementary Table 3. Metabolites differing significantly (p-value) between patients with AMD and controls, based on meta-analysis of AMD/Control model from the 2 study cohorts.

Super Pathway	Sub Pathway	Metabolite	OR PT	Pval PT	OR US	Pval US	OR Meta	Pval Meta	Qval Meta	Qval Sig
Amino Acid	Methionine, Cysteine, SAM and Taurine Metabolism	taurine	3.70	1.19E-07	1.26	2.46E-01	2.04	9.59E-07	6.52E-05	1
Amino Acid	Glutamate Metabolism	beta-citrylglutamate	2.87	1.37E-05	1.52	5.40E-02	2.06	3.86E-06	2.05E-04	1
Amino Acid	Tryptophan Metabolism	serotonin	2.00	6.20E-04	1.58	2.67E-02	1.82	4.84E-05	1.65E-03	1
Amino Acid	Methionine, Cysteine, SAM and Taurine Metabolism	N-acetylmethionine	2.55	1.85E-05	1.20	4.05E-01	1.75	9.74E-05	2.92E-03	1
Amino Acid	Alanine and Aspartate Metabolism	aspartate	2.37	6.69E-05	1.28	2.32E-01	1.80	1.02E-04	2.92E-03	1
Amino Acid	Methionine, Cysteine, SAM and Taurine Metabolism	hypotaurine	2.32	4.69E-04	1.36	1.47E-01	1.85	2.57E-04	6.99E-03	1
Amino Acid	Alanine and Aspartate Metabolism	N-acetylasparagine	1.55	1.96E-02	2.00	4.21E-03	1.69	3.22E-04	7.96E-03	1
Amino Acid	Methionine, Cysteine, SAM and Taurine Metabolism	S-adenosylhomocysteine (SAH)	2.10	3.91E-04	1.24	3.21E-01	1.67	6.43E-04	1.52E-02	1
Amino Acid	Leucine, Isoleucine and Valine Metabolism	isovalerate (i5:0)	1.33	1.12E-01	1.76	1.24E-02	1.49	5.30E-03	9.40E-02	0
Amino Acid	Lysine Metabolism	6-oxopiperidine-2-carboxylate	1.82	4.95E-03	1.12	5.59E-01	1.38	9.86E-03	1.58E-01	0
Amino Acid	Lysine Metabolism	N6-acetyllysine	1.65	3.80E-02	1.29	2.18E-01	1.47	1.66E-02	2.20E-01	0
Amino Acid	Leucine, Isoleucine and Valine Metabolism	isovalerylcarnitine (C5)	1.66	7.72E-03	1.07	7.10E-01	1.31	1.95E-02	2.42E-01	0
Amino Acid	Histidine Metabolism	histidine	0.80	2.15E-01	0.64	2.77E-02	0.73	2.00E-02	2.42E-01	0
Amino Acid	Glutamate Metabolism	glutamine	0.66	1.90E-02	0.87	4.81E-01	0.75	2.23E-02	2.45E-01	0
Amino Acid	Polyamine Metabolism	4-acetamidobutanoate	1.41	7.49E-02	1.34	1.67E-01	1.37	2.42E-02	2.53E-01	0
Amino Acid	Glutathione Metabolism	cysteinylglycine	0.90	5.38E-01	0.58	6.33E-03	0.74	3.10E-02	2.98E-01	0
Amino Acid	Histidine Metabolism	1-methylhistidine	1.55	3.50E-02	1.19	4.33E-01	1.40	3.17E-02	2.98E-01	0
Amino Acid	Glutamate Metabolism	glutamate	1.82	3.32E-03	0.94	7.74E-01	1.30	3.17E-02	2.98E-01	0
Amino Acid	Leucine, Isoleucine and Valine Metabolism	3-methylglutarylcarnitine (2)	1.48	4.70E-02	1.20	4.06E-01	1.34	3.76E-02	3.41E-01	0
Amino Acid	Glutathione Metabolism	cysteine-glutathione disulfide	1.61	6.85E-03	0.98	9.11E-01	1.29	3.83E-02	3.42E-01	0
Amino Acid	Urea cycle; Arginine and Proline Metabolism	proline	0.65	3.56E-02	0.89	5.56E-01	0.76	4.31E-02	3.61E-01	0
Amino Acid	Glutathione Metabolism	cys-gly, oxidized	0.86	3.91E-01	0.67	3.61E-02	0.77	5.00E-02	3.91E-01	0
Carbohydrate	Glycogen Metabolism	maltotriose	3.79	2.44E-07	1.49	5.12E-02	2.47	1.31E-07	1.75E-05	1
Carbohydrate	Glycogen Metabolism	maltose	3.32	1.62E-06	1.29	1.86E-01	2.14	4.14E-06	2.05E-04	1
Carbohydrate	Aminosugar Metabolism	N-acetylneuraminate	2.69	8.79E-05	0.89	5.60E-01	1.44	6.01E-03	1.02E-01	0
Carbohydrate	Glycolysis, Gluconeogenesis, and Pyruvate Metabolism	glucose	0.75	1.09E-01	0.72	9.76E-02	0.74	2.25E-02	2.45E-01	0
Cofactors and Vitamins	Nicotinate and Nicotinamide Metabolism	nicotinamide	2.34	1.91E-04	1.15	5.08E-01	1.68	7.87E-04	1.78E-02	1
Cofactors and Vitamins	Nicotinate and Nicotinamide Metabolism	quinolinate	1.10	6.35E-01	2.00	8.79E-03	1.33	4.79E-02	3.83E-01	0
Energy	TCA Cycle	succinylcarnitine (C4-DC)	1.32	1.21E-01	1.50	5.91E-02	1.36	1.72E-02	2.23E-01	0
Energy	Oxidative Phosphorylation	phosphate	1.64	7.39E-03	0.96	8.47E-01	1.31	4.53E-02	3.73E-01	0
Lipid	Phosphatidylserine (PS)	1-stearoyl-2-oleoyl-GPS (18:0/18:1)	5.74	2.94E-10	1.23	2.92E-01	2.35	1.80E-08	6.64E-06	1
Lipid	Phospholipid Metabolism	phosphoethanolamine	3.97	2.36E-08	1.44	5.79E-02	2.49	2.44E-08	6.64E-06	1
Lipid	Phospholipid Metabolism	choline phosphate	4.95	4.01E-09	1.21	3.21E-01	2.38	1.42E-07	1.75E-05	1
Lipid	Sphingosines	sphingosine	16.67	1.89E-09	0.99	9.68E-01	1.75	2.26E-06	1.36E-04	1
Lipid	Lysoplasmalogen	1-(1-enyl-palmitoyl)-GPE (P-16:0)*	1.70	5.86E-03	2.47	2.98E-04	1.97	1.12E-05	4.69E-04	1
Lipid	Fatty Acid, Monohydroxy	14-HDoHE/17-HDoHE	5.02	8.02E-09	0.87	4.63E-01	1.73	3.87E-05	1.46E-03	1
Lipid	Eicosanoid	12-HETE	4.77	2.61E-08	0.91	6.26E-01	1.64	4.03E-05	1.46E-03	1
Lipid	Sphingolipid Synthesis	sphinganine	5.33	6.18E-07	0.90	5.85E-01	1.44	3.05E-04	7.91E-03	1
Lipid	Lysoplasmalogen	1-(1-enyl-oleoyl)-GPE (P-18:1)*	1.35	9.20E-02	2.01	2.36E-03	1.60	1.41E-03	2.95E-02	1
Lipid	Lysoplasmalogen	1-(1-enyl-stearoyl)-GPE (P-18:0)*	1.25	1.89E-01	2.24	6.67E-04	1.58	1.81E-03	3.65E-02	1
Lipid	Sphingosines	sphingosine 1-phosphate	2.75	3.35E-06	0.77	1.86E-01	1.46	4.12E-03	7.74E-02	0
Lipid	Fatty Acid Metabolism (also BCAA Metabolism)	butyrylcarnitine (C4)	2.18	1.69E-03	1.06	7.94E-01	1.40	8.18E-03	1.35E-01	0
Lipid	Diacylglycerol	oleoyl-oleoyl-glycerol (18:1/18:1) [2]*	0.79	1.37E-01	0.67	3.51E-02	0.74	1.37E-02	2.11E-01	0
Lipid	Fatty Acid, Dicarboxylate	3-carboxy-4-methyl-5-propyl-2-furanpropanoate (CMPF)	1.45	3.94E-02	1.32	1.76E-01	1.36	1.40E-02	2.11E-01	0
Lipid	Secondary Bile Acid Metabolism	deoxycholate	0.70	3.02E-02	0.80	2.45E-01	0.74	1.53E-02	2.20E-01	0
Lipid	Diacylglycerol	oleoyl-arachidonoyl-glycerol (18:1/20:4) [2]*	0.86	3.34E-01	0.59	7.13E-03	0.74	1.59E-02	2.20E-01	0
Lipid	Diacylglycerol	palmitoyl-linoleoyl-glycerol (16:0/18:2) [2]*	0.80	1.72E-01	0.66	3.03E-02	0.74	1.61E-02	2.20E-01	0
Lipid	Phosphatidylcholine (PC)	1-stearoyl-2-oleoyl-GPC (18:0/18:1)	0.88	4.09E-01	0.54	4.28E-03	0.73	1.64E-02	2.20E-01	0

Lipid	Diacylglycerol	oleoyl-oleoyl-glycerol (18:1/18:1) [1]*	0.78	1.22E-01	0.70	6.11E-02	0.75	1.79E-02	2.26E-01	0
Lipid	Phosphatidylcholine (PC)	1-palmitoyl-2-oleoyl-GPC (16:0/18:1)	0.88	4.18E-01	0.57	6.37E-03	0.74	2.09E-02	2.45E-01	0
Lipid	Diacylglycerol	oleoyl-linoleoyl-glycerol (18:1/18:2) [1]	0.80	1.91E-01	0.67	4.20E-02	0.74	2.27E-02	2.45E-01	0
Lipid	Diacylglycerol	oleoyl-linoleoyl-glycerol (18:1/18:2) [2]	0.84	3.19E-01	0.62	1.49E-02	0.73	2.28E-02	2.45E-01	0
Lipid	Diacylglycerol	palmitoyl-oleoyl-glycerol (16:0/18:1) [2]*	0.82	1.81E-01	0.69	4.67E-02	0.76	2.29E-02	2.45E-01	0
Lipid	Fatty Acid Metabolism(Acyl Carnitine)	cerotoylcarnitine (C26)*	0.75	1.01E-01	0.74	1.22E-01	0.75	2.50E-02	2.56E-01	0
Lipid	Diacylglycerol	oleoyl-arachidonoyl-glycerol (18:1/20:4) [1]*	0.81	1.67E-01	0.71	7.18E-02	0.76	2.82E-02	2.84E-01	0
Lipid	Plasmalogen	1-(1-enyl-palmitoyl)-2-arachidonoyl-GPE (P-16:0/20:4)*	1.09	6.11E-01	1.78	3.72E-03	1.31	2.95E-02	2.92E-01	0
Lipid	Sphingomyelins	sphingomyelin (d18:1/20:1, d18:2/20:0)*	1.24	2.38E-01	1.52	5.18E-02	1.35	3.38E-02	3.12E-01	0
Lipid	Diacylglycerol	palmitoyl-linoleoyl-glycerol (16:0/18:2) [1]*	0.81	2.07E-01	0.72	8.58E-02	0.77	4.04E-02	3.54E-01	0
Lipid	Phosphatidylcholine (PC)	1-palmitoyl-2-arachidonoyl-GPC (16:0/20:4n6)	0.87	3.87E-01	0.63	2.74E-02	0.76	4.19E-02	3.61E-01	0
Lipid	Phosphatidylcholine (PC)	1-stearoyl-2-arachidonoyl-GPC (18:0/20:4)	0.90	5.49E-01	0.56	1.14E-02	0.74	4.32E-02	3.61E-01	0
Lipid	Sphingomyelins	sphingomyelin (d18:2/24:2)*	1.32	1.46E-01	1.31	1.70E-01	1.32	4.65E-02	3.78E-01	0
Nucleotide	Purine Metabolism, Adenine containing	adenosine	2.58	6.33E-05	2.15	6.90E-04	2.39	1.60E-07	1.75E-05	1
Nucleotide	Pyrimidine Metabolism, Cytidine containing	cytidine	2.55	6.24E-05	2.12	1.35E-03	2.37	2.93E-07	2.66E-05	1
Nucleotide	Purine Metabolism, Guanine containing	guanine	3.70	5.99E-07	1.39	1.08E-01	2.40	8.16E-07	6.34E-05	1
Nucleotide	Purine Metabolism, (Hypo)Xanthine/Inosine containing	inosine	2.29	1.54E-04	1.65	9.69E-03	2.02	4.74E-06	2.15E-04	1
Nucleotide	Purine Metabolism, (Hypo)Xanthine/Inosine containing	hypoxanthine	2.52	3.58E-05	1.24	2.79E-01	1.80	8.43E-05	2.70E-03	1
Nucleotide	Purine Metabolism, Adenine containing	adenine	1.63	2.02E-02	1.54	4.38E-02	1.58	2.13E-03	4.14E-02	1
Nucleotide	Pyrimidine Metabolism, Orotate containing	dihydroorotate	0.53	7.52E-04	0.96	8.46E-01	0.70	5.35E-03	9.40E-02	0
Peptide	Dipeptide	isoleucylglycine	2.15	1.25E-04	1.07	7.26E-01	1.58	1.15E-03	2.51E-02	1

Legend: OR – Odds Ratio; Pval – P-value; Qval – q-value; PT– Portugal; US – United States; Meta – Meta analysis; Qval_Sig - indicator for significance based on q-value.

Supplementary Table 4. Characterization of the included study population, by eye.

Boston, US

	**Control**	**Early AMD**	**Intermediate AMD**	**Late AMD**	**p-value**
**Number of patients**, n (%)	105 (27)	63 (16)	142 (36)	82 (21)	NA
**Age**, mean ± SD	67.9 ± 8.2	69.3 ± 7.9	72.5 ± 7.2	76.0 ± 8.2	<0.001*
**BMI**, mean ± SD	27.2 ± 5.1	26.6 ± 4.5	27.2 ± 5.0	27.1 ± 4.5	0.887
**Gender** n, (%)					
Female	64 (61)	43 (68)	94 (66)	51 (62)	0.724
Male	41 (39)	20 (32)	48 (34)	31 (38)	
**Smoking** n, (%)					
Non-smoker	54 (52)	37 (59)	60 (42)	27 (35)	0.009*
Ex-smoker	45 (44)	26 (41)	76 (54)	51 (65)	
Current smoker	4 (4)	0 (0)	6 (4)	0 (0)	
**Race** n, (%)					
White	87 (95)	55 (92)	137 (97)	73 (95)	0.192
Black	2 (2)	0 (0)	0 (0)	0 (0)	
Asian	1 (1)	1 (2)	2 (1)	0 (0)	
Hispanic	2 (2)	4 (7)	2 (1)	4 (5)	

Coimbra, Portugal

	**Control**	**Early AMD**	**Intermediate AMD**	**Late AMD**	**p-value**
**Number of patients**, n (%)	138	124	249	79	NA
**Age**, mean ± SD	69.8 ± 6.2	72.4 ± 6.6	76.9 ± 7.5	82.2 ± 6.8	<0.0001*
**BMI**, mean ± SD	26.9 ± 4.4	27.4 ± 4.6	27.3 ± 4.4	26.6 ± 4.2	0.477
**Gender** n, (%)					
Female	87 (63)	76 (61)	170 (68)	51 (65)	0.539
Male	51 (37)	48 (39)	79 (32)	28 (35)	
**Smoking** n, (%)					
Non-smoker	115 (83)	105 (85)	215 (86)	61 (77)	0.342
Ex-smoker	23 (17)	19 (15)	33 (13)	17 (22)	
Current smoker	0 (0)	0 (0)	1 (0)	1 (1)	
**Race** n, (%)					
White	138 (100)	124 (100)	245 (98)	77 (97)	0.127
Black	0 (0)	0 (0)	4 (2)	2 (3)	
Asian	0 (0)	0 (0)	0 (0)	0 (0)	
Hispanic	0 (0)	0 (0)	0 (0)	0 (0)	

Supplementary Table 5. Metabolites differing significantly across all study groups using Boston (US) samples based on p-values from Stage+2Eye model.

Super Pathway	Sub Pathway	Metabolite	OR US	Pval US	Qval US	Qval Sig
Amino Acid	Methionine, Cysteine, SAM and Taurine Metabolism	cysteine s-sulfate	0.53	1.00E-06	2.72E-05	1
Amino Acid	Alanine and Aspartate Metabolism	N-acetylasparagine	1.74	1.00E-06	2.72E-05	1
Amino Acid	Leucine, Isoleucine and Valine Metabolism	isovalerate (i5:0)	1.75	1.00E-06	2.72E-05	1
Amino Acid	Histidine Metabolism	histidine	0.52	1.00E-06	2.72E-05	1
Amino Acid	Methionine, Cysteine, SAM and Taurine Metabolism	S-methylcysteine	0.64	2.00E-05	4.03E-04	1
Amino Acid	Leucine, Isoleucine and Valine Metabolism	2-hydroxy-3-methylvalerate	1.50	1.00E-04	1.55E-03	1
Amino Acid	Glutathione Metabolism	cysteinylglycine	0.69	3.40E-04	3.59E-03	1
Amino Acid	Glutamate Metabolism	beta-citrylglutamate	1.46	3.50E-04	3.59E-03	1
Amino Acid	Leucine, Isoleucine and Valine Metabolism	N-acetylleucine	1.43	7.30E-04	6.30E-03	1
Amino Acid	Methionine, Cysteine, SAM and Taurine Metabolism	S-methylcysteine sulfoxide	0.70	7.90E-04	6.72E-03	1
Amino Acid	Leucine, Isoleucine and Valine Metabolism	alpha-hydroxyisovalerate	1.40	1.37E-03	1.10E-02	1
Amino Acid	Urea cycle; Arginine and Proline Metabolism	N-acetylarginine	1.40	1.40E-03	1.10E-02	1
Amino Acid	Tyrosine Metabolism	p-cresol glucuronide*	1.40	1.62E-03	1.21E-02	1
Amino Acid	Leucine, Isoleucine and Valine Metabolism	isobutyrylglycine	1.40	1.67E-03	1.23E-02	1
Amino Acid	Leucine, Isoleucine and Valine Metabolism	isovalerylglycine	1.39	1.72E-03	1.25E-02	1
Amino Acid	Urea cycle; Arginine and Proline Metabolism	N-acetylcitrulline	1.36	3.83E-03	2.32E-02	1
Amino Acid	Urea cycle; Arginine and Proline Metabolism	N2,N5-diacetylornithine	1.36	5.19E-03	2.94E-02	1
Amino Acid	Tryptophan Metabolism	serotonin	1.32	5.54E-03	3.08E-02	1
Amino Acid	Glutamate Metabolism	glutamine	0.74	6.07E-03	3.30E-02	1
Amino Acid	Glutathione Metabolism	cys-gly, oxidized	0.75	6.55E-03	3.53E-02	1
Amino Acid	Tyrosine Metabolism	N-acetyltyrosine	1.32	8.54E-03	4.19E-02	1
Amino Acid	Lysine Metabolism	N2-acetyllysine	1.31	9.89E-03	4.72E-02	1
Amino Acid	Tryptophan Metabolism	kynurenate	1.33	1.21E-02	5.51E-02	0
Amino Acid	Urea cycle; Arginine and Proline Metabolism	ornithine	0.78	1.45E-02	6.43E-02	0
Amino Acid	Alanine and Aspartate Metabolism	N-acetylaspartate (NAA)	1.33	1.52E-02	6.56E-02	0
Amino Acid	Tyrosine Metabolism	3-(4-hydroxyphenyl)lactate	1.31	1.58E-02	6.72E-02	0
Amino Acid	Methionine, Cysteine, SAM and Taurine Metabolism	N-acetyltaurine	1.31	1.70E-02	7.11E-02	0
Amino Acid	Phenylalanine Metabolism	phenyllactate (PLA)	1.29	2.12E-02	8.34E-02	0
Amino Acid	Glycine, Serine and Threonine Metabolism	dimethylglycine	0.77	2.13E-02	8.35E-02	0
Amino Acid	Histidine Metabolism	3-methylhistidine	1.27	2.24E-02	8.63E-02	0
Amino Acid	Tryptophan Metabolism	indole-3-carboxylic acid	1.27	2.38E-02	9.05E-02	0
Amino Acid	Leucine, Isoleucine and Valine Metabolism	N-acetylvaline	1.29	2.50E-02	9.43E-02	0
Amino Acid	Methionine, Cysteine, SAM and Taurine Metabolism	S-methylmethionine	0.79	2.68E-02	9.99E-02	0
Amino Acid	Histidine Metabolism	formiminoglutamate	1.30	2.71E-02	1.00E-01	0
Amino Acid	Methionine, Cysteine, SAM and Taurine Metabolism	methionine sulfoxide	1.25	2.79E-02	1.03E-01	0
Amino Acid	Leucine, Isoleucine and Valine Metabolism	N-acetylisoleucine	1.30	2.83E-02	1.03E-01	0
Amino Acid	Glycine, Serine and Threonine Metabolism	sarcosine	1.25	2.84E-02	1.03E-01	0
Amino Acid	Urea cycle; Arginine and Proline Metabolism	2-oxoarginine*	1.25	2.87E-02	1.03E-01	0
Amino Acid	Leucine, Isoleucine and Valine Metabolism	3-hydroxy-2-ethylpropionate	1.27	2.92E-02	1.04E-01	0
Amino Acid	Histidine Metabolism	N-acetyl-1-methylhistidine*	1.26	3.98E-02	1.35E-01	0
Amino Acid	Glutamate Metabolism	glutamate	0.81	4.08E-02	1.37E-01	0
Amino Acid	Glutathione Metabolism	cysteine-glutathione disulfide	0.82	4.42E-02	1.45E-01	0
Amino Acid	Methionine, Cysteine, SAM and Taurine Metabolism	cysteine sulfinic acid	1.22	4.92E-02	1.59E-01	0
Carbohydrate	Aminosugar Metabolism	N-acetylglucosamine/N-acetylgalactosamine	1.52	2.20E-04	2.60E-03	1
Carbohydrate	Aminosugar Metabolism	glucuronate	0.80	3.75E-02	1.30E-01	0
Carbohydrate	Glycolysis, Gluconeogenesis, and Pyruvate Metabolism	glucose	0.80	4.12E-02	1.37E-01	0
Cofactors and Vitamins	Tocopherol Metabolism	alpha-tocopherol	2.00	1.00E-06	2.72E-05	1
Cofactors and Vitamins	Hemoglobin and Porphyrin Metabolism	biliverdin	1.52	1.60E-04	2.12E-03	1

Cofactors and Vitamins	Tocopherol Metabolism	gamma-tocopherol/beta-tocopherol	0.74	4.12E-03	2.46E-02	1
Cofactors and Vitamins	Nicotinate and Nicotinamide Metabolism	quinolinate	1.31	2.03E-02	8.18E-02	0
Energy	TCA Cycle	2-methylcitrate/homocitrate	1.42	2.10E-03	1.43E-02	1
Energy	TCA Cycle	malate	1.25	3.58E-02	1.26E-01	0
Lipid	Sphingomyelins	stearoyl sphingomyelin (d18:1/18:0)	1.74	1.00E-06	2.72E-05	1
Lipid	Fatty Acid Metabolism(Acyl Carnitine)	laurylcarnitine (C12)	0.61	1.00E-06	2.72E-05	1
Lipid	Fatty Acid Metabolism(Acyl Carnitine)	oleoylcarnitine (C18:1)	0.60	1.00E-06	2.72E-05	1
Lipid	Lysoplasmalogen	1-(1-enyl-palmitoyl)-GPE (P-16:0)*	1.95	1.00E-06	2.72E-05	1
Lipid	Lysoplasmalogen	1-(1-enyl-stearoyl)-GPE (P-18:0)*	1.95	1.00E-06	2.72E-05	1
Lipid	Lysoplasmalogen	1-(1-enyl-oleoyl)-GPE (P-18:1)*	1.75	1.00E-06	2.72E-05	1
Lipid	Fatty Acid Metabolism(Acyl Carnitine)	palmitoylcarnitine (C16)	0.63	1.00E-06	2.72E-05	1
Lipid	Fatty Acid Metabolism(Acyl Carnitine)	myristoleoylcarnitine (C14:1)*	0.62	1.00E-06	2.72E-05	1
Lipid	Phosphatidylcholine (PC)	1-stearoyl-2-oleoyl-GPC (18:0/18:1)	0.53	1.00E-06	2.72E-05	1
Lipid	Phosphatidylinositol (PI)	1-stearoyl-2-arachidonoyl-GPI (18:0/20:4)	0.61	1.00E-06	2.72E-05	1
Lipid	Lysoplasmalogen	1-(1-enyl-palmitoyl)-GPC (P-16:0)*	1.80	1.00E-06	2.72E-05	1
Lipid	Fatty Acid Metabolism(Acyl Carnitine)	palmitoleoylcarnitine (C16:1)*	0.62	1.00E-06	2.72E-05	1
Lipid	Diacylglycerol	oleoyl-oleoyl-glycerol (18:1/18:1) [2]*	0.62	1.00E-06	2.72E-05	1
Lipid	Diacylglycerol	oleoyl-arachidonoyl-glycerol (18:1/20:4) [2]*	0.63	1.00E-06	2.72E-05	1
Lipid	Diacylglycerol	oleoyl-linoleoyl-glycerol (18:1/18:2) [1]	0.64	1.00E-05	2.47E-04	1
Lipid	Plasmalogen	1-(1-enyl-palmitoyl)-2-linoleoyl-GPE (P-16:0/18:2)*	1.62	1.00E-05	2.47E-04	1
Lipid	Diacylglycerol	oleoyl-linoleoyl-glycerol (18:1/18:2) [2]	0.63	2.00E-05	4.03E-04	1
Lipid	Phosphatidylcholine (PC)	1-stearoyl-2-linoleoyl-GPC (18:0/18:2)*	0.63	2.00E-05	4.03E-04	1
Lipid	Phosphatidylcholine (PC)	1-palmitoyl-2-oleoyl-GPC (16:0/18:1)	0.61	2.00E-05	4.03E-04	1
Lipid	Fatty Acid Metabolism(Acyl Carnitine)	dihomo-linolenoylcarnitine (C20:3n3 or 6)*	0.65	2.00E-05	4.03E-04	1
Lipid	Sphingomyelins	sphingomyelin (d18:1/20:1, d18:2/20:0)*	1.56	4.00E-05	7.77E-04	1
Lipid	Fatty Acid Metabolism(Acyl Carnitine)	myristoylcarnitine (C14)	0.63	5.00E-05	9.38E-04	1
Lipid	Phosphatidylcholine (PC)	1-linoleoyl-2-arachidonoyl-GPC (18:2/20:4n6)*	0.65	6.00E-05	1.09E-03	1
Lipid	Phosphatidylcholine (PC)	1-stearoyl-2-arachidonoyl-GPC (18:0/20:4)	0.65	7.00E-05	1.19E-03	1
Lipid	Sphingomyelins	palmitoyl sphingomyelin (d18:1/16:0)	1.50	8.00E-05	1.32E-03	1
Lipid	Plasmalogen	1-(1-enyl-palmitoyl)-2-arachidonoyl-GPE (P-16:0/20:4)*	1.50	9.00E-05	1.44E-03	1
Lipid	Phosphatidylcholine (PC)	1-palmitoyl-2-linoleoyl-GPC (16:0/18:2)	0.67	1.10E-04	1.57E-03	1
Lipid	Fatty Acid Metabolism(Acyl Carnitine)	ximenoylcarnitine (C26:1)*	0.67	1.10E-04	1.57E-03	1
Lipid	Fatty Acid Metabolism(Acyl Carnitine)	arachidonoylcarnitine (C20:4)	0.66	1.10E-04	1.57E-03	1
Lipid	Diacylglycerol	linoleoyl-arachidonoyl-glycerol (18:2/20:4) [2]*	0.68	1.20E-04	1.67E-03	1
Lipid	Diacylglycerol	palmitoyl-linoleoyl-glycerol (16:0/18:2) [2]*	0.67	1.60E-04	2.12E-03	1
Lipid	Phosphatidylcholine (PC)	1-palmitoyl-2-stearoyl-GPC (16:0/18:0)	0.66	1.70E-04	2.20E-03	1
Lipid	Fatty Acid Metabolism(Acyl Carnitine)	linolenoylcarnitine (C18:3)*	0.68	1.80E-04	2.28E-03	1
Lipid	Fatty Acid Metabolism(Acyl Carnitine)	decanoylcarnitine (C10)	0.69	2.20E-04	2.60E-03	1
Lipid	Phosphatidylinositol (PI)	1-stearoyl-2-linoleoyl-GPI (18:0/18:2)	0.66	2.50E-04	2.89E-03	1
Lipid	Lysophospholipid	1-palmitoyl-GPG (16:0)*	1.45	2.60E-04	2.95E-03	1
Lipid	Phosphatidylinositol (PI)	1-palmitoyl-2-oleoyl-GPI (16:0/18:1)*	0.68	3.30E-04	3.59E-03	1
Lipid	Fatty Acid Metabolism(Acyl Carnitine)	stearoylcarnitine (C18)	0.69	3.40E-04	3.59E-03	1
Lipid	Sphingomyelins	sphingomyelin (d18:1/22:2, d18:2/22:1, d16:1/24:2)*	1.46	3.50E-04	3.59E-03	1
Lipid	Diacylglycerol	palmitoyl-oleoyl-glycerol (16:0/18:1) [2]*	0.69	3.90E-04	3.86E-03	1
Lipid	Sphingosines	sphingosine 1-phosphate	0.70	4.80E-04	4.66E-03	1
Lipid	Lysophospholipid	1-linoleoyl-GPG (18:2)*	1.44	5.70E-04	5.44E-03	1
Lipid	Fatty Acid Metabolism(Acyl Carnitine)	octanoylcarnitine (C8)	0.71	6.10E-04	5.72E-03	1
Lipid	Diacylglycerol	oleoyl-oleoyl-glycerol (18:1/18:1) [1]*	0.70	6.50E-04	5.99E-03	1
Lipid	Sterol	4-cholesten-3-one	1.44	6.80E-04	6.17E-03	1
Lipid	Diacylglycerol	stearoyl-arachidonoyl-glycerol (18:0/20:4) [1]*	0.70	7.10E-04	6.30E-03	1
Lipid	Fatty Acid Metabolism(Acyl Carnitine)	docosahexaenoylcarnitine (C22:6)*	0.70	7.20E-04	6.30E-03	1

Lipid	Fatty Acid Metabolism(Acyl Carnitine)	linoleoylcarnitine (C18:2)*	0.71	1.12E-03	9.23E-03	1
Lipid	Fatty Acid Metabolism(Acyl Carnitine)	nervonoylcarnitine (C24:1)*	0.71	1.12E-03	9.23E-03	1
Lipid	Fatty Acid Metabolism(Acyl Carnitine)	eicosenoylcarnitine (C20:1)*	0.70	1.24E-03	1.01E-02	1
Lipid	Sphingomyelins	sphingomyelin (d18:2/24:2)*	1.39	1.44E-03	1.12E-02	1
Lipid	Diacylglycerol	palmitoleoyl-linoleoyl-glycerol (16:1/18:2) [1]*	0.71	1.54E-03	1.18E-02	1
Lipid	Fatty Acid Metabolism(Acyl Carnitine)	dihomo-linoleoylcarnitine (C20:2)*	0.71	1.56E-03	1.18E-02	1
Lipid	Fatty Acid Metabolism(Acyl Carnitine)	cerotoylcarnitine (C26)*	0.73	1.79E-03	1.26E-02	1
Lipid	Lysophospholipid	1-lignoceroyl-GPC (24:0)	0.73	1.82E-03	1.27E-02	1
Lipid	Fatty Acid, Monohydroxy	2-hydroxypalmitate	1.38	2.06E-03	1.42E-02	1
Lipid	Phosphatidylcholine (PC)	1-palmitoyl-2-palmitoleoyl-GPC (16:0/16:1)*	0.72	2.17E-03	1.46E-02	1
Lipid	Diacylglycerol	palmitoyl-linoleoyl-glycerol (16:0/18:2) [1]*	0.73	2.21E-03	1.47E-02	1
Lipid	Diacylglycerol	oleoyl-arachidonoyl-glycerol (18:1/20:4) [1]*	0.73	2.29E-03	1.50E-02	1
Lipid	Polyunsaturated Fatty Acid (n3 and n6)	arachidonate (20:4n6)	1.37	2.46E-03	1.59E-02	1
Lipid	Phosphatidylcholine (PC)	1-palmitoyl-2-arachidonoyl-GPC (16:0/20:4n6)	0.73	2.55E-03	1.61E-02	1
Lipid	Phosphatidylinositol (PI)	1-palmitoyl-2-arachidonoyl-GPI (16:0/20:4)*	0.71	2.55E-03	1.61E-02	1
Lipid	Lysophospholipid	1-palmitoyl-GPI (16:0)	1.37	2.65E-03	1.66E-02	1
Lipid	Lysophospholipid	1-linoleoyl-GPI (18:2)*	1.37	2.78E-03	1.72E-02	1
Lipid	Dihydrosphingomyelins	palmitoyl dihydrosphingomyelin (d18:0/16:0)*	1.36	3.39E-03	2.07E-02	1
Lipid	Phosphatidylcholine (PC)	1-linoleoyl-2-linolenoyl-GPC (18:2/18:3)*	0.75	4.82E-03	2.85E-02	1
Lipid	Long Chain Fatty Acid	erucate (22:1n9)	1.35	5.05E-03	2.93E-02	1
Lipid	Lysophospholipid	1-arachidonoyl-GPI (20:4)*	1.35	5.06E-03	2.93E-02	1
Lipid	Androgenic Steroids	5alpha-androstan-3beta,17beta-diol disulfate	1.40	5.44E-03	3.05E-02	1
Lipid	Fatty Acid Metabolism(Acyl Carnitine)	cis-4-decenoylcarnitine (C10:1)	0.76	5.78E-03	3.18E-02	1
Lipid	Phosphatidylcholine (PC)	1,2-dipalmitoyl-GPC (16:0/16:0)	0.75	6.78E-03	3.56E-02	1
Lipid	Lysophospholipid	1-stearoyl-GPI (18:0)	1.32	6.80E-03	3.56E-02	1
Lipid	Fatty Acid Metabolism(Acyl Carnitine)	docosapentaenoylcarnitine (C22:5n3)*	0.76	7.26E-03	3.76E-02	1
Lipid	Fatty Acid Metabolism(Acyl Carnitine)	margaroylcarnitine (C17)*	0.75	7.59E-03	3.79E-02	1
Lipid	Diacylglycerol	linoleoyl-arachidonoyl-glycerol (18:2/20:4) [1]*	0.76	7.98E-03	3.95E-02	1
Lipid	Diacylglycerol	linoleoyl-docosahexaenoyl-glycerol (18:2/22:6) [2]*	0.75	9.49E-03	4.60E-02	1
Lipid	Phosphatidylcholine (PC)	1,2-dilinoleoyl-GPC (18:2/18:2)	0.77	9.55E-03	4.60E-02	1
Lipid	Polyunsaturated Fatty Acid (n3 and n6)	adrenate (22:4n6)	1.31	1.08E-02	5.11E-02	0
Lipid	Fatty Acid, Monohydroxy	16-hydroxypalmitate	1.31	1.09E-02	5.11E-02	0
Lipid	Secondary Bile Acid Metabolism	taurolithocholate 3-sulfate	1.30	1.19E-02	5.50E-02	0
Lipid	Diacylglycerol	linoleoyl-linoleoyl-glycerol (18:2/18:2) [1]*	0.76	1.19E-02	5.50E-02	0
Lipid	Fatty Acid, Dicarboxylate	tetradecanedioate (C14-DC)	1.32	1.29E-02	5.85E-02	0
Lipid	Phosphatidylethanolamine (PE)	1-palmitoyl-2-oleoyl-GPE (16:0/18:1)	0.77	1.36E-02	6.12E-02	0
Lipid	Polyunsaturated Fatty Acid (n3 and n6)	linoleate (18:2n6)	1.30	1.39E-02	6.21E-02	0
Lipid	Polyunsaturated Fatty Acid (n3 and n6)	dihomo-linoleate (20:2n6)	1.29	1.49E-02	6.46E-02	0
Lipid	Polyunsaturated Fatty Acid (n3 and n6)	docosapentaenoate (n3 DPA; 22:5n3)	1.30	1.57E-02	6.72E-02	0
Lipid	Fatty Acid, Dicarboxylate	3-methyladipate	1.30	1.68E-02	7.06E-02	0
Lipid	Phosphatidylinositol (PI)	1-palmitoyl-2-linoleoyl-GPI (16:0/18:2)	0.77	1.71E-02	7.11E-02	0
Lipid	Fatty Acid, Dicarboxylate	hexadecanedioate (C16-DC)	1.29	1.81E-02	7.42E-02	0
Lipid	Dihydrosphingomyelins	sphingomyelin (d18:0/18:0, d19:0/17:0)*	1.28	1.81E-02	7.42E-02	0
Lipid	Phospholipid Metabolism	phosphoethanolamine	1.26	2.01E-02	8.16E-02	0
Lipid	Diacylglycerol	palmitoyl-arachidonoyl-glycerol (16:0/20:4) [2]*	0.79	2.10E-02	8.34E-02	0
Lipid	Primary Bile Acid Metabolism	glycochenodeoxycholate glucuronide (1)	1.27	2.10E-02	8.34E-02	0
Lipid	Fatty Acid, Monohydroxy	2-hydroxystearate	1.26	2.22E-02	8.61E-02	0
Lipid	Sphingomyelins	sphingomyelin (d18:1/20:0, d16:1/22:0)*	1.27	2.26E-02	8.67E-02	0
Lipid	Fatty Acid Metabolism(Acyl Carnitine)	lignoceroylcarnitine (C24)*	0.80	2.67E-02	9.99E-02	0
Lipid	Polyunsaturated Fatty Acid (n3 and n6)	docosadienoate (22:2n6)	1.25	3.30E-02	1.17E-01	0
Lipid	Polyunsaturated Fatty Acid (n3 and n6)	stearidonate (18:4n3)	1.25	3.74E-02	1.30E-01	0

Lipid	Polyunsaturated Fatty Acid (n3 and n6)	dihomo-linolenate (20:3n3 or n6)	1.23	3.85E-02	1.32E-01	0
Lipid	Fatty Acid, Amino	2-aminoheptanoate	0.81	4.07E-02	1.37E-01	0
Lipid	Fatty Acid, Dicarboxylate	octadecanedioate (C18-DC)	1.24	4.28E-02	1.42E-01	0
Lipid	Dihydroceramides	N-palmitoyl-sphinganine (d18:0/16:0)	1.22	4.79E-02	1.55E-01	0
Nucleotide	Pyrimidine Metabolism, Cytidine containing	cytidine	1.69	1.00E-06	2.72E-05	1
Nucleotide	Purine Metabolism, Adenine containing	adenosine	1.47	7.00E-05	1.19E-03	1
Nucleotide	Purine Metabolism, (Hypo)Xanthine/Inosine containing	inosine	1.37	1.79E-03	1.26E-02	1
Nucleotide	Purine Metabolism, (Hypo)Xanthine/Inosine containing	allantoin	0.72	5.15E-03	2.94E-02	1
Nucleotide	Pyrimidine Metabolism, Thymine containing	5,6-dihydrothymine	1.33	7.39E-03	3.79E-02	1
Nucleotide	Purine Metabolism, Adenine containing	adenine	1.31	7.52E-03	3.79E-02	1
Nucleotide	Purine Metabolism, Guanine containing	N2,N2-dimethylguanosine	1.36	1.47E-02	6.45E-02	0
Nucleotide	Pyrimidine Metabolism, Uracil containing	2'-deoxyuridine	1.24	3.52E-02	1.24E-01	0
Nucleotide	Pyrimidine Metabolism, Uracil containing	uridine	0.82	4.36E-02	1.44E-01	0
Nucleotide	Purine Metabolism, (Hypo)Xanthine/Inosine containing	urate	0.80	4.47E-02	1.45E-01	0
Peptide	Acetylated Peptides	phenylacetylglutamine	1.45	2.20E-04	2.60E-03	1
Peptide	Gamma-glutamyl Amino Acid	gamma-glutamylglutamine	0.67	3.70E-04	3.73E-03	1
Peptide	Acetylated Peptides	4-hydroxyphenylacetylglutamine	1.34	6.61E-03	3.53E-02	1
Peptide	Gamma-glutamyl Amino Acid	gamma-glutamylhistidine	0.77	7.60E-03	3.79E-02	1
Peptide	Gamma-glutamyl Amino Acid	gamma-glutamylalanine	0.81	3.89E-02	1.33E-01	0

Legend: OR – Odds Ratio; Pval – P-value; Qval – q-value; US – United States; Qval_Sig – indicator for significance based on q-value.

Supplementary Table 6. Metabolites differing significantly across all study groups using Coimbra (Portugal) samples based on p-values from Stage+2Eye model.

Super Pathway	Sub Pathway	Metabolite	OR PT	Pval PT	Qval PT	Qval Sig
Amino Acid	Methionine, Cysteine, SAM and Taurine Metabolism	hypotaurine	1.49	1.00E-06	1.36E-04	1
Amino Acid	Glutamate Metabolism	glutamate	1.44	3.00E-05	1.63E-03	1
Amino Acid	Alanine and Aspartate Metabolism	aspartate	1.37	7.00E-05	3.17E-03	1
Amino Acid	Methionine, Cysteine, SAM and Taurine Metabolism	N-acetylmethionine	1.35	3.60E-04	1.31E-02	1
Amino Acid	Methionine, Cysteine, SAM and Taurine Metabolism	taurine	1.31	5.30E-04	1.80E-02	1
Amino Acid	Tryptophan Metabolism	indoleacetylglutamine	0.78	1.46E-03	3.31E-02	1
Amino Acid	Urea cycle; Arginine and Proline Metabolism	trans-4-hydroxyproline	1.28	2.09E-03	4.55E-02	1
Amino Acid	Glutamate Metabolism	beta-citrylglutamate	1.26	3.40E-03	6.38E-02	0
Amino Acid	Methionine, Cysteine, SAM and Taurine Metabolism	S-adenosylhomocysteine (SAH)	1.26	5.91E-03	9.07E-02	0
Amino Acid	Urea cycle; Arginine and Proline Metabolism	2-oxoarginine*	1.26	6.00E-03	9.07E-02	0
Amino Acid	Glutamate Metabolism	N-acetylglutamate	1.24	1.15E-02	1.38E-01	0
Amino Acid	Alanine and Aspartate Metabolism	N-acetylasparagine	1.23	1.16E-02	1.38E-01	0
Amino Acid	Glycine, Serine and Threonine Metabolism	threonine	1.23	1.19E-02	1.38E-01	0
Amino Acid	Polyamine Metabolism	spermidine	1.21	1.57E-02	1.65E-01	0
Amino Acid	Urea cycle; Arginine and Proline Metabolism	N-methylproline	0.83	1.65E-02	1.67E-01	0
Amino Acid	Tryptophan Metabolism	serotonin	1.20	1.71E-02	1.69E-01	0
Amino Acid	Methionine, Cysteine, SAM and Taurine Metabolism	methionine	1.21	2.09E-02	1.84E-01	0
Amino Acid	Tryptophan Metabolism	indoleacetate	0.84	2.34E-02	1.99E-01	0
Amino Acid	Glutamate Metabolism	carboxyethyl-GABA	0.83	2.47E-02	2.04E-01	0
Amino Acid	Glycine, Serine and Threonine Metabolism	betaine	0.83	2.74E-02	2.13E-01	0
Amino Acid	Tyrosine Metabolism	4-hydroxyphenylpyruvate	1.19	2.93E-02	2.18E-01	0
Amino Acid	Glutamate Metabolism	pyroglutamine*	0.81	2.96E-02	2.18E-01	0
Amino Acid	Leucine, Isoleucine and Valine Metabolism	isovalerylcarnitine (C5)	1.19	3.04E-02	2.18E-01	0
Amino Acid	Urea cycle; Arginine and Proline Metabolism	arginine	1.19	3.09E-02	2.18E-01	0
Amino Acid	Creatine Metabolism	creatine	1.21	3.36E-02	2.32E-01	0
Amino Acid	Glutamate Metabolism	glutamine	0.85	3.98E-02	2.71E-01	0
Amino Acid	Glutathione Metabolism	cys-gly, oxidized	0.85	4.37E-02	2.89E-01	0
Amino Acid	Alanine and Aspartate Metabolism	asparagine	1.17	4.42E-02	2.89E-01	0
Amino Acid	Leucine, Isoleucine and Valine Metabolism	N-acetylleucine	1.18	4.62E-02	2.95E-01	0
Carbohydrate	Glycogen Metabolism	maltotriose	1.43	1.00E-06	1.36E-04	1
Carbohydrate	Glycolysis, Gluconeogenesis, and Pyruvate Metabolism	pyruvate	1.41	1.00E-05	9.07E-04	1
Carbohydrate	Glycogen Metabolism	maltose	1.38	3.00E-05	1.63E-03	1
Carbohydrate	Glycolysis, Gluconeogenesis, and Pyruvate Metabolism	1,5-anhydroglucitol (1,5-AG)	0.72	4.00E-05	1.98E-03	1
Carbohydrate	Aminosugar Metabolism	N-acetylneuraminate	1.34	7.00E-04	2.06E-02	1
Carbohydrate	Glycolysis, Gluconeogenesis, and Pyruvate Metabolism	lactate	1.24	1.07E-02	1.38E-01	0
Cofactors and Vitamins	Nicotinate and Nicotinamide Metabolism	nicotinamide	1.30	1.03E-03	2.55E-02	1
Cofactors and Vitamins	Tocopherol Metabolism	gamma-CEHC	1.21	1.18E-02	1.38E-01	0
Energy	TCA Cycle	malate	1.25	9.73E-03	1.29E-01	0
Lipid	Phosphatidylserine (PS)	1-stearoyl-2-oleoyl-GPS (18:0/18:1)	1.50	1.00E-06	1.36E-04	1
Lipid	Phospholipid Metabolism	phosphoethanolamine	1.39	2.00E-05	1.55E-03	1
Lipid	Sphingosines	sphingosine 1-phosphate	1.40	3.00E-05	1.63E-03	1
Lipid	Phospholipid Metabolism	choline phosphate	1.34	2.20E-04	9.21E-03	1
Lipid	Sphingolipid Synthesis	sphinganine	1.34	2.80E-04	1.09E-02	1
Lipid	Eicosanoid	12-HETE	1.31	6.00E-04	1.92E-02	1
Lipid	Fatty Acid, Monohydroxy	14-HDoHE/17-HDoHE	1.30	7.20E-04	2.06E-02	1
Lipid	Sphingosines	sphingosine	1.29	9.70E-04	2.51E-02	1
Lipid	Lysophospholipid	1-linoleoyl-GPE (18:2)*	1.30	1.37E-03	3.24E-02	1
Lipid	Fatty Acid Metabolism (Acyl Choline)	docosahexaenoylcholine	0.79	2.30E-03	4.63E-02	1

Lipid	Lysophospholipid	1-oleoyl-GPE (18:1)	1.26	4.56E-03	7.96E-02	0
Lipid	Monoacylglycerol	2-oleoylglycerol (18:1)	0.80	4.68E-03	7.96E-02	0
Lipid	Fatty Acid Metabolism(Acyl Carnitine)	dihomo-linolenoylcarnitine (C20:3n3 or 6)*	1.26	5.07E-03	8.36E-02	0
Lipid	Polyunsaturated Fatty Acid (n3 and n6)	docosahexaenoate (DHA; 22:6n3)	0.80	5.53E-03	8.85E-02	0
Lipid	Phosphatidylcholine (PC)	1,2-dipalmitoyl-GPC (16:0/16:0)	1.25	7.10E-03	1.04E-01	0
Lipid	Primary Bile Acid Metabolism	glycocholate	1.25	9.30E-03	1.29E-01	0
Lipid	Sphingomyelins	lignoceroyl sphingomyelin (d18:1/24:0)	1.24	9.52E-03	1.29E-01	0
Lipid	Sphingomyelins	behenoyl sphingomyelin (d18:1/22:0)*	1.24	9.56E-03	1.29E-01	0
Lipid	Secondary Bile Acid Metabolism	glycolithocholate sulfate*	1.22	1.19E-02	1.38E-01	0
Lipid	Fatty Acid Metabolism(Acyl Carnitine)	dihomo-linoleoylcarnitine (C20:2)*	1.22	1.23E-02	1.39E-01	0
Lipid	Primary Bile Acid Metabolism	glycochenodeoxycholate	1.22	1.42E-02	1.55E-01	0
Lipid	Phospholipid Metabolism	glycerophosphorylcholine (GPC)	0.83	1.55E-02	1.65E-01	0
Lipid	Lysoplasmalogen	1-(1-enyl-palmitoyl)-GPE (P-16:0)*	1.21	1.62E-02	1.66E-01	0
Lipid	Pregnenolone Steroids	pregnenolone sulfate	1.22	1.87E-02	1.79E-01	0
Lipid	Primary Bile Acid Metabolism	taurochenodeoxycholate	1.21	1.93E-02	1.79E-01	0
Lipid	Medium Chain Fatty Acid	caprylate (8:0)	1.21	1.98E-02	1.79E-01	0
Lipid	Fatty Acid, Dicarboxylate	3-methyladipate	0.83	2.09E-02	1.84E-01	0
Lipid	Phosphatidylcholine (PC)	1-palmitoyl-2-palmitoleoyl-GPC (16:0/16:1)*	1.20	2.32E-02	1.99E-01	0
Lipid	Pregnenolone Steroids	pregnenediol sulfate (C21H34O5S)*	1.22	2.45E-02	2.04E-01	0
Lipid	Fatty Acid Metabolism(Acyl Carnitine)	linoleoylcarnitine (C18:2)*	1.20	2.53E-02	2.05E-01	0
Lipid	Medium Chain Fatty Acid	10-undecenoate (11:1n1)	0.84	2.56E-02	2.05E-01	0
Lipid	Phosphatidylethanolamine (PE)	1-stearoyl-2-oleoyl-GPE (18:0/18:1)	1.20	2.68E-02	2.11E-01	0
Lipid	Fatty Acid Metabolism(Acyl Carnitine)	stearoylcarnitine (C18)	1.19	2.79E-02	2.14E-01	0
Lipid	Fatty Acid Metabolism (also BCAA Metabolism)	propionylcarnitine (C3)	1.20	2.99E-02	2.18E-01	0
Lipid	Phosphatidylcholine (PC)	1-palmitoyl-2-linoleoyl-GPC (16:0/18:2)	1.19	3.07E-02	2.18E-01	0
Lipid	Monoacylglycerol	1-docosahexaenoylglycerol (22:6)	0.85	4.17E-02	2.80E-01	0
Lipid	Dihydrosphingomyelins	sphingomyelin (d18:0/18:0, d19:0/17:0)*	1.18	4.47E-02	2.89E-01	0
Lipid	Sterol	3beta,7alpha-dihydroxy-5-cholestenoate	0.84	4.67E-02	2.95E-01	0
Lipid	Dihydrosphingomyelins	sphingomyelin (d18:0/20:0, d16:0/22:0)*	1.18	4.82E-02	3.02E-01	0
Nucleotide	Pyrimidine Metabolism, Orotate containing	dihydroorotate	0.71	1.00E-06	1.36E-04	1
Nucleotide	Purine Metabolism, (Hypo)Xanthine/Inosine containing	hypoxanthine	1.40	1.00E-05	9.07E-04	1
Nucleotide	Pyrimidine Metabolism, Cytidine containing	cytidine	1.31	9.10E-04	2.48E-02	1
Nucleotide	Purine Metabolism, Adenine containing	adenosine	1.25	3.12E-03	6.06E-02	0
Nucleotide	Purine Metabolism, Guanine containing	guanine	1.25	4.64E-03	7.96E-02	0
Nucleotide	Pyrimidine Metabolism, Uracil containing	uracil	1.21	1.32E-02	1.47E-01	0
Nucleotide	Pyrimidine Metabolism, Orotate containing	orotidine	0.82	1.74E-02	1.69E-01	0
Nucleotide	Purine Metabolism, Adenine containing	N6-succinyladenosine	0.82	1.94E-02	1.79E-01	0
Nucleotide	Pyrimidine Metabolism, Uracil containing	beta-alanine	1.19	3.13E-02	2.19E-01	0
Peptide	Dipeptide	isoleucylglycine	1.27	2.23E-03	4.63E-02	1

Legend: OR – Odds Ratio; Pval – P-value; Qval – q-value; PT– Portugal; Qval_Sig – indicator for significance based on q-value.

Supplementary Table 7. Metabolites differing significantly (p-value) from Stage+2Eye model identified on the meta-analysis of the 2 cohorts.

Super Pathway	Sub Pathway	Metabolite	OR PT	Pval PT	OR US	Pval US	OR Meta	Pval Meta	Qval Meta	Qval Sig
Amino Acid	Alanine and Aspartate Metabolism	N-acetylasparagine	1.23	1.16E-02	1.74	1.00E-06	1.37	6.10E-07	1.65E-04	1
Amino Acid	Methionine, Cysteine, SAM and Taurine Metabolism	hypotaurine	1.49	1.00E-06	1.19	1.08E-01	1.37	1.21E-06	1.65E-04	1
Amino Acid	Glutamate Metabolism	beta-citrylglutamate	1.26	3.40E-03	1.46	3.50E-04	1.32	6.67E-06	4.21E-04	1
Amino Acid	Leucine, Isoleucine and Valine Metabolism	N-acetylleucine	1.18	4.62E-02	1.43	7.30E-04	1.27	2.69E-04	7.80E-03	1
Amino Acid	Tryptophan Metabolism	serotonin	1.20	1.71E-02	1.32	5.54E-03	1.23	3.40E-04	7.96E-03	1
Amino Acid	Methionine, Cysteine, SAM and Taurine Metabolism	taurine	1.31	5.30E-04	1.14	1.73E-01	1.25	3.51E-04	7.96E-03	1
Amino Acid	Urea cycle; Arginine and Proline Metabolism	2-oxoarginine*	1.26	6.00E-03	1.25	2.87E-02	1.25	4.45E-04	9.31E-03	1
Amino Acid	Alanine and Aspartate Metabolism	aspartate	1.37	7.00E-05	1.05	6.37E-01	1.25	5.93E-04	1.11E-02	1
Amino Acid	Urea cycle; Arginine and Proline Metabolism	trans-4-hydroxyproline	1.28	2.09E-03	1.18	1.07E-01	1.24	6.25E-04	1.13E-02	1
Amino Acid	Methionine, Cysteine, SAM and Taurine Metabolism	N-acetylmethionine	1.35	3.60E-04	1.09	4.05E-01	1.25	8.60E-04	1.37E-02	1
Amino Acid	Leucine, Isoleucine and Valine Metabolism	isovalerate (i5:0)	1.03	6.73E-01	1.75	1.00E-06	1.26	8.82E-04	1.37E-02	1
Amino Acid	Glutamate Metabolism	glutamine	0.85	3.98E-02	0.74	6.07E-03	0.81	9.51E-04	1.40E-02	1
Amino Acid	Glutathione Metabolism	cys-gly, oxidized	0.85	4.37E-02	0.75	6.55E-03	0.82	1.12E-03	1.52E-02	1
Amino Acid	Histidine Metabolism	histidine	0.98	7.77E-01	0.52	1.00E-06	0.79	1.30E-03	1.73E-02	1
Amino Acid	Urea cycle; Arginine and Proline Metabolism	N-acetylarginine	1.13	1.33E-01	1.40	1.40E-03	1.21	1.67E-03	2.03E-02	1
Amino Acid	Methionine, Cysteine, SAM and Taurine Metabolism	S-adenosylhomocysteine (SAH)	1.26	5.91E-03	1.20	1.15E-01	1.23	1.68E-03	2.03E-02	1
Amino Acid	Methionine, Cysteine, SAM and Taurine Metabolism	S-methylcysteine	0.96	6.43E-01	0.64	2.00E-05	0.86	2.94E-03	3.13E-02	1
Amino Acid	Leucine, Isoleucine and Valine Metabolism	isobutyrylglycine	1.11	1.91E-01	1.40	1.67E-03	1.21	3.11E-03	3.25E-02	1
Amino Acid	Tyrosine Metabolism	p-cresol glucuronide*	1.10	2.59E-01	1.40	1.62E-03	1.19	4.80E-03	4.21E-02	1
Amino Acid	Glutathione Metabolism	cysteinylglycine	0.94	4.46E-01	0.69	3.40E-04	0.84	5.20E-03	4.30E-02	1
Amino Acid	Tyrosine Metabolism	N-acetyltyrosine	1.12	1.63E-01	1.32	8.54E-03	1.18	6.70E-03	5.28E-02	0
Amino Acid	Glycine, Serine and Threonine Metabolism	betaine	0.83	2.74E-02	0.85	1.21E-01	0.84	7.06E-03	5.33E-02	0
Amino Acid	Glutamate Metabolism	N-acetylglutamate	1.24	1.15E-02	1.13	2.70E-01	1.19	7.46E-03	5.49E-02	0
Amino Acid	Urea cycle; Arginine and Proline Metabolism	N-methylproline	0.83	1.65E-02	0.88	2.22E-01	0.85	8.23E-03	5.81E-02	0
Amino Acid	Methionine, Cysteine, SAM and Taurine Metabolism	methionine sulfoxide	1.13	1.21E-01	1.25	2.79E-02	1.17	1.02E-02	6.59E-02	0
Amino Acid	Methionine, Cysteine, SAM and Taurine Metabolism	S-methylcysteine sulfoxide	0.96	5.83E-01	0.70	7.90E-04	0.85	1.29E-02	7.65E-02	0
Amino Acid	Leucine, Isoleucine and Valine Metabolism	N-acetylisoleucine	1.13	1.50E-01	1.30	2.83E-02	1.20	1.32E-02	7.70E-02	0
Amino Acid	Urea cycle; Arginine and Proline Metabolism	N-acetylcitrulline	1.07	3.95E-01	1.36	3.83E-03	1.16	1.46E-02	8.11E-02	0
Amino Acid	Tyrosine Metabolism	4-hydroxyphenylpyruvate	1.19	2.93E-02	1.12	2.61E-01	1.17	1.59E-02	8.60E-02	0
Amino Acid	Histidine Metabolism	3-methylhistidine	1.10	2.35E-01	1.27	2.24E-02	1.16	1.94E-02	9.69E-02	0
Amino Acid	Phenylalanine Metabolism	N-acetylphenylalanine	1.13	1.50E-01	1.24	5.37E-02	1.16	2.04E-02	1.00E-01	0
Amino Acid	Methionine, Cysteine, SAM and Taurine Metabolism	alpha-ketobutyrate	1.16	6.78E-02	1.16	1.56E-01	1.16	2.07E-02	1.01E-01	0
Amino Acid	Tryptophan Metabolism	indoleacetylglutamine	0.78	1.46E-03	1.04	7.29E-01	0.86	2.11E-02	1.01E-01	0
Amino Acid	Methionine, Cysteine, SAM and Taurine Metabolism	cysteine sulfinic acid	1.12	1.66E-01	1.22	4.92E-02	1.16	2.15E-02	1.02E-01	0
Amino Acid	Lysine Metabolism	N6-acetyllysine	1.14	1.16E-01	1.21	9.21E-02	1.15	2.31E-02	1.07E-01	0
Amino Acid	Leucine, Isoleucine and Valine Metabolism	isovalerylglycine	1.03	6.78E-01	1.39	1.72E-03	1.18	2.48E-02	1.12E-01	0
Amino Acid	Leucine, Isoleucine and Valine Metabolism	N-acetylvaline	1.10	2.70E-01	1.29	2.50E-02	1.17	2.49E-02	1.12E-01	0
Amino Acid	Glycine, Serine and Threonine Metabolism	dimethylglycine	0.92	3.07E-01	0.77	2.13E-02	0.87	2.67E-02	1.18E-01	0
Amino Acid	Creatine Metabolism	creatine	1.21	3.36E-02	1.10	3.98E-01	1.16	2.80E-02	1.21E-01	0
Amino Acid	Glycine, Serine and Threonine Metabolism	threonine	1.23	1.19E-02	1.02	8.14E-01	1.14	3.29E-02	1.40E-01	0
Amino Acid	Methionine, Cysteine, SAM and Taurine Metabolism	N-formylmethionine	1.15	1.21E-01	1.18	1.52E-01	1.16	3.55E-02	1.45E-01	0
Amino Acid	Methionine, Cysteine, SAM and Taurine Metabolism	S-methylmethionine	0.93	3.51E-01	0.79	2.68E-02	0.87	3.64E-02	1.48E-01	0
Amino Acid	Glutamate Metabolism	glutamate	1.44	3.00E-05	0.81	4.08E-02	1.16	4.02E-02	1.57E-01	0
Amino Acid	Tyrosine Metabolism	phenol sulfate	0.88	1.00E-01	0.88	2.26E-01	0.88	4.13E-02	1.60E-01	0
Carbohydrate	Glycogen Metabolism	maltotriose	1.43	1.00E-06	1.11	2.88E-01	1.31	6.19E-06	4.21E-04	1
Carbohydrate	Glycogen Metabolism	maltose	1.38	3.00E-05	1.15	1.60E-01	1.29	3.17E-05	1.72E-03	1
Carbohydrate	Aminosugar Metabolism	N-acetylneuraminate	1.34	7.00E-04	1.08	4.70E-01	1.25	1.78E-03	2.09E-02	1
Carbohydrate	Glycolysis, Gluconeogenesis, and Pyruvate Metabolism	pyruvate	1.41	1.00E-05	0.91	3.61E-01	1.18	3.31E-03	3.28E-02	1

Carbohydrate	Glycolysis, Gluconeogenesis, and Pyruvate Metabolism	1,5-anhydroglucitol (1,5-AG)	0.72	4.00E-05	1.12	2.79E-01	0.84	9.64E-03	6.39E-02	0
Carbohydrate	Aminosugar Metabolism	N-acetylglucosamine/N-acetylgalactosamine	1.03	7.40E-01	1.52	2.20E-04	1.20	1.17E-02	7.21E-02	0
Cofactors and Vitamins	Tocopherol Metabolism	alpha-tocopherol	1.03	7.00E-01	2.00	1.00E-06	1.42	9.81E-04	1.40E-02	1
Cofactors and Vitamins	Hemoglobin and Porphyrin Metabolism	biliverdin	1.03	6.91E-01	1.52	1.60E-04	1.17	8.72E-03	5.93E-02	0
Cofactors and Vitamins	Nicotinate and Nicotinamide Metabolism	nicotinamide	1.30	1.03E-03	0.97	7.44E-01	1.18	1.65E-02	8.65E-02	0
Energy	TCA Cycle	malate	1.25	9.73E-03	1.25	3.58E-02	1.25	8.70E-04	1.37E-02	1
Lipid	Lysoplasmalogen	1-(1-enyl-palmitoyl)-GPE (P-16:0)*	1.21	1.62E-02	1.95	1.00E-06	1.48	9.86E-07	1.65E-04	1
Lipid	Phospholipid Metabolism	phosphoethanolamine	1.39	2.00E-05	1.26	2.01E-02	1.32	1.62E-06	1.67E-04	1
Lipid	Diacylglycerol	oleoyl-oleoyl-glycerol (18:1/18:1) [2]*	0.86	5.74E-02	0.62	1.00E-06	0.76	6.96E-06	4.21E-04	1
Lipid	Diacylglycerol	oleoyl-linoleoyl-glycerol (18:1/18:2) [1]	0.87	7.56E-02	0.64	1.00E-05	0.76	4.01E-05	1.98E-03	1
Lipid	Fatty Acid Metabolism(Acyl Carnitine)	myristoleoylcarnitine (C14:1)*	0.90	2.06E-01	0.62	1.00E-06	0.78	6.54E-05	2.97E-03	1
Lipid	Phosphatidylserine (PS)	1-stearoyl-2-oleoyl-GPS (18:0/18:1)	1.50	1.00E-06	1.01	9.33E-01	1.33	8.78E-05	3.54E-03	1
Lipid	Fatty Acid Metabolism(Acyl Carnitine)	laurylcarnitine (C12)	0.91	2.78E-01	0.61	1.00E-06	0.78	1.18E-04	4.29E-03	1
Lipid	Diacylglycerol	oleoyl-linoleoyl-glycerol (18:1/18:2) [2]	0.89	1.44E-01	0.63	2.00E-05	0.76	1.68E-04	5.67E-03	1
Lipid	Fatty Acid Metabolism(Acyl Carnitine)	decanoylcarnitine (C10)	0.86	5.97E-02	0.69	2.20E-04	0.79	1.77E-04	5.67E-03	1
Lipid	Lysoplasmalogen	1-(1-enyl-oleoyl)-GPE (P-18:1)*	1.06	4.19E-01	1.75	1.00E-06	1.32	2.83E-04	7.80E-03	1
Lipid	Diacylglycerol	oleoyl-oleoyl-glycerol (18:1/18:1) [1]*	0.86	5.14E-02	0.70	6.50E-04	0.79	2.87E-04	7.80E-03	1
Lipid	Lysoplasmalogen	1-(1-enyl-stearoyl)-GPE (P-18:0)*	1.06	4.36E-01	1.95	1.00E-06	1.38	3.08E-04	7.96E-03	1
Lipid	Fatty Acid Metabolism(Acyl Carnitine)	docosahexaenoylcarnitine (C22:6)*	0.86	5.58E-02	0.70	7.20E-04	0.79	3.42E-04	7.96E-03	1
Lipid	Plasmalogen	1-(1-enyl-palmitoyl)-2-arachidonoyl-GPE (P-16:0/20:4)*	1.13	1.41E-01	1.50	9.00E-05	1.24	3.70E-04	8.06E-03	1
Lipid	Diacylglycerol	oleoyl-arachidonoyl-glycerol (18:1/20:4) [2]*	0.95	5.24E-01	0.63	1.00E-06	0.81	4.74E-04	9.56E-03	1
Lipid	Fatty Acid Metabolism(Acyl Carnitine)	octanoylcarnitine (C8)	0.87	8.85E-02	0.71	6.10E-04	0.81	5.74E-04	1.11E-02	1
Lipid	Eicosanoid	12-HETE	1.31	6.00E-04	1.11	2.85E-01	1.24	7.55E-04	1.32E-02	1
Lipid	Phospholipid Metabolism	choline phosphate	1.34	2.20E-04	1.07	5.22E-01	1.24	9.17E-04	1.39E-02	1
Lipid	Plasmalogen	1-(1-enyl-palmitoyl)-2-linoleoyl-GPE (P-16:0/18:2)*	1.05	5.27E-01	1.62	1.00E-05	1.22	1.37E-03	1.77E-02	1
Lipid	Diacylglycerol	palmitoyl-linoleoyl-glycerol (16:0/18:2) [2]*	0.92	3.05E-01	0.67	1.60E-04	0.80	1.81E-03	2.09E-02	1
Lipid	Sphingomyelins	stearoyl sphingomyelin (d18:1/18:0)	1.01	8.79E-01	1.74	1.00E-06	1.27	1.86E-03	2.11E-02	1
Lipid	Lysophospholipid	1-linoleoyl-GPG (18:2)*	1.10	2.14E-01	1.44	5.70E-04	1.21	2.00E-03	2.22E-02	1
Lipid	Dihydrosphingomyelins	sphingomyelin (d18:0/18:0, d19:0/17:0)*	1.18	4.47E-02	1.28	1.81E-02	1.22	2.42E-03	2.63E-02	1
Lipid	Diacylglycerol	linoleoyl-docosahexaenoyl-glycerol (18:2/22:6) [2]*	0.87	8.56E-02	0.75	9.49E-03	0.84	3.21E-03	3.28E-02	1
Lipid	Secondary Bile Acid Metabolism	glycolithocholate sulfate*	1.22	1.19E-02	1.18	1.21E-01	1.21	3.30E-03	3.28E-02	1
Lipid	Fatty Acid Metabolism(Acyl Carnitine)	cerotoylcarnitine (C26)*	0.90	2.06E-01	0.73	1.79E-03	0.82	3.61E-03	3.45E-02	1
Lipid	Secondary Bile Acid Metabolism	taurolithocholate 3-sulfate	1.15	8.30E-02	1.30	1.19E-02	1.20	3.62E-03	3.45E-02	1
Lipid	Sphingomyelins	sphingomyelin (d18:2/24:2)*	1.11	2.33E-01	1.39	1.44E-03	1.21	3.83E-03	3.59E-02	1
Lipid	Lysophospholipid	1-palmitoyl-GPG (16:0)*	1.07	4.16E-01	1.45	2.60E-04	1.21	4.02E-03	3.70E-02	1
Lipid	Fatty Acid Metabolism(Acyl Carnitine)	palmitoleoylcarnitine (C16:1)*	1.01	8.76E-01	0.62	1.00E-06	0.83	4.13E-03	3.75E-02	1
Lipid	Phosphatidylinositol (PI)	1-stearoyl-2-linoleoyl-GPI (18:0/18:2)	0.94	4.31E-01	0.66	2.50E-04	0.81	4.20E-03	3.75E-02	1
Lipid	Sphingomyelins	behenoyl sphingomyelin (d18:1/22:0)*	1.24	9.56E-03	1.14	2.18E-01	1.20	5.06E-03	4.30E-02	1
Lipid	Primary Bile Acid Metabolism	glycocholate	1.25	9.30E-03	1.13	2.29E-01	1.18	5.21E-03	4.30E-02	1
Lipid	Fatty Acid Metabolism (Acyl Choline)	docosahexaenoylcholine	0.79	2.30E-03	0.94	5.60E-01	0.85	5.63E-03	4.57E-02	1
Lipid	Fatty Acid Metabolism(Acyl Carnitine)	myristoylcarnitine (C14)	0.97	7.60E-01	0.63	5.00E-05	0.81	6.49E-03	5.19E-02	0
Lipid	Fatty Acid Metabolism (also BCAA Metabolism)	propionylcarnitine (C3)	1.20	2.99E-02	1.18	1.08E-01	1.19	6.93E-03	5.33E-02	0
Lipid	Primary Bile Acid Metabolism	taurochenodeoxycholate	1.21	1.93E-02	1.14	1.67E-01	1.18	7.01E-03	5.33E-02	0
Lipid	Diacylglycerol	palmitoyl-oleoyl-glycerol (16:0/18:1) [2]*	0.95	5.16E-01	0.69	3.90E-04	0.84	7.29E-03	5.44E-02	0
Lipid	Secondary Bile Acid Metabolism	taurodeoxycholate	1.18	5.45E-02	1.20	6.03E-02	1.18	7.59E-03	5.50E-02	0
Lipid	Sphingomyelins	palmitoyl sphingomyelin (d18:1/16:0)	1.03	7.62E-01	1.50	8.00E-05	1.17	8.01E-03	5.73E-02	0
Lipid	Diacylglycerol	palmitoleoyl-linoleoyl-glycerol (16:1/18:2) [1]*	0.93	3.76E-01	0.71	1.54E-03	0.83	8.36E-03	5.83E-02	0
Lipid	Lysoplasmalogen	1-(1-enyl-palmitoyl)-GPC (P-16:0)*	0.96	6.41E-01	1.80	1.00E-06	1.32	8.74E-03	5.93E-02	0
Lipid	Fatty Acid Metabolism(Acyl Carnitine)	oleoylcarnitine (C18:1)	1.04	6.37E-01	0.60	1.00E-06	0.82	8.84E-03	5.93E-02	0
Lipid	Diacylglycerol	palmitoyl-linoleoyl-glycerol (16:0/18:2) [1]*	0.93	3.76E-01	0.73	2.21E-03	0.84	1.01E-02	6.59E-02	0
Lipid	Diacylglycerol	oleoyl-arachidonoyl-glycerol (18:1/20:4) [1]*	0.93	3.93E-01	0.73	2.29E-03	0.85	1.11E-02	7.01E-02	0

Lipid	Dihydrosphingomyelins	behenoyl dihydrosphingomyelin (d18:0/22:0)*	1.17	6.08E-02	1.20	8.41E-02	1.18	1.11E-02	7.01E-02	0
Lipid	Medium Chain Fatty Acid	caprylate (8:0)	1.21	1.98E-02	1.11	2.63E-01	1.16	1.14E-02	7.15E-02	0
Lipid	Sphingomyelins	sphingomyelin (d18:1/22:2, d18:2/22:1, d16:1/24:2)*	1.04	6.83E-01	1.46	3.50E-04	1.20	1.21E-02	7.39E-02	0
Lipid	Lysophospholipid	1-linoleoyl-GPE (18:2)*	1.30	1.37E-03	0.99	9.58E-01	1.16	1.24E-02	7.50E-02	0
Lipid	Phosphatidylcholine (PC)	1-stearoyl-2-arachidonoyl-GPC (18:0/20:4)	0.99	9.46E-01	0.65	7.00E-05	0.82	1.29E-02	7.65E-02	0
Lipid	Dihydrosphingomyelins	palmitoyl dihydrosphingomyelin (d18:0/16:0)*	1.07	3.96E-01	1.36	3.39E-03	1.18	1.38E-02	7.90E-02	0
Lipid	Sphingomyelins	lignoceroyl sphingomyelin (d18:1/24:0)	1.24	9.52E-03	1.07	5.05E-01	1.17	1.39E-02	7.90E-02	0
Lipid	Sphingomyelins	sphingomyelin (d18:1/20:0, d16:1/22:0)*	1.12	1.78E-01	1.27	2.26E-02	1.18	1.39E-02	7.90E-02	0
Lipid	Fatty Acid, Monohydroxy	9-hydroxystearate	1.17	5.21E-02	1.17	1.38E-01	1.17	1.45E-02	8.11E-02	0
Lipid	Monoacylglycerol	2-oleoylglycerol (18:1)	0.80	4.68E-03	0.97	7.47E-01	0.85	1.49E-02	8.18E-02	0
Lipid	Fatty Acid Metabolism(Acyl Carnitine)	cis-4-decenoylcarnitine (C10:1)	0.93	3.61E-01	0.76	5.78E-03	0.86	1.60E-02	8.60E-02	0
Lipid	Diacylglycerol	linoleoyl-arachidonoyl-glycerol (18:2/20:4) [2]*	1.00	9.54E-01	0.68	1.20E-04	0.85	1.65E-02	8.65E-02	0
Lipid	Dihydrosphingomyelins	sphingomyelin (d18:0/20:0, d16:0/22:0)*	1.18	4.82E-02	1.16	1.73E-01	1.17	1.65E-02	8.65E-02	0
Lipid	Sphingolipid Synthesis	sphinganine	1.34	2.80E-04	0.92	4.29E-01	1.20	1.68E-02	8.72E-02	0
Lipid	Hexosylceramides (HCER)	glycosyl-N-stearoyl-sphingosine (d18:1/18:0)	1.17	5.37E-02	1.16	1.63E-01	1.16	1.73E-02	8.90E-02	0
Lipid	Fatty Acid Metabolism(Acyl Carnitine)	ximenoylcarnitine (C26:1)*	1.00	9.96E-01	0.67	1.10E-04	0.84	1.82E-02	9.27E-02	0
Lipid	Primary Bile Acid Metabolism	glycochenodeoxycholate	1.22	1.42E-02	1.07	5.30E-01	1.15	2.02E-02	9.97E-02	0
Lipid	Phosphatidylinositol (PI)	1-palmitoyl-2-oleoyl-GPI (16:0/18:1)*	0.99	8.90E-01	0.68	3.30E-04	0.84	2.12E-02	1.01E-01	0
Lipid	Medium Chain Fatty Acid	10-undecenoate (11:1n1)	0.84	2.56E-02	0.92	4.03E-01	0.87	2.27E-02	1.07E-01	0
Lipid	Pregnenolone Steroids	pregnenolone sulfate	1.22	1.87E-02	1.08	5.09E-01	1.17	2.36E-02	1.09E-01	0
Lipid	Progestin Steroids	pregnanediol-3-glucuronide	1.14	1.06E-01	1.18	1.15E-01	1.15	2.49E-02	1.12E-01	0
Lipid	Diacylglycerol	stearoyl-arachidonoyl-glycerol (18:0/20:4) [1]*	0.98	8.32E-01	0.70	7.10E-04	0.88	2.52E-02	1.12E-01	0
Lipid	Lysophospholipid	1-stearoyl-GPE (18:0)	1.17	5.34E-02	1.12	2.64E-01	1.15	2.70E-02	1.18E-01	0
Lipid	Sterol	4-cholesten-3-one	1.01	8.72E-01	1.44	6.80E-04	1.15	2.74E-02	1.19E-01	0
Lipid	Lysophospholipid	1-palmitoyl-GPI (16:0)	1.03	7.09E-01	1.37	2.65E-03	1.15	3.29E-02	1.40E-01	0
Lipid	Fatty Acid, Monohydroxy	16-hydroxypalmitate	1.06	4.74E-01	1.31	1.09E-02	1.14	3.37E-02	1.42E-01	0
Lipid	Fatty Acid, Monohydroxy	14-HDoHE/17-HDoHE	1.30	7.20E-04	0.91	3.61E-01	1.18	3.42E-02	1.43E-01	0
Lipid	Sphingomyelins	sphingomyelin (d18:1/20:1, d18:2/20:0)*	0.96	6.16E-01	1.56	4.00E-05	1.18	3.45E-02	1.43E-01	0
Lipid	Phosphatidylcholine (PC)	1-stearoyl-2-oleoyl-GPC (18:0/18:1)	1.09	2.66E-01	0.53	1.00E-06	0.83	3.48E-02	1.43E-01	0
Lipid	Fatty Acid Metabolism(Acyl Carnitine)	eicosenoylcarnitine (C20:1)*	0.99	8.85E-01	0.70	1.24E-03	0.85	3.66E-02	1.48E-01	0
Lipid	Diacylglycerol	linoleoyl-linoleoyl-glycerol (18:2/18:2) [1]*	0.95	4.98E-01	0.76	1.19E-02	0.86	3.82E-02	1.53E-01	0
Lipid	Plasmalogen	1-(1-enyl-palmitoyl)-2-palmitoleoyl-GPC (P-16:0/16:1)*	1.11	1.95E-01	1.20	9.11E-02	1.14	3.95E-02	1.56E-01	0
Lipid	Primary Bile Acid Metabolism	cholate	0.91	2.07E-01	0.84	8.33E-02	0.88	3.95E-02	1.56E-01	0
Lipid	Fatty Acid, Monohydroxy	2-hydroxypalmitate	1.01	8.51E-01	1.38	2.06E-03	1.13	4.21E-02	1.61E-01	0
Lipid	Sphingosines	sphingosine	1.29	9.70E-04	0.90	3.42E-01	1.17	4.24E-02	1.61E-01	0
Lipid	Lysophospholipid	1-linoleoyl-GPI (18:2)*	1.02	8.48E-01	1.37	2.78E-03	1.14	4.77E-02	1.76E-01	0
Nucleotide	Pyrimidine Metabolism, Cytidine containing	cytidine	1.31	9.10E-04	1.69	1.00E-06	1.40	1.96E-08	1.06E-05	1
Nucleotide	Purine Metabolism, Adenine containing	adenosine	1.25	3.12E-03	1.47	7.00E-05	1.32	1.84E-06	1.67E-04	1
Nucleotide	Pyrimidine Metabolism, Orotate containing	dihydroorotate	0.71	1.00E-06	0.99	9.44E-01	0.80	9.11E-05	3.54E-03	1
Nucleotide	Purine Metabolism, (Hypo)Xanthine/Inosine containing	hypoxanthine	1.40	1.00E-05	0.98	8.27E-01	1.25	7.75E-04	1.32E-02	1
Nucleotide	Purine Metabolism, (Hypo)Xanthine/Inosine containing	inosine	1.14	8.18E-02	1.37	1.79E-03	1.17	1.01E-03	1.41E-02	1
Nucleotide	Pyrimidine Metabolism, Uracil containing	beta-alanine	1.19	3.13E-02	1.21	7.19E-02	1.20	5.05E-03	4.30E-02	1
Nucleotide	Purine Metabolism, Adenine containing	adenine	1.04	6.14E-01	1.31	7.52E-03	1.09	4.20E-02	1.61E-01	0
Nucleotide	Purine Metabolism, (Hypo)Xanthine/Inosine containing	N1-methylinosine	1.10	2.97E-01	1.25	5.32E-02	1.15	4.48E-02	1.68E-01	0
Nucleotide	Purine Metabolism, Guanine containing	guanine	1.25	4.64E-03	0.96	6.78E-01	1.16	4.70E-02	1.75E-01	0
Peptide	Acetylated Peptides	phenylacetylglutamine	1.10	2.54E-01	1.45	2.20E-04	1.21	1.57E-03	1.98E-02	1
Peptide	Gamma-glutamyl Amino Acid	gamma-glutamylglutamine	0.98	8.20E-01	0.67	3.70E-04	0.86	1.84E-02	9.27E-02	0
Peptide	Dipeptide	isoleucylglycine	1.27	2.23E-03	0.94	5.17E-01	1.16	4.31E-02	1.63E-01	0

Legend: OR – Odds Ratio; Pval – P-value; Qval – q-value; PT– Portugal; US – United States; Meta – Meta analysis; Qval_Sig - indicator for significance based on q-value.

Supplementary Table 8. List of the metabolites in Figure 2 and their p-values from AMD/Control and Stage+2Eye models from Boston cohort.

Met_Set	Met_ID	Metabolite	P_AMDControl	P_Stage2Eye
Common	X22176	cysteine s-sulfate	1.60E-05	1.00E-06
Stage+2Eye only	X39270	1-(1-enyl-palmitoyl)-GPE (P-16:0)*	2.98E-04	1.00E-06
	X39271	1-(1-enyl-stearoyl)-GPE (P-18:0)*	6.67E-04	1.00E-06
	X1561	alpha-tocopherol	1.94E-03	1.00E-06
	X52438	1-stearoyl-2-oleoyl-GPC (18:0/18:1)	4.28E-03	1.00E-06
	X52474	1-(1-enyl-palmitoyl)-GPC (P-16:0)*	8.69E-03	1.00E-06
	X59	histidine	2.77E-02	1.00E-06
	X44621	1-(1-enyl-oleoyl)-GPE (P-18:1)*	2.36E-03	1.00E-06
	X19503	stearoyl sphingomyelin (d18:1/18:0)	1.91E-01	1.00E-06
	X34534	laurylcarnitine (C12)	1.08E-02	1.00E-06
	X54946	oleoyl-oleoyl-glycerol (18:1/18:1) [2]*	3.51E-02	1.00E-06
	X54961	oleoyl-arachidonoyl-glycerol (18:1/20:4) [2]*	7.13E-03	1.00E-06
	X35160	oleoylcarnitine (C18:1)	1.27E-02	1.00E-06
	X33942	N-acetylasparagine	4.21E-03	1.00E-06
	X44656	isovalerate (i5:0)	1.24E-02	1.00E-06
	X52449	1-stearoyl-2-arachidonoyl-GPI (18:0/20:4)	1.67E-02	1.00E-06
	X514	cytidine	1.35E-03	1.00E-06
	X48182	myristoleoylcarnitine (C14:1)*	1.59E-02	1.00E-06
	X44681	palmitoylcarnitine (C16)	7.80E-03	1.00E-06
	X53223	palmitoleoylcarnitine (C16:1)*	1.50E-02	1.00E-06
	X46798	oleoyl-linoleoyl-glycerol (18:1/18:2) [1]	4.20E-02	1.00E-05
	X52677	1-(1-enyl-palmitoyl)-2-linoleoyl-GPE (P-16:0/18:2)*	6.16E-04	1.00E-05
	X46799	oleoyl-linoleoyl-glycerol (18:1/18:2) [2]	1.49E-02	2.00E-05
	X52452	1-stearoyl-2-linoleoyl-GPC (18:0/18:2)*	9.58E-02	2.00E-05
	X52461	1-palmitoyl-2-oleoyl-GPC (16:0/18:1)	6.37E-03	2.00E-05
	X57521	dihomo-linolenoylcarnitine (C20:3n3 or 6)*	4.43E-02	2.00E-05
	X39592	S-methylcysteine	1.50E-02	2.00E-05
	X48491	sphingomyelin (d18:1/20:1, d18:2/20:0)*	5.18E-02	4.00E-05
	X33952	myristoylcarnitine (C14)	6.15E-03	5.00E-05
	X52710	1-linoleoyl-2-arachidonoyl-GPC (18:2/20:4n6)*	1.22E-02	6.00E-05
	X42450	1-stearoyl-2-arachidonoyl-GPC (18:0/20:4)	1.14E-02	7.00E-05
	X555	adenosine	6.90E-04	7.00E-05
	X37506	palmitoyl sphingomyelin (d18:1/16:0)	2.64E-01	8.00E-05
	X52673	1-(1-enyl-palmitoyl)-2-arachidonoyl-GPE (P-16:0/20:4)*	3.72E-03	9.00E-05
	X36746	2-hydroxy-3-methylvalerate	1.50E-01	1.00E-04
	X42446	1-palmitoyl-2-linoleoyl-GPC (16:0/18:2)	6.38E-02	1.10E-04
	X57518	arachidonoylcarnitine (C20:4)	5.12E-02	1.10E-04
	X57517	ximenoylcarnitine (C26:1)*	4.29E-02	1.10E-04
	X54956	linoleoyl-arachidonoyl-glycerol (18:2/20:4) [2]*	3.05E-02	1.20E-04
	X52634	palmitoyl-linoleoyl-glycerol (16:0/18:2) [2]*	3.03E-02	1.60E-04
	X2137	biliverdin	3.05E-02	1.60E-04
	X52616	1-palmitoyl-2-stearoyl-GPC (16:0/18:0)	1.01E-01	1.70E-04
	X57511	linolenoylcarnitine (C18:3)*	6.81E-02	1.80E-04
	X35126	phenylacetylglutamine	1.47E-01	2.20E-04
	X33941	decanoylcarnitine (C10)	1.34E-01	2.20E-04
	X46539	N-acetylglucosamine/N-acetylgalactosamine	3.58E-01	2.20E-04
	X52468	1-stearoyl-2-linoleoyl-GPI (18:0/18:2)	1.14E-01	2.50E-04
	X45970	1-palmitoyl-GPG (16:0)*	2.10E-01	2.60E-04

X52669	1-palmitoyl-2-oleoyl-GPI (16:0/18:1)*	1.29E-01	3.30E-04
X34409	stearoylcarnitine (C18)	2.29E-02	3.40E-04
X35637	cysteinylglycine	6.33E-03	3.40E-04
X54923	beta-citrylglutamate	5.40E-02	3.50E-04
X57477	sphingomyelin (d18:1/22:2, d18:2/22:1, d16:1/24:2)*	4.71E-01	3.50E-04
X2730	gamma-glutamylglutamine	3.12E-01	3.70E-04
X54942	palmitoyl-oleoyl-glycerol (16:0/18:1) [2]*	4.67E-02	3.90E-04
X34445	sphingosine 1-phosphate	1.86E-01	4.80E-04
X54885	1-linoleoyl-GPG (18:2)*	1.90E-01	5.70E-04
X33936	octanoylcarnitine (C8)	1.50E-01	6.10E-04
X54945	oleoyl-oleoyl-glycerol (18:1/18:1) [1]*	6.11E-02	6.50E-04
X38125	4-cholesten-3-one	7.40E-02	6.80E-04
X57450	stearoyl-arachidonoyl-glycerol (18:0/20:4) [1]*	1.29E-01	7.10E-04
X57523	docosahexaenoylcarnitine (C22:6)*	2.30E-01	7.20E-04
X1587	N-acetylleucine	3.37E-01	7.30E-04
X43378	S-methylcysteine sulfoxide	1.06E-01	7.90E-04
X46223	linoleoylcarnitine (C18:2)*	1.58E-01	1.12E-03
X57531	nervonoylcarnitine (C24:1)*	2.78E-02	1.12E-03
X57519	eicosenoylcarnitine (C20:1)*	8.31E-02	1.24E-03
X33937	alpha-hydroxyisovalerate	6.74E-01	1.37E-03
X33953	N-acetylarginine	3.62E-01	1.40E-03
X57479	sphingomyelin (d18:2/24:2)*	1.70E-01	1.44E-03
X54967	palmitoleoyl-linoleoyl-glycerol (16:1/18:2) [1]*	6.93E-02	1.54E-03
X57520	dihomo-linoleoylcarnitine (C20:2)*	1.67E-01	1.56E-03
X48841	p-cresol glucuronide*	1.63E-01	1.62E-03
X35437	isobutyrylglycine	3.94E-01	1.67E-03
X35107	isovalerylglycine	2.27E-01	1.72E-03
X1123	inosine	9.69E-03	1.79E-03
X57516	cerotoylcarnitine (C26)*	1.22E-01	1.79E-03
X49617	1-lignoceroyl-GPC (24:0)	1.51E-01	1.82E-03
X35675	2-hydroxypalmitate	8.34E-01	2.06E-03
X52282	2-methylcitrate/homocitrate	6.15E-01	2.10E-03
X52470	1-palmitoyl-2-palmitoleoyl-GPC (16:0/16:1)*	2.59E-02	2.17E-03
X52633	palmitoyl-linoleoyl-glycerol (16:0/18:2) [1]*	8.58E-02	2.21E-03
X54960	oleoyl-arachidonoyl-glycerol (18:1/20:4) [1]*	7.18E-02	2.29E-03
X1110	arachidonate (20:4n6)	5.55E-01	2.46E-03
X52462	1-palmitoyl-2-arachidonoyl-GPC (16:0/20:4n6)	2.74E-02	2.55E-03
X52467	1-palmitoyl-2-arachidonoyl-GPI (16:0/20:4)*	7.90E-02	2.55E-03
X35305	1-palmitoyl-GPI (16:0)	1.73E-01	2.65E-03
X36594	1-linoleoyl-GPI (18:2)*	1.75E-01	2.78E-03
X52434	palmitoyl dihydrosphingomyelin (d18:0/16:0)*	4.15E-01	3.39E-03
X48434	N-acetylcitrulline	7.14E-01	3.83E-03
X52473	gamma-tocopherol/beta-tocopherol	2.66E-02	4.12E-03
X53176	1-linoleoyl-2-linolenoyl-GPC (18:2/18:3)*	2.29E-01	4.82E-03
X1552	erucate (22:1n9)	6.60E-01	5.05E-03
X34214	1-arachidonoyl-GPI (20:4)*	5.13E-01	5.06E-03
X1107	allantoin	1.05E-01	5.15E-03
X43591	N2,N5-diacetylornithine	9.60E-01	5.19E-03
X37190	5alpha-androstan-3beta,17beta-diol disulfate	4.72E-01	5.44E-03
X2342	serotonin	2.67E-02	5.54E-03
X38178	cis-4-decenoylcarnitine (C10:1)	2.73E-01	5.78E-03

X53	glutamine	4.81E-01	6.07E-03
X18368	cys-gly, oxidized	3.61E-02	6.55E-03
X55017	4-hydroxyphenylacetylglutamine	6.43E-01	6.61E-03
X19130	1,2-dipalmitoyl-GPC (16:0/16:0)	7.67E-02	6.78E-03
X19324	1-stearoyl-GPI (18:0)	3.49E-01	6.80E-03
X57529	docosapentaenoylcarnitine (C22:5n3)*	2.15E-01	7.26E-03
X1418	5,6-dihydrothymine	6.74E-02	7.39E-03
X554	adenine	4.38E-02	7.52E-03
X57512	margaroylcarnitine (C17)*	1.54E-02	7.59E-03
X18245	gamma-glutamylhistidine	9.09E-02	7.60E-03
X54955	linoleoyl-arachidonoyl-glycerol (18:2/20:4) [1]*	7.82E-02	7.98E-03
X32390	N-acetyltyrosine	8.01E-01	8.54E-03
X54950	linoleoyl-docosahexaenoyl-glycerol (18:2/22:6) [2]*	1.76E-01	9.49E-03
X52603	1,2-dilinoleoyl-GPC (18:2/18:2)	4.38E-01	9.55E-03
X36751	N2-acetyllysine	4.73E-01	9.89E-03

Legend: P_AMDControl - p-values from AMD/Control model; P_Stage2Eye - p-values from Stage+2Eye model

Supplementary Table 9. List of the metabolites in Figure 2 and their p-values from AMD/Control and Stage+2Eye models from Portuguese cohort.

Met_Set	Met_ID	Metabolite	P_AMDControl	P_Stage2Eye
AMD/Control only	X32352	guanine	5.99E-07	4.64E-03
	X54923	beta-citrylglutamate	1.37E-05	3.40E-03
	X555	adenosine	6.33E-05	3.12E-03
	X1123	inosine	1.54E-04	8.18E-02
	X42382	S-adenosylhomocysteine (SAH)	3.91E-04	5.91E-03
	X2342	serotonin	6.20E-04	1.71E-02
	X32412	butyrylcarnitine (C4)	1.69E-03	5.67E-01
Common	X19265	1-stearoyl-2-oleoyl-GPS (18:0/18:1)	2.94E-10	1.00E-06
	X44688	maltotriose	2.44E-07	1.00E-06
	X590	hypotaurine	4.69E-04	1.00E-06
	X601	dihydroorotate	7.52E-04	1.00E-06
	X3127	hypoxanthine	3.58E-05	1.00E-05
	X1600	phosphoethanolamine	2.36E-08	2.00E-05
	X15586	maltose	1.62E-06	3.00E-05
	X32377	N-acetylneuraminate	8.79E-05	7.00E-04
	X40008	isoleucylglycine	1.25E-04	2.23E-03
Stage+2Eye only	X42582	pyruvate	8.07E-02	1.00E-05
	X57	glutamate	3.32E-03	3.00E-05
	X20675	1,5-anhydroglucitol (1,5-AG)	7.89E-02	4.00E-05
	X36600	1-linoleoyl-GPE (18:2)*	5.48E-01	1.37E-03
	X42087	indoleacetylglutamine	6.77E-02	1.46E-03
	X32306	trans-4-hydroxyproline	8.08E-02	2.09E-03
	X53263	docosahexaenoylcholine	2.28E-01	2.30E-03

Legend: P_AMDControl - p-values from AMD/Control model; P_Stage2Eye - p-values from Stage+2Eye model

Supplementary Table 10. List of the metabolites in Figure 2 and their p-values from AMD/Control and Stage+2Eye models from the meta analysis.

Met_Set	Met_ID	Metabolite	P_AMDControl	P_Stage2Eye
AMD/Control only	X32352	guanine	8.16E-07	4.70E-02
	X17747	sphingosine	2.26E-06	4.24E-02
	X17747	14-HDoHE/17-HDoHE	3.87E-05	3.42E-02
	X17769	sphinganine	3.05E-04	1.68E-02
	X594	nicotinamide	7.87E-04	1.65E-02
	X40008	isoleucylglycine	1.15E-03	4.31E-02
	X554	adenine	2.13E-03	4.20E-02
Common	X514	cytidine	2.93E-07	1.96E-08
	X33942	N-acetylasparagine	3.22E-04	6.10E-07
	X39270	1-(1-enyl-palmitoyl)-GPE (P-16:0)*	1.12E-05	9.86E-07
	X590	hypotaurine	2.57E-04	1.21E-06
	X1600	phosphoethanolamine	2.44E-08	1.62E-06
	X555	adenosine	1.60E-07	1.84E-06
	X44688	maltotriose	1.31E-07	6.19E-06
	X54923	beta-citrylglutamate	3.86E-06	6.67E-06
	X15586	maltose	4.14E-06	3.17E-05
	X19265	1-stearoyl-2-oleoyl-GPS (18:0/18:1)	1.80E-08	8.78E-05
	X44621	1-(1-enyl-oleoyl)-GPE (P-18:1)*	1.41E-03	2.83E-04
	X39271	1-(1-enyl-stearoyl)-GPE (P-18:0)*	1.81E-03	3.08E-04
	X2342	serotonin	4.84E-05	3.40E-04
	X2125	taurine	9.59E-07	3.51E-04
	X443	aspartate	1.02E-04	5.93E-04
	X37536	12-HETE	4.03E-05	7.55E-04
	X3127	hypoxanthine	8.43E-05	7.75E-04
	X1589	N-acetylmethionine	9.74E-05	8.60E-04
	X34396	choline phosphate	1.42E-07	9.17E-04
	X1123	inosine	4.74E-06	1.01E-03
	X42382	S-adenosylhomocysteine (SAH)	6.43E-04	1.68E-03
Stage+2Eye only	X54946	oleoyl-oleoyl-glycerol (18:1/18:1) [2]*	1.37E-02	6.96E-06
	X46798	oleoyl-linoleoyl-glycerol (18:1/18:2) [1]	2.27E-02	4.01E-05
	X48182	myristoleoylcarnitine (C14:1)*	2.62E-01	6.54E-05
	X601	dihydroorotate	5.35E-03	9.11E-05
	X34534	laurylcarnitine (C12)	1.88E-01	1.18E-04
	X46799	oleoyl-linoleoyl-glycerol (18:1/18:2) [2]	2.28E-02	1.68E-04
	X33941	decanoylcarnitine (C10)	1.89E-01	1.77E-04
	X1587	N-acetylleucine	1.58E-01	2.69E-04
	X54945	oleoyl-oleoyl-glycerol (18:1/18:1) [1]*	1.79E-02	2.87E-04
	X57523	docosahexaenoylcarnitine (C22:6)*	9.95E-01	3.42E-04
	X52673	1-(1-enyl-palmitoyl)-2-arachidonoyl-GPE (P-16:0/20:4)*	2.95E-02	3.70E-04
	X55072	2-oxoarginine*	2.90E-01	4.45E-04
	X54961	oleoyl-arachidonoyl-glycerol (18:1/20:4) [2]*	1.59E-02	4.74E-04
	X33936	octanoylcarnitine (C8)	2.60E-01	5.74E-04
	X32306	trans-4-hydroxyproline	5.59E-02	6.25E-04
	X1303	malate	7.89E-01	8.70E-04
	X44656	isovalerate (i5:0)	5.30E-03	8.82E-04
	X53	glutamine	2.23E-02	9.51E-04
	X1561	alpha-tocopherol	1.16E-01	9.81E-04
	X18368	cys-gly, oxidized	5.00E-02	1.12E-03

X59	histidine	2.00E-02	1.30E-03
X52677	1-(1-enyl-palmitoyl)-2-linoleoyl-GPE (P-16:0/18:2)*	7.43E-02	1.37E-03
X35126	phenylacetylglutamine	2.22E-01	1.57E-03
X33953	N-acetylarginine	6.96E-02	1.67E-03
X32377	N-acetylneuraminate	6.01E-03	1.78E-03
X52634	palmitoyl-linoleoyl-glycerol (16:0/18:2) [2]*	1.61E-02	1.81E-03
X19503	stearoyl sphingomyelin (d18:1/18:0)	1.30E-01	1.86E-03
X54885	1-linoleoyl-GPG (18:2)*	8.00E-01	2.00E-03
X57473	sphingomyelin (d18:0/18:0, d19:0/17:0)*	4.97E-01	2.42E-03
X39592	S-methylcysteine	1.88E-01	2.94E-03
X35437	isobutyrylglycine	1.84E-01	3.11E-03
X54950	linoleoyl-docosahexaenoyl-glycerol (18:2/22:6) [2]*	3.79E-01	3.21E-03
X32620	glycolithocholate sulfate*	7.30E-01	3.30E-03
X42582	pyruvate	2.53E-01	3.31E-03
X57516	cerotoylcarnitine (C26)*	2.50E-02	3.61E-03
X36850	taurolithocholate 3-sulfate	7.44E-01	3.62E-03
X57479	sphingomyelin (d18:2/24:2)*	4.65E-02	3.83E-03
X45970	1-palmitoyl-GPG (16:0)*	7.21E-01	4.02E-03
X53223	palmitoleoylcarnitine (C16:1)*	6.93E-01	4.13E-03
X52468	1-stearoyl-2-linoleoyl-GPI (18:0/18:2)	1.09E-01	4.20E-03
X48841	p-cresol glucuronide*	1.38E-01	4.80E-03
X55	beta-alanine	2.80E-01	5.05E-03
X48492	behenoyl sphingomyelin (d18:1/22:0)*	2.39E-01	5.06E-03
X35637	cysteinylglycine	3.10E-02	5.20E-03
X18476	glycocholate	9.68E-01	5.21E-03
X53263	docosahexaenoylcholine	5.63E-01	5.63E-03

Legend: P_AMDControl - p-values from AMD/Control model; P_Stage2Eye - p-values from Stage+2Eye model

Supplementary Table 11. Metabolites differing significantly across all study groups using Boston (US) samples based on p values of Stage model.

Super Pathway	Sub Pathway	Metabolite	OR US	Pval US	Qval US	Qval Sig
Amino Acid	Methionine, Cysteine, SAM and Taurine Metabolism	cysteine s-sulfate	0.50	3.07E-06	5.56E-04	1
Amino Acid	Histidine Metabolism	histidine	0.51	1.75E-05	1.36E-03	1
Amino Acid	Alanine and Aspartate Metabolism	N-acetylasparagine	1.74	4.29E-04	1.42E-02	1
Amino Acid	Leucine, Isoleucine and Valine Metabolism	isovalerate (i5:0)	1.73	4.43E-04	1.42E-02	1
Amino Acid	Methionine, Cysteine, SAM and Taurine Metabolism	S-methylcysteine	0.64	2.93E-03	4.31E-02	1
Amino Acid	Tyrosine Metabolism	p-cresol glucuronide*	1.57	3.83E-03	4.97E-02	1
Amino Acid	Glutathione Metabolism	cysteinylglycine	0.68	7.14E-03	7.10E-02	0
Amino Acid	Glutamate Metabolism	beta-citrylglutamate	1.49	7.60E-03	7.26E-02	0
Amino Acid	Leucine, Isoleucine and Valine Metabolism	2-hydroxy-3-methylvalerate	1.48	9.16E-03	7.94E-02	0
Amino Acid	Methionine, Cysteine, SAM and Taurine Metabolism	S-methylcysteine sulfoxide	0.70	1.68E-02	1.23E-01	0
Amino Acid	Leucine, Isoleucine and Valine Metabolism	isobutyrylglycine	1.44	1.74E-02	1.25E-01	0
Amino Acid	Leucine, Isoleucine and Valine Metabolism	isovalerylglycine	1.40	2.51E-02	1.61E-01	0
Amino Acid	Leucine, Isoleucine and Valine Metabolism	N-acetylleucine	1.39	2.62E-02	1.66E-01	0
Amino Acid	Urea cycle; Arginine and Proline Metabolism	N2,N5-diacetylornithine	1.41	2.76E-02	1.67E-01	0
Amino Acid	Methionine, Cysteine, SAM and Taurine Metabolism	S-methylmethionine	0.72	2.94E-02	1.76E-01	0
Amino Acid	Leucine, Isoleucine and Valine Metabolism	alpha-hydroxyisovalerate	1.38	3.07E-02	1.77E-01	0
Amino Acid	Glutathione Metabolism	cys-gly, oxidized	0.73	3.38E-02	1.88E-01	0
Amino Acid	Urea cycle; Arginine and Proline Metabolism	N-acetylarginine	1.35	4.09E-02	2.09E-01	0
Amino Acid	Glutamate Metabolism	glutamine	0.74	4.13E-02	2.09E-01	0
Amino Acid	Methionine, Cysteine, SAM and Taurine Metabolism	N-acetyltaurine	1.39	4.15E-02	2.09E-01	0
Amino Acid	Urea cycle; Arginine and Proline Metabolism	N-acetylcitrulline	1.34	4.69E-02	2.22E-01	0
Carbohydrate	Aminosugar Metabolism	N-acetylglucosamine/N-acetylgalactosamine	1.55	6.86E-03	7.04E-02	0
Cofactors and Vitamins	Tocopherol Metabolism	alpha-tocopherol	2.01	4.75E-06	6.46E-04	1
Cofactors and Vitamins	Hemoglobin and Porphyrin Metabolism	biliverdin	1.47	1.39E-02	1.08E-01	0
Cofactors and Vitamins	Tocopherol Metabolism	gamma-tocopherol/beta-tocopherol	0.73	3.15E-02	1.77E-01	0
Energy	TCA Cycle	2-methylcitrate/homocitrate	1.41	3.06E-02	1.77E-01	0
Lipid	Lysoplasmalogen	1-(1-enyl-palmitoyl)-GPE (P-16:0)*	2.08	1.55E-06	5.25E-04	1
Lipid	Lysoplasmalogen	1-(1-enyl-stearoyl)-GPE (P-18:0)*	2.07	1.93E-06	5.25E-04	1
Lipid	Phosphatidylcholine (PC)	1-stearoyl-2-oleoyl-GPC (18:0/18:1)	0.50	7.99E-06	8.69E-04	1
Lipid	Lysoplasmalogen	1-(1-enyl-palmitoyl)-GPC (P-16:0)*	1.93	1.57E-05	1.36E-03	1
Lipid	Lysoplasmalogen	1-(1-enyl-oleoyl)-GPE (P-18:1)*	1.87	3.51E-05	2.39E-03	1
Lipid	Sphingomyelins	stearoyl sphingomyelin (d18:1/18:0)	1.83	4.67E-05	2.83E-03	1
Lipid	Fatty Acid Metabolism(Acyl Carnitine)	laurylcarnitine (C12)	0.57	1.84E-04	1.00E-02	1
Lipid	Diacylglycerol	oleoyl-oleoyl-glycerol (18:1/18:1) [2]*	0.59	2.75E-04	1.27E-02	1
Lipid	Diacylglycerol	oleoyl-linoleoyl-glycerol (18:1/18:2) [2]	0.58	3.01E-04	1.27E-02	1
Lipid	Phosphatidylcholine (PC)	1-stearoyl-2-linoleoyl-GPC (18:0/18:2)*	0.57	3.20E-04	1.27E-02	1
Lipid	Diacylglycerol	oleoyl-arachidonoyl-glycerol (18:1/20:4) [2]*	0.58	3.26E-04	1.27E-02	1
Lipid	Fatty Acid Metabolism(Acyl Carnitine)	oleoylcarnitine (C18:1)	0.58	3.75E-04	1.36E-02	1
Lipid	Phosphatidylcholine (PC)	1-palmitoyl-2-oleoyl-GPC (16:0/18:1)	0.59	4.73E-04	1.43E-02	1
Lipid	Diacylglycerol	oleoyl-linoleoyl-glycerol (18:1/18:2) [1]	0.60	5.74E-04	1.56E-02	1
Lipid	Phosphatidylinositol (PI)	1-stearoyl-2-arachidonoyl-GPI (18:0/20:4)	0.58	5.86E-04	1.56E-02	1
Lipid	Fatty Acid Metabolism(Acyl Carnitine)	myristoleoylcarnitine (C14:1)*	0.60	7.05E-04	1.74E-02	1
Lipid	Diacylglycerol	linoleoyl-arachidonoyl-glycerol (18:2/20:4) [2]*	0.61	7.58E-04	1.79E-02	1
Lipid	Sphingomyelins	sphingomyelin (d18:1/20:1, d18:2/20:0)*	1.67	8.57E-04	1.94E-02	1
Lipid	Phosphatidylcholine (PC)	1-stearoyl-2-arachidonoyl-GPC (18:0/20:4)	0.61	9.22E-04	2.01E-02	1
Lipid	Fatty Acid Metabolism(Acyl Carnitine)	palmitoylcarnitine (C16)	0.60	9.63E-04	2.01E-02	1
Lipid	Fatty Acid Metabolism(Acyl Carnitine)	myristoylcarnitine (C14)	0.61	1.06E-03	2.14E-02	1
Lipid	Fatty Acid Metabolism(Acyl Carnitine)	palmitoleoylcarnitine (C16:1)*	0.60	1.17E-03	2.27E-02	1

Lipid	Phosphatidylcholine (PC)	1-palmitoyl-2-linoleoyl-GPC (16:0/18:2)	0.62	1.35E-03	2.53E-02	1
Lipid	Phosphatidylcholine (PC)	1-linoleoyl-2-arachidonoyl-GPC (18:2/20:4n6)*	0.62	1.46E-03	2.64E-02	1
Lipid	Diacylglycerol	palmitoyl-linoleoyl-glycerol (16:0/18:2) [2]*	0.63	1.80E-03	3.08E-02	1
Lipid	Fatty Acid Metabolism(Acyl Carnitine)	linolenoylcarnitine (C18:3)*	0.63	1.86E-03	3.08E-02	1
Lipid	Plasmalogen	1-(1-enyl-palmitoyl)-2-linoleoyl-GPE (P-16:0/18:2)*	1.60	1.87E-03	3.08E-02	1
Lipid	Fatty Acid Metabolism(Acyl Carnitine)	dihomo-linolenoylcarnitine (C20:3n3 or 6)*	0.63	2.14E-03	3.43E-02	1
Lipid	Phosphatidylcholine (PC)	1-palmitoyl-2-stearoyl-GPC (16:0/18:0)	0.62	2.21E-03	3.44E-02	1
Lipid	Phosphatidylinositol (PI)	1-stearoyl-2-linoleoyl-GPI (18:0/18:2)	0.63	2.39E-03	3.61E-02	1
Lipid	Fatty Acid Metabolism(Acyl Carnitine)	decanoylcarnitine (C10)	0.66	3.64E-03	4.97E-02	1
Lipid	Fatty Acid Metabolism(Acyl Carnitine)	arachidonoylcarnitine (C20:4)	0.65	3.66E-03	4.97E-02	1
Lipid	Sphingomyelins	palmitoyl sphingomyelin (d18:1/16:0)	1.55	3.84E-03	4.97E-02	1
Lipid	Diacylglycerol	palmitoyl-oleoyl-glycerol (16:0/18:1) [2]*	0.65	4.27E-03	5.41E-02	0
Lipid	Diacylglycerol	oleoyl-oleoyl-glycerol (18:1/18:1) [1]*	0.66	4.71E-03	5.70E-02	0
Lipid	Fatty Acid Metabolism(Acyl Carnitine)	stearoylcarnitine (C18)	0.66	5.04E-03	5.92E-02	0
Lipid	Fatty Acid Metabolism(Acyl Carnitine)	octanoylcarnitine (C8)	0.67	5.25E-03	5.92E-02	0
Lipid	Lysophospholipid	1-palmitoyl-GPG (16:0)*	1.49	5.32E-03	5.92E-02	0
Lipid	Fatty Acid Metabolism(Acyl Carnitine)	ximenoylcarnitine (C26:1)*	0.66	5.34E-03	5.92E-02	0
Lipid	Sphingomyelins	sphingomyelin (d18:1/22:2, d18:2/22:1, d16:1/24:2)*	1.50	5.75E-03	6.25E-02	0
Lipid	Phosphatidylinositol (PI)	1-palmitoyl-2-oleoyl-GPI (16:0/18:1)*	0.66	6.36E-03	6.78E-02	0
Lipid	Plasmalogen	1-(1-enyl-palmitoyl)-2-arachidonoyl-GPE (P-16:0/20:4)*	1.49	7.17E-03	7.10E-02	0
Lipid	Sphingosines	sphingosine 1-phosphate	0.68	7.42E-03	7.21E-02	0
Lipid	Diacylglycerol	stearoyl-arachidonoyl-glycerol (18:0/20:4) [1]*	0.68	8.62E-03	7.89E-02	0
Lipid	Fatty Acid Metabolism(Acyl Carnitine)	linoleoylcarnitine (C18:2)*	0.68	8.78E-03	7.89E-02	0
Lipid	Diacylglycerol	oleoyl-arachidonoyl-glycerol (18:1/20:4) [1]*	0.68	8.84E-03	7.89E-02	0
Lipid	Lysophospholipid	1-linoleoyl-GPG (18:2)*	1.46	9.19E-03	7.94E-02	0
Lipid	Diacylglycerol	palmitoleoyl-linoleoyl-glycerol (16:1/18:2) [1]*	0.67	9.68E-03	8.23E-02	0
Lipid	Sphingomyelins	sphingomyelin (d18:2/24:2)*	1.44	1.04E-02	8.74E-02	0
Lipid	Fatty Acid Metabolism(Acyl Carnitine)	cerotoylcarnitine (C26)*	0.69	1.15E-02	9.45E-02	0
Lipid	Diacylglycerol	linoleoyl-arachidonoyl-glycerol (18:2/20:4) [1]*	0.69	1.19E-02	9.51E-02	0
Lipid	Diacylglycerol	palmitoyl-linoleoyl-glycerol (16:0/18:2) [1]*	0.69	1.19E-02	9.51E-02	0
Lipid	Fatty Acid, Monohydroxy	2-hydroxypalmitate	1.44	1.29E-02	1.02E-01	0
Lipid	Phosphatidylcholine (PC)	1-palmitoyl-2-arachidonoyl-GPC (16:0/20:4n6)	0.69	1.49E-02	1.14E-01	0
Lipid	Polyunsaturated Fatty Acid (n3 and n6)	arachidonate (20:4n6)	1.42	1.59E-02	1.19E-01	0
Lipid	Phosphatidylinositol (PI)	1-palmitoyl-2-arachidonoyl-GPI (16:0/20:4)*	0.68	1.60E-02	1.19E-01	0
Lipid	Fatty Acid Metabolism(Acyl Carnitine)	nervonoylcarnitine (C24:1)*	0.70	1.72E-02	1.25E-01	0
Lipid	Fatty Acid Metabolism(Acyl Carnitine)	dihomo-linoleoylcarnitine (C20:2)*	0.70	1.79E-02	1.26E-01	0
Lipid	Fatty Acid Metabolism(Acyl Carnitine)	eicosenoylcarnitine (C20:1)*	0.70	1.82E-02	1.26E-01	0
Lipid	Fatty Acid Metabolism(Acyl Carnitine)	docosahexaenoylcarnitine (C22:6)*	0.70	1.83E-02	1.26E-01	0
Lipid	Phosphatidylcholine (PC)	1-linoleoyl-2-linolenoyl-GPC (18:2/18:3)*	0.71	1.98E-02	1.35E-01	0
Lipid	Long Chain Fatty Acid	erucate (22:1n9)	1.42	2.08E-02	1.39E-01	0
Lipid	Fatty Acid Metabolism(Acyl Carnitine)	cis-4-decenoylcarnitine (C10:1)	0.73	2.09E-02	1.39E-01	0
Lipid	Phosphatidylcholine (PC)	1-palmitoyl-2-palmitoleoyl-GPC (16:0/16:1)*	0.71	2.38E-02	1.56E-01	0
Lipid	Lysophospholipid	1-palmitoyl-GPI (16:0)	1.38	2.72E-02	1.67E-01	0
Lipid	Diacylglycerol	linoleoyl-linoleoyl-glycerol (18:2/18:2) [1]*	0.72	2.75E-02	1.67E-01	0
Lipid	Phosphatidylcholine (PC)	1,2-dilinoleoyl-GPC (18:2/18:2)	0.73	2.75E-02	1.67E-01	0
Lipid	Androgenic Steroids	5alpha-androstan-3beta,17beta-diol disulfate	1.46	2.99E-02	1.77E-01	0
Lipid	Lysophospholipid	1-arachidonoyl-GPI (20:4)*	1.38	3.09E-02	1.77E-01	0
Lipid	Dihydrosphingomyelins	palmitoyl dihydrosphingomyelin (d18:0/16:0)*	1.37	3.14E-02	1.77E-01	0
Lipid	Polyunsaturated Fatty Acid (n3 and n6)	dihomo-linoleate (20:2n6)	1.36	3.73E-02	2.03E-01	0
Lipid	Phosphatidylcholine (PC)	1,2-dipalmitoyl-GPC (16:0/16:0)	0.73	3.91E-02	2.09E-01	0
Lipid	Lysophospholipid	1-linoleoyl-GPI (18:2)*	1.35	3.97E-02	2.09E-01	0

Lipid	Diacylglycerol	palmitoyl-arachidonoyl-glycerol (16:0/20:4) [2]*	0.74	4.06E-02	2.09E-01	0
Lipid	Phosphatidylinositol (PI)	1-palmitoyl-2-linoleoyl-GPI (16:0/18:2)	0.73	4.11E-02	2.09E-01	0
Lipid	Diacylglycerol	linoleoyl-docosahexaenoyl-glycerol (18:2/22:6) [2]*	0.72	4.14E-02	2.09E-01	0
Lipid	Polyunsaturated Fatty Acid (n3 and n6)	docosapentaenoate (n3 DPA; 22:5n3)	1.36	4.31E-02	2.11E-01	0
Lipid	Fatty Acid, Dicarboxylate	tetradecanedioate (C14-DC)	1.38	4.48E-02	2.17E-01	0
Lipid	Lysophospholipid	1-stearoyl-GPI (18:0)	1.33	4.63E-02	2.22E-01	0
Lipid	Fatty Acid, Monohydroxy	16-hydroxypalmitate	1.35	4.73E-02	2.22E-01	0
Lipid	Lysophospholipid	1-lignoceroyl-GPC (24:0)	0.75	4.74E-02	2.22E-01	0
Nucleotide	Pyrimidine Metabolism, Cytidine containing	cytidine	1.68	6.01E-04	1.56E-02	1
Nucleotide	Purine Metabolism, Adenine containing	adenosine	1.44	6.76E-03	7.04E-02	0
Nucleotide	Purine Metabolism, (Hypo)Xanthine/Inosine containing	allantoin	0.65	8.09E-03	7.59E-02	0
Nucleotide	Purine Metabolism, (Hypo)Xanthine/Inosine containing	inosine	1.37	2.51E-02	1.61E-01	0
Nucleotide	Purine Metabolism, (Hypo)Xanthine/Inosine containing	urate	0.72	4.23E-02	2.10E-01	0
Peptide	Acetylated Peptides	phenylacetylglutamine	1.53	3.19E-03	4.57E-02	1
Peptide	Gamma-glutamyl Amino Acid	gamma-glutamylglutamine	0.64	4.45E-03	5.50E-02	0
Peptide	Acetylated Peptides	4-hydroxyphenylacetylglutamine	1.37	3.57E-02	1.96E-01	0
Peptide	Gamma-glutamyl Amino Acid	gamma-glutamylhistidine	0.75	4.24E-02	2.10E-01	0

Legend: OR – Odds Ratio; Pval – P-value; Qval – q-value; US – United States; Qval_Sig – indicator for significance based on q-value.

Supplementary Table 12. Metabolites differing significantly across all study groups using Coimbra (Portugal) samples based on p values of Stage model.

Super Pathway	Sub Pathway	Metabolite	OR PT	Pval PT	Qval PT	Qval Sig
Amino Acid	Methionine, Cysteine, SAM and Taurine Metabolism	hypotaurine	1.68	3.81E-05	6.91E-03	1
Amino Acid	Glutamate Metabolism	glutamate	1.52	4.16E-04	2.43E-02	1
Amino Acid	Alanine and Aspartate Metabolism	aspartate	1.49	5.32E-04	2.43E-02	1
Amino Acid	Methionine, Cysteine, SAM and Taurine Metabolism	taurine	1.46	6.33E-04	2.43E-02	1
Amino Acid	Methionine, Cysteine, SAM and Taurine Metabolism	N-acetylmethionine	1.48	1.27E-03	3.83E-02	1
Amino Acid	Glutamate Metabolism	beta-citrylglutamate	1.40	3.39E-03	8.77E-02	0
Amino Acid	Methionine, Cysteine, SAM and Taurine Metabolism	S-adenosylhomocysteine (SAH)	1.38	7.16E-03	1.56E-01	0
Amino Acid	Urea cycle; Arginine and Proline Metabolism	trans-4-hydroxyproline	1.35	9.90E-03	1.92E-01	0
Amino Acid	Polyamine Metabolism	spermidine	1.34	1.04E-02	1.95E-01	0
Amino Acid	Tryptophan Metabolism	indoleacetylglutamine	0.75	1.19E-02	2.09E-01	0
Amino Acid	Alanine and Aspartate Metabolism	N-acetylasparagine	1.33	1.51E-02	2.35E-01	0
Amino Acid	Urea cycle; Arginine and Proline Metabolism	N-methylproline	0.78	2.69E-02	3.85E-01	0
Amino Acid	Glutamate Metabolism	N-acetylglutamate	1.30	3.25E-02	4.21E-01	0
Amino Acid	Glutamate Metabolism	carboxyethyl-GABA	0.78	3.73E-02	4.42E-01	0
Amino Acid	Tryptophan Metabolism	serotonin	1.25	4.46E-02	4.79E-01	0
Carbohydrate	Glycogen Metabolism	maltotriose	1.63	1.95E-05	5.30E-03	1
Carbohydrate	Glycogen Metabolism	maltose	1.58	6.09E-05	7.55E-03	1
Carbohydrate	Glycolysis, Gluconeogenesis, and Pyruvate Metabolism	pyruvate	1.48	9.95E-04	3.22E-02	1
Carbohydrate	Glycolysis, Gluconeogenesis, and Pyruvate Metabolism	1,5-anhydroglucitol (1,5-AG)	0.74	6.97E-03	1.56E-01	0
Carbohydrate	Aminosugar Metabolism	N-acetylneuraminate	1.39	8.32E-03	1.68E-01	0
Carbohydrate	Glycolysis, Gluconeogenesis, and Pyruvate Metabolism	lactate	1.28	4.48E-02	4.79E-01	0
Cofactors and Vitamins	Nicotinate and Nicotinamide Metabolism	nicotinamide	1.48	6.35E-04	2.43E-02	1
Cofactors and Vitamins	Vitamin B6 Metabolism	pyridoxate	1.31	2.37E-02	3.49E-01	0
Cofactors and Vitamins	Tocopherol Metabolism	gamma-CEHC	1.26	3.64E-02	4.42E-01	0
Energy	TCA Cycle	malate	1.31	2.99E-02	4.11E-01	0
Lipid	Phosphatidylserine (PS)	1-stearoyl-2-oleoyl-GPS (18:0/18:1)	1.66	7.20E-06	3.92E-03	1
Lipid	Phospholipid Metabolism	phosphoethanolamine	1.55	1.04E-04	8.05E-03	1
Lipid	Sphingosines	sphingosine 1-phosphate	1.51	2.13E-04	1.45E-02	1
Lipid	Eicosanoid	12-HETE	1.49	4.47E-04	2.43E-02	1
Lipid	Sphingolipid Synthesis	sphinganine	1.49	5.48E-04	2.43E-02	1
Lipid	Phospholipid Metabolism	choline phosphate	1.47	6.70E-04	2.43E-02	1
Lipid	Fatty Acid, Monohydroxy	14-HDoHE/17-HDoHE	1.42	1.58E-03	4.40E-02	1
Lipid	Sphingosines	sphingosine	1.43	1.62E-03	4.40E-02	1
Lipid	Lysophospholipid	1-oleoyl-GPE (18:1)	1.36	7.58E-03	1.59E-01	0
Lipid	Lysophospholipid	1-linoleoyl-GPE (18:2)*	1.34	1.14E-02	2.07E-01	0
Lipid	Lysoplasmalogen	1-(1-enyl-palmitoyl)-GPE (P-16:0)*	1.32	1.35E-02	2.26E-01	0
Lipid	Fatty Acid Metabolism(Acyl Carnitine)	dihomo-linolenoylcarnitine (C20:3n3 or 6)*	1.31	1.67E-02	2.52E-01	0
Lipid	Phosphatidylcholine (PC)	1,2-dipalmitoyl-GPC (16:0/16:0)	1.30	3.02E-02	4.11E-01	0
Lipid	Lysophospholipid	1-stearoyl-GPE (18:0)	1.28	3.10E-02	4.11E-01	0
Lipid	Sphingomyelins	lignoceroyl sphingomyelin (d18:1/24:0)	1.28	3.35E-02	4.24E-01	0
Lipid	Primary Bile Acid Metabolism	glycochenodeoxycholate	1.28	3.70E-02	4.42E-01	0
Lipid	Phosphatidylethanolamine (PE)	1-stearoyl-2-oleoyl-GPE (18:0/18:1)	1.26	4.11E-02	4.76E-01	0
Lipid	Fatty Acid Metabolism (Acyl Choline)	docosahexaenoylcholine	0.80	4.33E-02	4.79E-01	0
Lipid	Sphingomyelins	behenoyl sphingomyelin (d18:1/22:0)*	1.27	4.49E-02	4.79E-01	0
Lipid	Primary Bile Acid Metabolism	glycocholate	1.28	4.58E-02	4.79E-01	0
Lipid	Fatty Acid Metabolism(Acyl Carnitine)	dihomo-linoleoylcarnitine (C20:2)*	1.25	4.92E-02	5.03E-01	0
Lipid	Fatty Acid, Dicarboxylate	3-methyladipate	0.80	4.99E-02	5.03E-01	0
Nucleotide	Pyrimidine Metabolism, Orotate containing	dihydroorotate	0.64	7.84E-05	7.55E-03	1

Nucleotide	Purine Metabolism, (Hypo)Xanthine/Inosine containing	hypoxanthine	1.57	8.33E-05	7.55E-03	1
Nucleotide	Pyrimidine Metabolism, Cytidine containing	cytidine	1.48	1.01E-03	3.22E-02	1
Nucleotide	Purine Metabolism, Guanine containing	guanine	1.37	6.03E-03	1.43E-01	0
Nucleotide	Purine Metabolism, Adenine containing	adenosine	1.31	1.37E-02	2.26E-01	0
Nucleotide	Pyrimidine Metabolism, Uracil containing	uracil	1.31	1.51E-02	2.35E-01	0
Peptide	Dipeptide	isoleucylglycine	1.36	5.66E-03	1.40E-01	0

Legend: OR – Odds Ratio; Pval – P-value; Qval – q-value; PT– Portugal; Qval_Sig – indicator for significance based on q-value.

Supplementary Table 13. Metabolites differing significantly (p-value) of Stage model across all study groups identified on the meta-analysis of the 2 cohorts.

Super Pathway	Sub Pathway	Metabolite	OR PT	Pval PT	OR US	Pval US	OR Meta	Pval Meta	Qval Meta	Qval Sig
Amino Acid	Alanine and Aspartate Metabolism	N-acetylasparagine	1.33	1.51E-02	1.74	4.29E-04	1.45	4.56E-05	6.35E-03	1
Amino Acid	Glutamate Metabolism	beta-citrylglutamate	1.40	3.39E-03	1.49	7.60E-03	1.42	7.79E-05	7.11E-03	1
Amino Acid	Methionine, Cysteine, SAM and Taurine Metabolism	hypotaurine	1.68	3.81E-05	1.19	2.59E-01	1.49	7.84E-05	7.11E-03	1
Amino Acid	Methionine, Cysteine, SAM and Taurine Metabolism	taurine	1.46	6.33E-04	1.15	3.20E-01	1.34	9.25E-04	3.59E-02	1
Amino Acid	Methionine, Cysteine, SAM and Taurine Metabolism	N-acetylmethionine	1.48	1.27E-03	1.13	4.34E-01	1.34	2.46E-03	7.04E-02	0
Amino Acid	Histidine Metabolism	histidine	0.95	6.45E-01	0.51	1.75E-05	0.76	2.78E-03	7.28E-02	0
Amino Acid	Urea cycle; Arginine and Proline Metabolism	trans-4-hydroxyproline	1.35	9.90E-03	1.21	1.92E-01	1.29	4.52E-03	9.86E-02	0
Amino Acid	Alanine and Aspartate Metabolism	aspartate	1.49	5.32E-04	1.02	8.97E-01	1.31	4.81E-03	9.92E-02	0
Amino Acid	Methionine, Cysteine, SAM and Taurine Metabolism	S-adenosylhomocysteine (SAH)	1.38	7.16E-03	1.20	2.84E-01	1.31	5.38E-03	1.04E-01	0
Amino Acid	Tryptophan Metabolism	serotonin	1.25	4.46E-02	1.31	5.57E-02	1.26	5.79E-03	1.09E-01	0
Amino Acid	Leucine, Isoleucine and Valine Metabolism	N-acetylleucine	1.23	9.04E-02	1.39	2.62E-02	1.30	6.94E-03	1.18E-01	0
Amino Acid	Urea cycle; Arginine and Proline Metabolism	N-acetylarginine	1.21	1.03E-01	1.35	4.09E-02	1.26	1.10E-02	1.76E-01	0
Amino Acid	Glutamate Metabolism	glutamine	0.84	1.13E-01	0.74	4.13E-02	0.80	1.23E-02	1.92E-01	0
Amino Acid	Glycine, Serine and Threonine Metabolism	betaine	0.80	6.31E-02	0.83	2.00E-01	0.81	2.42E-02	2.63E-01	0
Amino Acid	Tyrosine Metabolism	N-acetyltyrosine	1.17	1.70E-01	1.33	5.90E-02	1.22	2.51E-02	2.63E-01	0
Amino Acid	Leucine, Isoleucine and Valine Metabolism	isobutyrylglycine	1.12	3.40E-01	1.44	1.74E-02	1.23	2.72E-02	2.74E-01	0
Amino Acid	Urea cycle; Arginine and Proline Metabolism	N-acetylcitrulline	1.15	2.29E-01	1.34	4.69E-02	1.21	3.02E-02	2.78E-01	0
Amino Acid	Urea cycle; Arginine and Proline Metabolism	N-methylproline	0.78	2.69E-02	0.91	4.98E-01	0.83	3.04E-02	2.78E-01	0
Amino Acid	Glutamate Metabolism	N-acetylglutamate	1.30	3.25E-02	1.12	4.42E-01	1.22	3.07E-02	2.78E-01	0
Amino Acid	Urea cycle; Arginine and Proline Metabolism	2-oxoarginine*	1.22	9.22E-02	1.21	1.78E-01	1.22	3.11E-02	2.78E-01	0
Amino Acid	Glutathione Metabolism	cys-gly, oxidized	0.89	2.93E-01	0.73	3.38E-02	0.83	3.32E-02	2.82E-01	0
Amino Acid	Leucine, Isoleucine and Valine Metabolism	isovalerate (i5:0)	0.99	9.54E-01	1.73	4.43E-04	1.23	3.55E-02	2.88E-01	0
Amino Acid	Methionine, Cysteine, SAM and Taurine Metabolism	S-methylmethionine	0.91	3.80E-01	0.72	2.94E-02	0.82	4.27E-02	3.08E-01	0
Amino Acid	Tyrosine Metabolism	p-cresol glucuronide*	1.03	7.91E-01	1.57	3.83E-03	1.18	4.79E-02	3.20E-01	0
Carbohydrate	Glycogen Metabolism	maltose	1.58	6.09E-05	1.17	2.65E-01	1.42	1.16E-04	8.93E-03	1
Carbohydrate	Glycogen Metabolism	maltotriose	1.63	1.95E-05	1.10	4.90E-01	1.43	1.44E-04	8.93E-03	1
Carbohydrate	Aminosugar Metabolism	N-acetylneuraminate	1.39	8.32E-03	1.09	6.02E-01	1.28	1.61E-02	2.10E-01	0
Carbohydrate	Glycolysis, Gluconeogenesis, and Pyruvate Metabolism	pyruvate	1.48	9.95E-04	0.90	4.56E-01	1.20	3.17E-02	2.78E-01	0
Carbohydrate	Aminosugar Metabolism	N-acetylglucosamine/N-acetylgalactosamine	1.07	6.26E-01	1.55	6.86E-03	1.23	4.15E-02	3.08E-01	0
Cofactors and Vitamins	Tocopherol Metabolism	alpha-tocopherol	1.07	5.34E-01	2.01	4.75E-06	1.44	1.00E-03	3.64E-02	1
Cofactors and Vitamins	Nicotinate and Nicotinamide Metabolism	nicotinamide	1.48	6.35E-04	0.92	6.00E-01	1.27	1.72E-02	2.10E-01	0
Energy	TCA Cycle	malate	1.31	2.99E-02	1.28	9.46E-02	1.30	6.14E-03	1.11E-01	0
Lipid	Lysoplasmalogen	1-(1-enyl-palmitoyl)-GPE (P-16:0)*	1.32	1.35E-02	2.08	1.55E-06	1.59	9.94E-07	5.41E-04	1
Lipid	Phospholipid Metabolism	phosphoethanolamine	1.55	1.04E-04	1.26	1.02E-01	1.42	4.67E-05	6.35E-03	1
Lipid	Lysoplasmalogen	1-(1-enyl-stearoyl)-GPE (P-18:0)*	1.13	2.64E-01	2.07	1.93E-06	1.46	1.48E-04	8.93E-03	1
Lipid	Phosphatidylserine (PS)	1-stearoyl-2-oleoyl-GPS (18:0/18:1)	1.66	7.20E-06	1.03	8.52E-01	1.43	2.47E-04	1.28E-02	1
Lipid	Lysoplasmalogen	1-(1-enyl-oleoyl)-GPE (P-18:1)*	1.17	1.56E-01	1.87	3.51E-05	1.43	2.60E-04	1.28E-02	1
Lipid	Eicosanoid	12-HETE	1.49	4.47E-04	1.16	2.92E-01	1.36	6.22E-04	2.60E-02	1
Lipid	Diacylglycerol	oleoyl-oleoyl-glycerol (18:1/18:1) [2]*	0.88	2.68E-01	0.59	2.75E-04	0.75	1.93E-03	5.83E-02	0
Lipid	Phospholipid Metabolism	choline phosphate	1.47	6.70E-04	1.07	6.28E-01	1.32	2.81E-03	7.28E-02	0
Lipid	Diacylglycerol	oleoyl-linoleoyl-glycerol (18:1/18:2) [1]	0.88	2.76E-01	0.60	5.74E-04	0.75	3.00E-03	7.42E-02	0
Lipid	Sphingomyelins	stearoyl sphingomyelin (d18:1/18:0)	1.05	6.59E-01	1.83	4.67E-05	1.32	4.53E-03	9.86E-02	0
Lipid	Diacylglycerol	oleoyl-linoleoyl-glycerol (18:1/18:2) [2]	0.92	4.47E-01	0.58	3.01E-04	0.75	4.93E-03	9.92E-02	0
Lipid	Plasmalogen	1-(1-enyl-palmitoyl)-2-arachidonoyl-GPE (P-16:0/20:4)*	1.16	1.81E-01	1.49	7.17E-03	1.27	6.90E-03	1.18E-01	0
Lipid	Diacylglycerol	oleoyl-oleoyl-glycerol (18:1/18:1) [1]*	0.87	2.31E-01	0.66	4.71E-03	0.78	7.46E-03	1.23E-01	0
Lipid	Fatty Acid Metabolism(Acyl Carnitine)	cerotoylcarnitine (C26)*	0.87	2.38E-01	0.69	1.15E-02	0.79	1.31E-02	1.93E-01	0
Lipid	Diacylglycerol	palmitoyl-linoleoyl-glycerol (16:0/18:2) [2]*	0.92	4.70E-01	0.63	1.80E-03	0.77	1.31E-02	1.93E-01	0

Lipid	Lysophospholipid	1-linoleoyl-GPG (18:2)*	1.13	2.86E-01	1.46	9.19E-03	1.23	1.48E-02	2.10E-01	0
Lipid	Sphingolipid Synthesis	sphinganine	1.49	5.48E-04	0.93	6.09E-01	1.28	1.54E-02	2.10E-01	0
Lipid	Fatty Acid Metabolism(Acyl Carnitine)	docosahexaenoylcarnitine (C22:6)*	0.87	2.28E-01	0.70	1.83E-02	0.80	1.65E-02	2.10E-01	0
Lipid	Dihydrosphingomyelins	sphingomyelin (d18:0/18:0, d19:0/17:0)*	1.24	6.41E-02	1.25	1.28E-01	1.25	1.66E-02	2.10E-01	0
Lipid	Phosphatidylinositol (PI)	1-stearoyl-2-linoleoyl-GPI (18:0/18:2)	0.92	5.07E-01	0.63	2.39E-03	0.78	1.72E-02	2.10E-01	0
Lipid	Fatty Acid Metabolism(Acyl Carnitine)	laurylcarnitine (C12)	0.99	9.13E-01	0.57	1.84E-04	0.81	1.77E-02	2.10E-01	0
Lipid	Fatty Acid Metabolism(Acyl Carnitine)	myristoleoylcarnitine (C14:1)*	0.96	7.05E-01	0.60	7.05E-04	0.80	1.77E-02	2.10E-01	0
Lipid	Lysoplasmalogen	1-(1-enyl-palmitoyl)-GPC (P-16:0)*	0.96	7.23E-01	1.93	1.57E-05	1.35	1.83E-02	2.11E-01	0
Lipid	Fatty Acid, Monohydroxy	14-HDoHE/17-HDoHE	1.42	1.58E-03	0.95	7.42E-01	1.26	2.15E-02	2.44E-01	0
Lipid	Diacylglycerol	palmitoyl-linoleoyl-glycerol (16:0/18:2) [1]*	0.90	3.71E-01	0.69	1.19E-02	0.80	2.47E-02	2.63E-01	0
Lipid	Fatty Acid Metabolism(Acyl Carnitine)	decanoylcarnitine (C10)	0.94	5.75E-01	0.66	3.64E-03	0.83	2.63E-02	2.70E-01	0
Lipid	Sphingosines	sphingosine	1.43	1.62E-03	0.92	5.87E-01	1.26	3.06E-02	2.78E-01	0
Lipid	Sphingomyelins	sphingomyelin (d18:2/24:2)*	1.10	4.61E-01	1.44	1.04E-02	1.22	3.16E-02	2.78E-01	0
Lipid	Diacylglycerol	oleoyl-arachidonoyl-glycerol (18:1/20:4) [2]*	1.01	9.40E-01	0.58	3.26E-04	0.83	3.25E-02	2.81E-01	0
Lipid	Diacylglycerol	linoleoyl-docosahexaenoyl-glycerol (18:2/22:6) [2]*	0.88	2.71E-01	0.72	4.14E-02	0.83	3.42E-02	2.82E-01	0
Lipid	Lysophospholipid	1-stearoyl-GPE (18:0)	1.28	3.10E-02	1.10	5.02E-01	1.21	3.42E-02	2.82E-01	0
Lipid	Diacylglycerol	palmitoleoyl-linoleoyl-glycerol (16:1/18:2) [1]*	0.93	5.20E-01	0.67	9.68E-03	0.82	3.65E-02	2.88E-01	0
Lipid	Diacylglycerol	palmitoyl-oleoyl-glycerol (16:0/18:1) [2]*	0.96	6.93E-01	0.65	4.27E-03	0.83	3.94E-02	3.06E-01	0
Lipid	Fatty Acid, Monohydroxy	9-hydroxystearate	1.19	1.35E-01	1.24	1.56E-01	1.21	4.03E-02	3.08E-01	0
Lipid	Lysophospholipid	1-palmitoyl-GPG (16:0)*	1.05	6.76E-01	1.49	5.32E-03	1.21	4.18E-02	3.08E-01	0
Lipid	Fatty Acid Metabolism(Acyl Carnitine)	octanoylcarnitine (C8)	0.96	6.89E-01	0.67	5.25E-03	0.85	4.30E-02	3.08E-01	0
Lipid	Sphingomyelins	palmitoyl sphingomyelin (d18:1/16:0)	1.04	7.54E-01	1.55	3.84E-03	1.19	4.38E-02	3.09E-01	0
Lipid	Plasmalogen	1-(1-enyl-palmitoyl)-2-linoleoyl-GPE (P-16:0/18:2)*	1.01	9.05E-01	1.60	1.87E-03	1.19	4.59E-02	3.17E-01	0
Lipid	Secondary Bile Acid Metabolism	glycolithocholate sulfate*	1.21	1.01E-01	1.18	2.57E-01	1.20	4.64E-02	3.17E-01	0
Lipid	Sphingomyelins	behenoyl sphingomyelin (d18:1/22:0)*	1.27	4.49E-02	1.10	5.11E-01	1.20	4.67E-02	3.17E-01	0
Lipid	Sphingomyelins	sphingomyelin (d18:1/20:1, d18:2/20:0)*	0.99	9.36E-01	1.67	8.57E-04	1.22	4.83E-02	3.20E-01	0
Nucleotide	Pyrimidine Metabolism, Cytidine containing	cytidine	1.48	1.01E-03	1.68	6.01E-04	1.52	2.60E-06	7.07E-04	1
Nucleotide	Purine Metabolism, Adenine containing	adenosine	1.31	1.37E-02	1.44	6.76E-03	1.35	3.10E-04	1.41E-02	1
Nucleotide	Purine Metabolism, (Hypo)Xanthine/Inosine containing	hypoxanthine	1.57	8.33E-05	1.04	8.01E-01	1.37	1.09E-03	3.69E-02	1
Nucleotide	Pyrimidine Metabolism, Orotate containing	dihydroorotate	0.64	7.84E-05	1.00	9.76E-01	0.75	1.67E-03	5.35E-02	0
Nucleotide	Purine Metabolism, (Hypo)Xanthine/Inosine containing	inosine	1.24	5.17E-02	1.37	2.51E-02	1.24	3.62E-03	8.57E-02	0
Nucleotide	Purine Metabolism, Adenine containing	adenine	1.15	2.05E-01	1.31	5.45E-02	1.17	2.94E-02	2.78E-01	0
Nucleotide	Purine Metabolism, Guanine containing	guanine	1.37	6.03E-03	0.97	8.16E-01	1.23	4.23E-02	3.08E-01	0
Peptide	Acetylated Peptides	phenylacetylglutamine	1.07	5.69E-01	1.53	3.19E-03	1.21	2.42E-02	2.63E-01	0
Peptide	Dipeptide	isoleucylglycine	1.36	5.66E-03	0.98	8.73E-01	1.23	3.65E-02	2.88E-01	0

Legend: OR – Odds Ratio; Pval – P-value; Qval – q-value; PT– Portugal; US – United States; Meta – Meta analysis; Qval_Sig - indicator for significance based on q-value.

Supplementary Table 14. List of the metabolites belonging to metabolite sets and their p-values of AMD/Control, Stage and Stage+2Eye models from Boston cohort.

Met_Set	Met_ID	Metabolite	P_AMDControl	P_Stage	P_Stage2Eye
Set B	X22176	cysteine s-sulfate	1.60E-05	3.07E-06	1.00E-06
Set D	X39270	1-(1-enyl-palmitoyl)-GPE (P-16:0)*	2.98E-04	1.55E-06	1.00E-06
	X39271	1-(1-enyl-stearoyl)-GPE (P-18:0)*	6.67E-04	1.93E-06	1.00E-06
	X1561	alpha-tocopherol	1.94E-03	4.75E-06	1.00E-06
	X52438	1-stearoyl-2-oleoyl-GPC (18:0/18:1)	4.28E-03	7.99E-06	1.00E-06
	X52474	1-(1-enyl-palmitoyl)-GPC (P-16:0)*	8.69E-03	1.57E-05	1.00E-06
	X59	histidine	2.77E-02	1.75E-05	1.00E-06
	X44621	1-(1-enyl-oleoyl)-GPE (P-18:1)*	2.36E-03	3.51E-05	1.00E-06
	X19503	stearoyl sphingomyelin (d18:1/18:0)	1.91E-01	4.67E-05	1.00E-06
	X34534	laurylcarnitine (C12)	1.08E-02	1.84E-04	1.00E-06
	X54946	oleoyl-oleoyl-glycerol (18:1/18:1) [2]*	3.51E-02	2.75E-04	1.00E-06
	X54961	oleoyl-arachidonoyl-glycerol (18:1/20:4) [2]*	7.13E-03	3.26E-04	1.00E-06
	X35160	oleoylcarnitine (C18:1)	1.27E-02	3.75E-04	1.00E-06
	X33942	N-acetylasparagine	4.21E-03	4.29E-04	1.00E-06
	X44656	isovalerate (i5:0)	1.24E-02	4.43E-04	1.00E-06
	X52449	1-stearoyl-2-arachidonoyl-GPI (18:0/20:4)	1.67E-02	5.86E-04	1.00E-06
	X514	cytidine	1.35E-03	6.01E-04	1.00E-06
	X48182	myristoleoylcarnitine (C14:1)*	1.59E-02	7.05E-04	1.00E-06
	X44681	palmitoylcarnitine (C16)	7.80E-03	9.63E-04	1.00E-06
	X53223	palmitoleoylcarnitine (C16:1)*	1.50E-02	1.17E-03	1.00E-06
	X46798	oleoyl-linoleoyl-glycerol (18:1/18:2) [1]	4.20E-02	5.74E-04	1.00E-05
	X52677	1-(1-enyl-palmitoyl)-2-linoleoyl-GPE (P-16:0/18:2)*	6.16E-04	1.87E-03	1.00E-05
	X46799	oleoyl-linoleoyl-glycerol (18:1/18:2) [2]	1.49E-02	3.01E-04	2.00E-05
	X52452	1-stearoyl-2-linoleoyl-GPC (18:0/18:2)*	9.58E-02	3.20E-04	2.00E-05
	X52461	1-palmitoyl-2-oleoyl-GPC (16:0/18:1)	6.37E-03	4.73E-04	2.00E-05
	X57521	dihomo-linolenoylcarnitine (C20:3n3 or 6)*	4.43E-02	2.14E-03	2.00E-05
	X39592	S-methylcysteine	1.50E-02	2.93E-03	2.00E-05
	X48491	sphingomyelin (d18:1/20:1, d18:2/20:0)*	5.18E-02	8.57E-04	4.00E-05
	X33952	myristoylcarnitine (C14)	6.15E-03	1.06E-03	5.00E-05
	X52710	1-linoleoyl-2-arachidonoyl-GPC (18:2/20:4n6)*	1.22E-02	1.46E-03	6.00E-05
	X42450	1-stearoyl-2-arachidonoyl-GPC (18:0/20:4)	1.14E-02	9.22E-04	7.00E-05
	X37506	palmitoyl sphingomyelin (d18:1/16:0)	2.64E-01	3.84E-03	8.00E-05
	X42446	1-palmitoyl-2-linoleoyl-GPC (16:0/18:2)	6.38E-02	1.35E-03	1.10E-04
	X57518	arachidonoylcarnitine (C20:4)	5.12E-02	3.66E-03	1.10E-04
	X54956	linoleoyl-arachidonoyl-glycerol (18:2/20:4) [2]*	3.05E-02	7.58E-04	1.20E-04
	X52634	palmitoyl-linoleoyl-glycerol (16:0/18:2) [2]*	3.03E-02	1.80E-03	1.60E-04
	X52616	1-palmitoyl-2-stearoyl-GPC (16:0/18:0)	1.01E-01	2.21E-03	1.70E-04
	X57511	linolenoylcarnitine (C18:3)*	6.81E-02	1.86E-03	1.80E-04
	X35126	phenylacetylglutamine	1.47E-01	3.19E-03	2.20E-04
	X33941	decanoylcarnitine (C10)	1.34E-01	3.64E-03	2.20E-04
	X52468	1-stearoyl-2-linoleoyl-GPI (18:0/18:2)	1.14E-01	2.39E-03	2.50E-04
	X48841	p-cresol glucuronide*	1.63E-01	3.83E-03	1.62E-03
Set E	X555	adenosine	6.90E-04	6.76E-03	7.00E-05
	X52673	1-(1-enyl-palmitoyl)-2-arachidonoyl-GPE (P-16:0/20:4)*	3.72E-03	7.17E-03	9.00E-05
	X36746	2-hydroxy-3-methylvalerate	1.50E-01	9.16E-03	1.00E-04
	X57517	ximenoylcarnitine (C26:1)*	4.29E-02	5.34E-03	1.10E-04
	X2137	biliverdin	3.05E-02	1.39E-02	1.60E-04
	X46539	N-acetylglucosamine/N-acetylgalactosamine	3.58E-01	6.86E-03	2.20E-04

X45970	1-palmitoyl-GPG (16:0)*	2.10E-01	5.32E-03	2.60E-04
X52669	1-palmitoyl-2-oleoyl-GPI (16:0/18:1)*	1.29E-01	6.36E-03	3.30E-04
X34409	stearoylcarnitine (C18)	2.29E-02	5.04E-03	3.40E-04
X35637	cysteinylglycine	6.33E-03	7.14E-03	3.40E-04
X54923	beta-citrylglutamate	5.40E-02	7.60E-03	3.50E-04
X57477	sphingomyelin (d18:1/22:2, d18:2/22:1, d16:1/24:2)*	4.71E-01	5.75E-03	3.50E-04
X2730	gamma-glutamylglutamine	3.12E-01	4.45E-03	3.70E-04
X54942	palmitoyl-oleoyl-glycerol (16:0/18:1) [2]*	4.67E-02	4.27E-03	3.90E-04
X34445	sphingosine 1-phosphate	1.86E-01	7.42E-03	4.80E-04
X54885	1-linoleoyl-GPG (18:2)*	1.90E-01	9.19E-03	5.70E-04
X33936	octanoylcarnitine (C8)	1.50E-01	5.25E-03	6.10E-04
X54945	oleoyl-oleoyl-glycerol (18:1/18:1) [1]*	6.11E-02	4.71E-03	6.50E-04
X38125	4-cholesten-3-one	7.40E-02	5.83E-02	6.80E-04
X57450	stearoyl-arachidonoyl-glycerol (18:0/20:4) [1]*	1.29E-01	8.62E-03	7.10E-04
X57523	docosahexaenoylcarnitine (C22:6)*	2.30E-01	1.83E-02	7.20E-04
X1587	N-acetylleucine	3.37E-01	2.62E-02	7.30E-04
X43378	S-methylcysteine sulfoxide	1.06E-01	1.68E-02	7.90E-04
X46223	linoleoylcarnitine (C18:2)*	1.58E-01	8.78E-03	1.12E-03
X57531	nervonoylcarnitine (C24:1)*	2.78E-02	1.72E-02	1.12E-03
X57519	eicosenoylcarnitine (C20:1)*	8.31E-02	1.82E-02	1.24E-03
X33937	alpha-hydroxyisovalerate	6.74E-01	3.07E-02	1.37E-03
X33953	N-acetylarginine	3.62E-01	4.09E-02	1.40E-03
X57479	sphingomyelin (d18:2/24:2)*	1.70E-01	1.04E-02	1.44E-03
X54967	palmitoleoyl-linoleoyl-glycerol (16:1/18:2) [1]*	6.93E-02	9.68E-03	1.54E-03
X57520	dihomo-linoleoylcarnitine (C20:2)*	1.67E-01	1.79E-02	1.56E-03
X35437	isobutyrylglycine	3.94E-01	1.74E-02	1.67E-03
X35107	isovalerylglycine	2.27E-01	2.51E-02	1.72E-03
X1123	inosine	9.69E-03	2.51E-02	1.79E-03
X57516	cerotoylcarnitine (C26)*	1.22E-01	1.15E-02	1.79E-03
X49617	1-lignoceroyl-GPC (24:0)	1.51E-01	4.74E-02	1.82E-03
X35675	2-hydroxypalmitate	8.34E-01	1.29E-02	2.06E-03
X52282	2-methylcitrate/homocitrate	6.15E-01	3.06E-02	2.10E-03
X52470	1-palmitoyl-2-palmitoleoyl-GPC (16:0/16:1)*	2.59E-02	2.38E-02	2.17E-03
X52633	palmitoyl-linoleoyl-glycerol (16:0/18:2) [1]*	8.58E-02	1.19E-02	2.21E-03
X54960	oleoyl-arachidonoyl-glycerol (18:1/20:4) [1]*	7.18E-02	8.84E-03	2.29E-03
X1110	arachidonate (20:4n6)	5.55E-01	1.59E-02	2.46E-03
X52462	1-palmitoyl-2-arachidonoyl-GPC (16:0/20:4n6)	2.74E-02	1.49E-02	2.55E-03
X52467	1-palmitoyl-2-arachidonoyl-GPI (16:0/20:4)*	7.90E-02	1.60E-02	2.55E-03
X35305	1-palmitoyl-GPI (16:0)	1.73E-01	2.72E-02	2.65E-03
X36594	1-linoleoyl-GPI (18:2)*	1.75E-01	3.97E-02	2.78E-03
X52434	palmitoyl dihydrosphingomyelin (d18:0/16:0)*	4.15E-01	3.14E-02	3.39E-03
X48434	N-acetylcitrulline	7.14E-01	4.69E-02	3.83E-03
X52473	gamma-tocopherol/beta-tocopherol	2.66E-02	3.15E-02	4.12E-03
X53176	1-linoleoyl-2-linolenoyl-GPC (18:2/18:3)*	2.29E-01	1.98E-02	4.82E-03
X1552	erucate (22:1n9)	6.60E-01	2.08E-02	5.05E-03
X34214	1-arachidonoyl-GPI (20:4)*	5.13E-01	3.09E-02	5.06E-03
X1107	allantoin	1.05E-01	8.09E-03	5.15E-03
X43591	N2,N5-diacetylornithine	9.60E-01	2.76E-02	5.19E-03
X37190	5alpha-androstan-3beta,17beta-diol disulfate	4.72E-01	2.99E-02	5.44E-03
X2342	serotonin	2.67E-02	5.57E-02	5.54E-03
X38178	cis-4-decenoylcarnitine (C10:1)	2.73E-01	2.09E-02	5.78E-03

X53	glutamine	4.81E-01	4.13E-02	6.07E-03
X18368	cys-gly, oxidized	3.61E-02	3.38E-02	6.55E-03
X55017	4-hydroxyphenylacetylglutamine	6.43E-01	3.57E-02	6.61E-03
X19130	1,2-dipalmitoyl-GPC (16:0/16:0)	7.67E-02	3.91E-02	6.78E-03
X19324	1-stearoyl-GPI (18:0)	3.49E-01	4.63E-02	6.80E-03
X57529	docosapentaenoylcarnitine (C22:5n3)*	2.15E-01	6.88E-02	7.26E-03
X1418	5,6-dihydrothymine	6.74E-02	5.79E-02	7.39E-03
X554	adenine	4.38E-02	5.45E-02	7.52E-03
X57512	margaroylcarnitine (C17)*	1.54E-02	5.02E-02	7.59E-03
X18245	gamma-glutamylhistidine	9.09E-02	4.24E-02	7.60E-03
X54955	linoleoyl-arachidonoyl-glycerol (18:2/20:4) [1]*	7.82E-02	1.19E-02	7.98E-03
X32390	N-acetyltyrosine	8.01E-01	5.90E-02	8.54E-03
X54950	linoleoyl-docosahexaenoyl-glycerol (18:2/22:6) [2]*	1.76E-01	4.14E-02	9.49E-03
X52603	1,2-dilinoleoyl-GPC (18:2/18:2)	4.38E-01	2.75E-02	9.55E-03
X36751	N2-acetyllysine	4.73E-01	5.89E-02	9.89E-03

Legend: P_AMDControl - p-values from AMD/Control model; P_Stage - p-values from Stage model; P_Stage2Eye - p-values from Stage+2Eye model

Supplementary Table 15. List of the metabolites belonging to metabolite sets and their p-values of AMD/Control, Stage and Stage+2Eye models from Portuguese cohort.

Met_Set	Met_ID	Metabolite	P_AMDControl	P_Stage	P_Stage2Eye
Set A	X32352	guanine	5.99E-07	6.03E-03	4.64E-03
	X54923	beta-citrylglutamate	1.37E-05	3.39E-03	3.40E-03
	X555	adenosine	6.33E-05	1.37E-02	3.12E-03
	X1123	inosine	1.54E-04	5.17E-02	8.18E-02
	X42382	S-adenosylhomocysteine (SAH)	3.91E-04	7.16E-03	5.91E-03
	X2342	serotonin	6.20E-04	4.46E-02	1.71E-02
	X32412	butyrylcarnitine (C4)	1.69E-03	4.61E-01	5.67E-01
Set B	X19265	1-stearoyl-2-oleoyl-GPS (18:0/18:1)	2.94E-10	7.20E-06	1.00E-06
	X44688	maltotriose	2.44E-07	1.95E-05	1.00E-06
	X590	hypotaurine	4.69E-04	3.81E-05	1.00E-06
	X601	dihydroorotate	7.52E-04	7.84E-05	1.00E-06
	X3127	hypoxanthine	3.58E-05	8.33E-05	1.00E-05
	X1600	phosphoethanolamine	2.36E-08	1.04E-04	2.00E-05
	X15586	maltose	1.62E-06	6.09E-05	3.00E-05
Set C	X32377	N-acetylneuraminate	8.79E-05	8.32E-03	7.00E-04
	X40008	isoleucylglycine	1.25E-04	5.66E-03	2.23E-03
Set D	X42582	pyruvate	8.07E-02	9.95E-04	1.00E-05
	X57	glutamate	3.32E-03	4.16E-04	3.00E-05
Set E	X20675	1,5-anhydroglucitol (1,5-AG)	7.89E-02	6.97E-03	4.00E-05
	X36600	1-linoleoyl-GPE (18:2)*	5.48E-01	1.14E-02	1.37E-03
	X42087	indoleacetylglutamine	6.77E-02	1.19E-02	1.46E-03
	X32306	trans-4-hydroxyproline	8.08E-02	9.90E-03	2.09E-03
	X53263	docosahexaenoylcholine	2.28E-01	4.33E-02	2.30E-03

Legend: P_AMDControl - p-values from AMD/Control model; P_Stage - p-values from Stage model; P_Stage2Eye - p-values from Stage+2Eye model

Supplementary Table 16. List of the metabolites belonging to metabolite sets and their p-values of AMD/Control, Stage and Stage+2Eye models from the meta analysis.

Met_Set	Met_ID	Metabolite	P_AMDControl	P_Stage	P_Stage2Eye
Set A	X32352	guanine	8.16E-07	4.23E-02	4.70E-02
	X17747	sphingosine	2.26E-06	3.06E-02	4.24E-02
	X17747	14-HDoHE/17-HDoHE	3.87E-05	2.15E-02	3.42E-02
	X17769	sphinganine	3.05E-04	1.54E-02	1.68E-02
	X594	nicotinamide	7.87E-04	1.72E-02	1.65E-02
	X40008	isoleucylglycine	1.15E-03	3.65E-02	4.31E-02
	X554	adenine	2.13E-03	2.94E-02	4.20E-02
Set B	X514	cytidine	2.93E-07	2.60E-06	1.96E-08
	X33942	N-acetylasparagine	3.22E-04	4.56E-05	6.10E-07
	X39270	1-(1-enyl-palmitoyl)-GPE (P-16:0)*	1.12E-05	9.94E-07	9.86E-07
	X590	hypotaurine	2.57E-04	7.84E-05	1.21E-06
	X1600	phosphoethanolamine	2.44E-08	4.67E-05	1.62E-06
	X555	adenosine	1.60E-07	3.10E-04	1.84E-06
	X44688	maltotriose	1.31E-07	1.44E-04	6.19E-06
	X54923	beta-citrylglutamate	3.86E-06	7.79E-05	6.67E-06
	X15586	maltose	4.14E-06	1.16E-04	3.17E-05
	X19265	1-stearoyl-2-oleoyl-GPS (18:0/18:1)	1.80E-08	2.47E-04	8.78E-05
	X44621	1-(1-enyl-oleoyl)-GPE (P-18:1)*	1.41E-03	2.60E-04	2.83E-04
	X39271	1-(1-enyl-stearoyl)-GPE (P-18:0)*	1.81E-03	1.48E-04	3.08E-04
	X2125	taurine	9.59E-07	9.25E-04	3.51E-04
	X37536	12-HETE	4.03E-05	6.22E-04	7.55E-04
	X3127	hypoxanthine	8.43E-05	1.09E-03	7.75E-04
Set C	X2342	serotonin	4.84E-05	5.79E-03	3.40E-04
	X443	aspartate	1.02E-04	4.81E-03	5.93E-04
	X1589	N-acetylmethionine	9.74E-05	2.46E-03	8.60E-04
	X34396	choline phosphate	1.42E-07	2.81E-03	9.17E-04
	X1123	inosine	4.74E-06	3.62E-03	1.01E-03
	X42382	S-adenosylhomocysteine (SAH)	6.43E-04	5.38E-03	1.68E-03
Set D	X1561	alpha-tocopherol	1.16E-01	1.00E-03	9.81E-04
Set E	X54946	oleoyl-oleoyl-glycerol (18:1/18:1) [2]*	1.37E-02	1.93E-03	6.96E-06
	X46798	oleoyl-linoleoyl-glycerol (18:1/18:2) [1]	2.27E-02	3.00E-03	4.01E-05
	X48182	myristoleoylcarnitine (C14:1)*	2.62E-01	1.77E-02	6.54E-05
	X601	dihydroorotate	5.35E-03	1.67E-03	9.11E-05
	X34534	laurylcarnitine (C12)	1.88E-01	1.77E-02	1.18E-04
	X46799	oleoyl-linoleoyl-glycerol (18:1/18:2) [2]	2.28E-02	4.93E-03	1.68E-04
	X33941	decanoylcarnitine (C10)	1.89E-01	2.63E-02	1.77E-04
	X1587	N-acetylleucine	1.58E-01	6.94E-03	2.69E-04
	X54945	oleoyl-oleoyl-glycerol (18:1/18:1) [1]*	1.79E-02	7.46E-03	2.87E-04
	X57523	docosahexaenoylcarnitine (C22:6)*	9.95E-01	1.65E-02	3.42E-04
	X52673	1-(1-enyl-palmitoyl)-2-arachidonoyl-GPE (P-16:0/20:4)*	2.95E-02	6.90E-03	3.70E-04
	X55072	2-oxoarginine*	2.90E-01	3.11E-02	4.45E-04
	X54961	oleoyl-arachidonoyl-glycerol (18:1/20:4) [2]*	1.59E-02	3.25E-02	4.74E-04
	X33936	octanoylcarnitine (C8)	2.60E-01	4.30E-02	5.74E-04
	X32306	trans-4-hydroxyproline	5.59E-02	4.52E-03	6.25E-04
	X1303	malate	7.89E-01	6.14E-03	8.70E-04
	X44656	isovalerate (i5:0)	5.30E-03	3.55E-02	8.82E-04
	X53	glutamine	2.23E-02	1.23E-02	9.51E-04
	X18368	cys-gly, oxidized	5.00E-02	3.32E-02	1.12E-03

X59	histidine	2.00E-02	2.78E-03	1.30E-03
X52677	1-(1-enyl-palmitoyl)-2-linoleoyl-GPE (P-16:0/18:2)*	7.43E-02	4.59E-02	1.37E-03
X35126	phenylacetylglutamine	2.22E-01	2.42E-02	1.57E-03
X33953	N-acetylarginine	6.96E-02	1.10E-02	1.67E-03
X32377	N-acetylneuraminate	6.01E-03	1.61E-02	1.78E-03
X52634	palmitoyl-linoleoyl-glycerol (16:0/18:2) [2]*	1.61E-02	1.31E-02	1.81E-03
X19503	stearoyl sphingomyelin (d18:1/18:0)	1.30E-01	4.53E-03	1.86E-03
X54885	1-linoleoyl-GPG (18:2)*	8.00E-01	1.48E-02	2.00E-03
X57473	sphingomyelin (d18:0/18:0, d19:0/17:0)*	4.97E-01	1.66E-02	2.42E-03
X39592	S-methylcysteine	1.88E-01	8.30E-02	2.94E-03
X35437	isobutyrylglycine	1.84E-01	2.72E-02	3.11E-03
X54950	linoleoyl-docosahexaenoyl-glycerol (18:2/22:6) [2]*	3.79E-01	3.42E-02	3.21E-03
X32620	glycolithocholate sulfate*	7.30E-01	4.64E-02	3.30E-03
X42582	pyruvate	2.53E-01	3.17E-02	3.31E-03
X57516	cerotoylcarnitine (C26)*	2.50E-02	1.31E-02	3.61E-03
X36850	taurolithocholate 3-sulfate	7.44E-01	8.39E-02	3.62E-03
X57479	sphingomyelin (d18:2/24:2)*	4.65E-02	3.16E-02	3.83E-03
X45970	1-palmitoyl-GPG (16:0)*	7.21E-01	4.18E-02	4.02E-03
X53223	palmitoleoylcarnitine (C16:1)*	6.93E-01	1.04E-01	4.13E-03
X52468	1-stearoyl-2-linoleoyl-GPI (18:0/18:2)	1.09E-01	1.72E-02	4.20E-03
X48841	p-cresol glucuronide*	1.38E-01	4.79E-02	4.80E-03
X55	beta-alanine	2.80E-01	7.38E-02	5.05E-03
X48492	behenoyl sphingomyelin (d18:1/22:0)*	2.39E-01	4.67E-02	5.06E-03
X35637	cysteinylglycine	3.10E-02	9.62E-02	5.20E-03
X18476	glycocholate	9.68E-01	6.36E-02	5.21E-03
X53263	docosahexaenoylcholine	5.63E-01	8.86E-02	5.63E-03

Legend: P_AMDControl - p-values from AMD/Control model; P_Stage - p-values from Stage model; P_Stage2Eye - p-values from Stage+2Eye model

Supplementary Table 17. Area under the curve (AUC) for predictive models for AMD including clinical covariates and significant metabolites identified in the AMD/Control, Stage, Stage+2Eye or multivariate cumulative logistic models.

Data	Model	AUC	AUC_CI_L	AUC_CI_U	Nz_sig	Nz_final	Pval
Boston	Baseline	0.645	0.540	0.749	4.0	.	
	All-Met+EN	0.703	0.602	0.803	544.0	17.2	2.87E-02
	AMD/Control	0.691	0.596	0.786	53.0	17.7	2.44E-01
	Stage	0.730	0.646	0.815	108.0	13.8	4.06E-02
	Stage+2Eye	0.747	0.665	0.829	169.5	14.2	1.06E-02
	Multi.Logit	0.748	0.666	0.829	98.1	14.7	1.01E-02
Portugal	Baseline	0.759	0.697	0.821	4.0	.	
	All-Met+EN	0.810	0.758	0.862	544.0	15.3	2.27E-04
	AMD/Control+EN	0.826	0.775	0.878	57.8	15.1	7.79E-03
	Stage+EN	0.834	0.782	0.886	50.9	16.0	1.26E-03
	Stage+2Eye+EN	0.850	0.803	0.898	87.1	18.6	2.70E-04
	Multi.Logit+EN	0.861	0.813	0.908	45.4	20.2	7.69E-05
Combined	Baseline	0.725	0.671	0.779	4.0	.	
	All-Met+EN	0.745	0.692	0.797	544.0	25.5	1.36E-01
	AMD/Control	0.789	0.738	0.840	63.7	11.8	2.07E-04
	Stage	0.791	0.741	0.841	77.4	15.5	1.65E-04
	Stage+2Eye	0.815	0.771	0.860	140.6	16.8	3.74E-06
	Multi.Logit	0.791	0.741	0.842	76.4	16.7	1.99E-04

Legend: AUC_CI_L – Lower bound of 95% confidence interval of AUC; AUC_CI_L – Upper bound of 95% confidence interval of AUC;
Pred R2 - prediction accuracy; MSE - mean squared error; slope - slope of regression of AMD status on the fitted probabilities for AMD
NZ_Sig – number of non-zero significant metabolites selected by logistic or permutation based logistic regression models;
NZ_Final – number of non-zero metabolites in the final model ; Pval – p-value for the difference with baseline model;
Baseline - baseline model including only clinical covariates;
All-Met+EN- all metabolites plus elastic net model including baseline + metabolites selected using elastic net regression with all metabolites;
AMD/Control - logistic model including baseline + metabolites identified in the logistic regression;
Stage - cumulative logistic model including baseline + metabolites identified in the cumulative logistic regression;
Stage+2Eye - cumulative logistic model with permutation including baseline + metabolites identified in the permutation-based cumulative logistic regression;
Multi.Logit - multivariate cumulative logistic model including baseline + metabolites identified in the multivariate cumulative logistic regression

We further evaluated the predictive performance of regular cumulative logistic model for AMD stages of each subject (Stage model) and multivariate cumulative logistic model for AMD stages of both eyes (Supplementary Table 12). Stage model performed better than AMD/Control model as it used AMD stages, but worse than Stage+2Eye model as it used only AMD stages of each subject rather than those of each eye. Prediction results of multivariate cumulative logistic model is comparable to those of Stage+2Eye model as both models utilized the same information on AMD stages for both eyes.
